# Luminescent Anion Sensing by Transition‐Metal Dipyridylbenzene Complexes Incorporated into Acyclic, Macrocyclic and Interlocked Hosts

**DOI:** 10.1002/chem.202000661

**Published:** 2020-04-01

**Authors:** Richard C. Knighton, Sophie Dapin, Paul D. Beer

**Affiliations:** ^1^ Department of Chemistry University of Oxford Mansfield Road Oxford OX1 3TA UK

**Keywords:** [2]rotaxane, dipyridylbenzene, platinum, ruthenium, sensing

## Abstract

A series of novel acyclic, macrocyclic and mechanically interlocked luminescent anion sensors have been prepared by incorporation of the isophthalamide motif into dipyridylbenzene to obtain cyclometallated complexes of platinum(II) and ruthenium(II). Both the acyclic and macrocyclic derivatives **7⋅Pt**, **7⋅Ru⋅PF_6_**, **10⋅Pt** and **10⋅Ru⋅PF_6_** are effective sensors for a range of halides and oxoanions. The near‐infra red emitting ruthenium congeners exhibited an increased binding strength compared to platinum due to the cationic charge and thus additional electrostatic interactions. Intramolecular hydrogen‐bonding between the dipyridylbenzene ligand and the amide carbonyls increases the preorganisation of both acyclic and macrocyclic metal derivatives resulting in no discernible macrocyclic effect. Interlocked analogues were also prepared, and preliminary luminescent chloride anion spectrometric titrations with **12⋅Ru⋅(PF_6_)_2_** demonstrate a marked increase in halide binding affinity due to the complementary chloride binding pocket of the [2]rotaxane. ^1^H NMR binding titrations indicate the interlocked dicationic receptor is capable of chloride recognition even in competitive 30 % aqueous mixtures.

## Graphical abstract

**A series of acyclic**, macrocyclic and mechanically interlocked luminescent anion sensors have been prepared by incorporation of the isophthalamide motif into dipyridylbenzene to obtain cyclometallated complexes of Pt^II^ and Ru^II^. Anion titrations with the ruthenium [2]rotaxane demonstrate a marked increase in chloride binding affinity and indicate the interlocked receptor is capable of chloride recognition even in competitive 30 % aqueous mixtures (see figure).

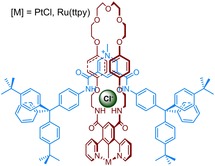

## Introduction

The 1,3‐di(2‐pyridyl)benzene ligand has been used to produce a diverse range of transition‐metal complexes with widespread utility in research areas such as catalysis,^1^ organic light emitting diodes (OLEDs)^2^ and energy transfer.^3^ Related to well‐known tridentate terpyridine transition‐metal complexes, they display a planar N^C^N coordination geometry, and transition‐metal complexes of dipyridylbenzene are typically extremely stable due to the synergistic combination of σ‐donating nitrogen and carbon atoms, and π‐accepting aromatic rings.

Complexes containing the dipyridylbenzene ligand have been reported for a range of late second and third row transition‐metals (M=Pt^II^, Ru^II^, Os^II^, Ir^III^, and Pd^II^).^4^ The strong field imparted by the ligand produces photo‐active complexes which typically emit in the visible and near‐infrared regions.^5^ In contrast to the ubiquitous terpyridine motif, the luminescence sensing properties of such systems are largely unexplored, and have been confined to volatile analytes.^6^ Anions are ubiquitous and their importance in biological systems and the environment, in particular from anthropogenic industrial activities, has over the past few decades stimulated the growth of abiotic anion receptor design, and importantly the need for the development of anion sensor materials. We^7^ and others^8^ have utilised the unique, three dimensional topological cavities of mechanically interlocked molecules (MIMs) for anion recognition applications. The integration of photo‐active reporter groups into MIM structural host frameworks enables such systems to selectively sense anions via optical luminescent methodologies.^9^ However, there is a paucity of such interlocked sensors incorporating luminophores which combine long lifetimes and efficient photosensitisation, such as cyclometallated transition‐metal complexes. Herein we incorporate an anion binding isophthalamide group into the dipyridylbenzene ligand structure to produce a range of acyclic, macrocyclic and rotaxane Pt^II^ and Ru^II^ transition‐metal‐based hosts and investigate their anion binding and sensing capabilities.

## Results and Discussion

### Synthesis

**Scheme 1:**
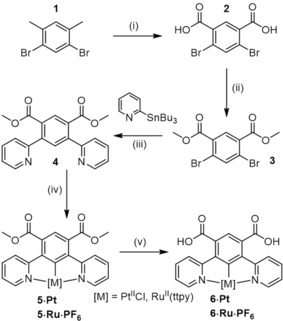
Synthesis of isophthalic acid functionalised complexes: (i) KMnO_4_, H_2_O/Pyridine, 89 %, (ii) MeOH/H_2_SO_4_, 89 %, (iii) PdCl_2_(PPh_3_)_2_, toluene, 58 %, (iv) M=Pt, K_2_PtCl_4_, 64 %; M=Ru, Ru(ttpy)Cl_3_, AgBF_4_, acetone, *n*‐butanol, 76 %, (v) M=Pt, KOH, MeOH, 99 %; M=Ru, NaOH, MeOH/1,4‐dioxane, 52 %.

The synthetic strategy devised involved the initial preparation of Pt^II^ and Ru^II^ cyclometallated complexes which contained carboxylic acid functionalities which would then facilitate integration into acyclic, macrocyclic and interlocked receptor structural frameworks via acid chloride‐amine condensation reactions, contrasting previous strategies of post‐synthetic modification of macrocyclic precursors or interlocked architectures.^10^ The synthesis of the dimethyl 4,6‐dipyridylisophthalate ligand **4** was accomplished using a 3‐step sequence (Scheme 1). Dibromo‐*m*‐xylene **1** 11 was oxidised using aqueous KMnO_4_ to afford **2** which was transformed to the methyl ester **3** via reaction in acidic methanol solution. The target ligand **4** was then obtained in 58 % yield by Pd^II^ catalysed Stille coupling between **3** and 2‐tributylstannyl pyridine and combined with the appropriate metal‐halide precursor (M=Pt, K_2_[PtCl_4_]; M=Ru, [Ru(4‐tolylterpyridine)Cl_3_]^12^) to obtain **5⋅Pt** and **5⋅Ru⋅PF_6_** cyclometallated esters. De‐esterification using basic conditions produced the target isophthalic acid functionalised synthons **6⋅Pt** and **6⋅Ru⋅PF_6_**.

**Scheme 2:**
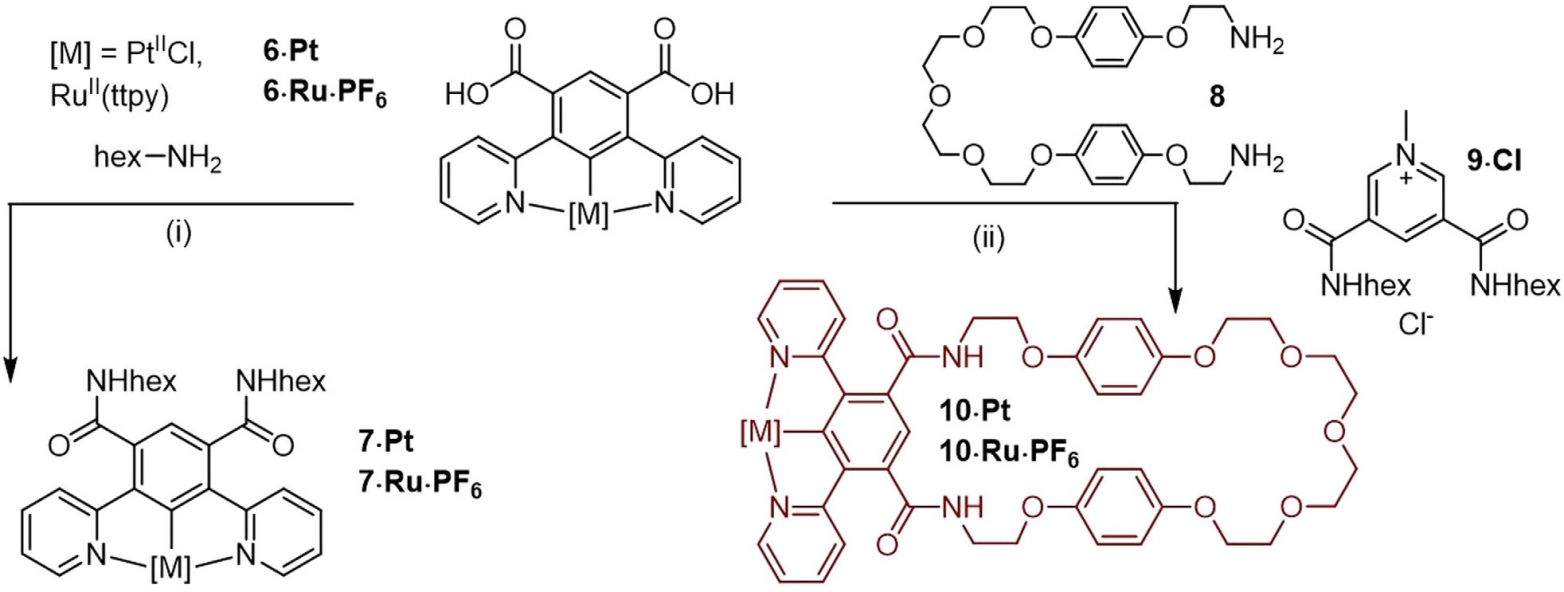
Synthesis of acyclic and macrocyclic Pt and Ru receptors: (i) (a) oxalyl chloride, CH_2_Cl_2_, (b) CH_2_Cl_2,_ Et_3_N, hexylamine, M=Pt, 6 %; M=Ru, (c) NH_4_PF_6(aq)_/CH_2_Cl_2,_ 81 %, (ii) (a) oxalyl chloride, CH_2_Cl_2_, (b) CH_2_Cl_2_, Et_3_N, 3,5‐bis‐hexylamide pyridinium chloride **9⋅Cl**, bisamine **8**, M=Pt, 24 %; M=Ru, (c) NH_4_PF_6(aq)_/CH_2_Cl_2_, 48 %.

The acyclic and macrocyclic isophthalamide receptors were prepared via acid chloride intermediates using oxalyl chloride before condensation with the appropriate amine (Scheme 2). In the case of the acyclic receptors *n*‐hexylamine was utilised to afford *n*‐hexylamide compounds, **7⋅Pt** and **7⋅Ru⋅PF_6_**. Macrocyclic targets were obtained via reaction with bis‐amine **8** 13 under high‐dilution conditions in the presence of an equimolar amount of 3,5‐bis‐hexylamide pyridinium chloride **9⋅Cl** 14 template to obtain **10⋅Pt** and **10⋅Ru⋅PF_6_** in 24 and 48 % respective yields.

**Scheme 3:**
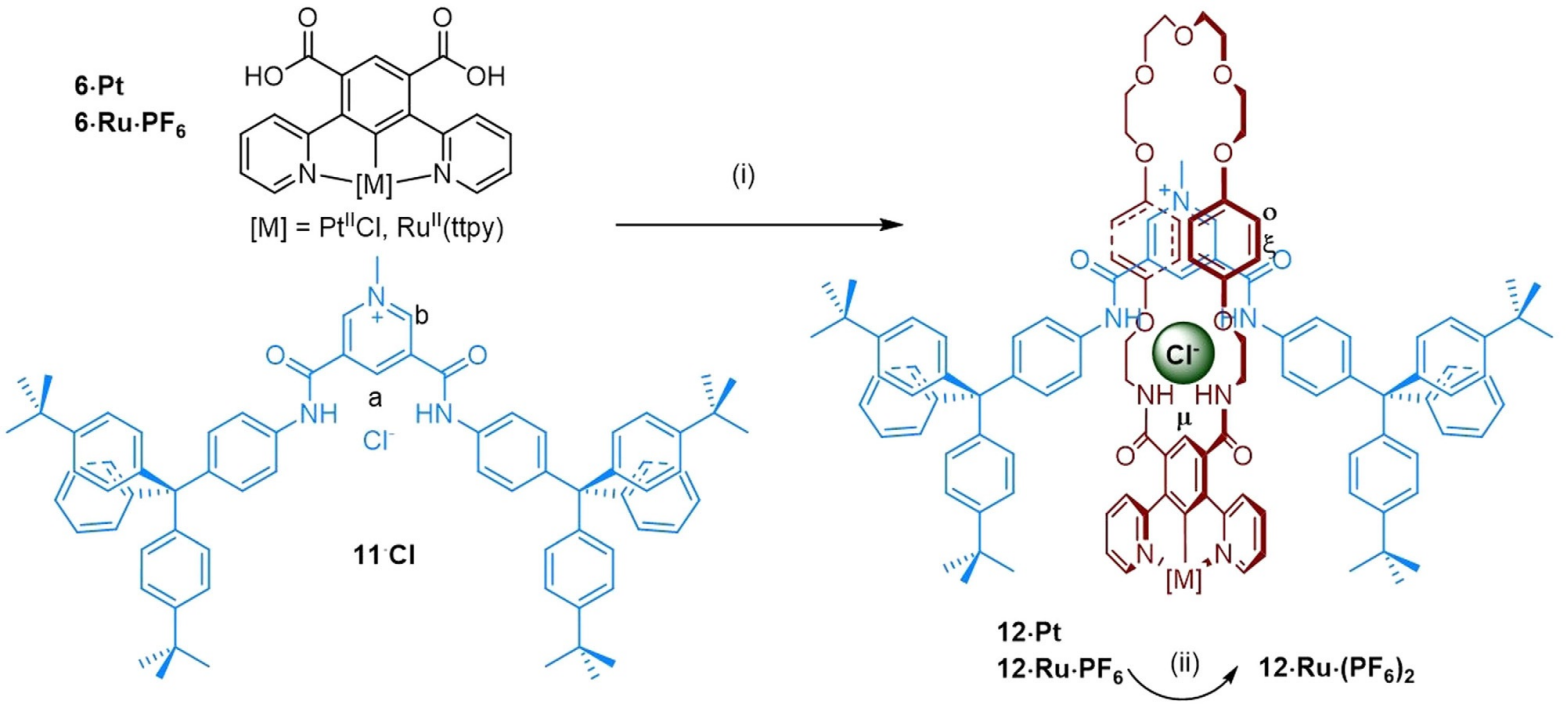
Synthesis of [2]rotaxane Pt and Ru receptors: (i) (a) oxalyl chloride, CH_2_Cl_2_, (b) CH_2_Cl_2_, Et_3_N, bisamine **8**, pyridinium axle **11⋅Cl**, M=Pt, 3 %; M=Ru, 8 %, (ii) NH_4_PF_6(aq)_/CH_2_Cl_2_, 81 %.

The target interlocked [2]rotaxane receptors were prepared using a chloride anion template clipping rotaxanation method,^13^, ^15^ utilising pyridinium chloride axle **11⋅Cl** 7c in place of the template **9⋅Cl** (Scheme 3). After challenging extensive size‐exclusion chromatography and preparative TLC purification, the target mechanically interlocked receptors **12⋅Pt⋅Cl** and **12⋅Ru⋅Cl_2_** were obtained in modest respective yields of 3 % and 8 %. Anion exchange of the mechanically interlocked ruthenium complex with aqueous NH_4_PF_6_ resulted in the formation of **12⋅Ru⋅(PF_6_)_2_**. All novel acyclic, macrocyclic and interlocked receptors were characterised by ^1^H NMR, ^13^C NMR and high‐resolution mass spectrometry (ESI‐HRMS or MALDI‐TOF‐MS), with the interlocked topology of the interlocked receptors determined by ^1^H‐^1^H ROESY NMR spectroscopy (See Electronic Supplementary Information).

### X‐ray crystallography

**Figure 1:**
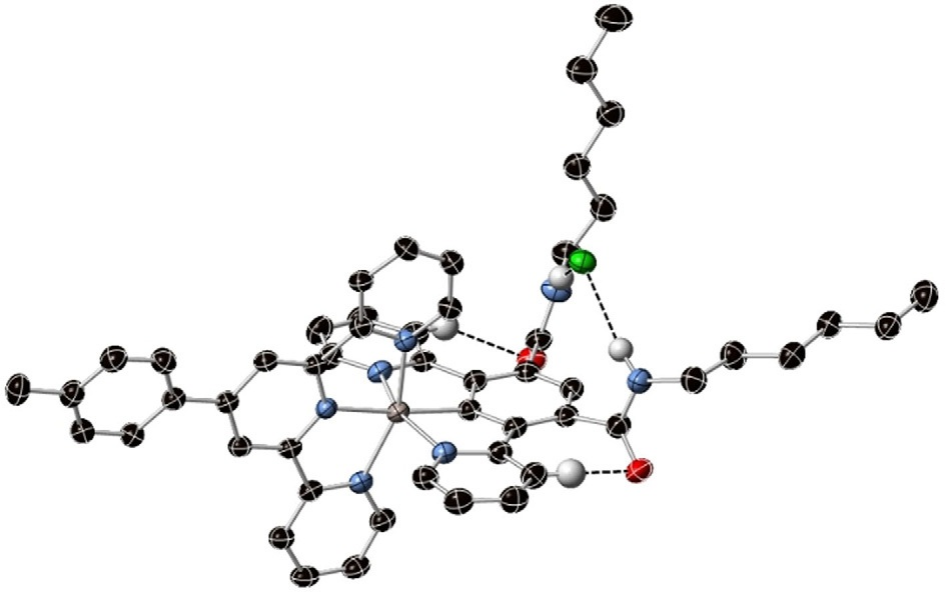
Single‐crystal X‐ray structure of *syn*‐**7⋅Ru⋅Cl** (ellipsoids are plotted at the 50 % probability level; non‐hydrogen‐bonding hydrogens are omitted for clarity).

Single‐crystal X‐ray structural analysis was performed on **7⋅Ru⋅PF_6_**, which was crystallised in the presence of excess tetra‐*n‐*butylammonium chloride. The crystal structure (Figure 1), reveals the chloride counteranion is bound within the isophthalamide cleft of the receptor which adopts a *syn*‐ geometry in the solid state, exhibiting mean N−Cl bond lengths of 3.41 Å. The chloride is bound out of the plane of the bis‐amide motif, satisfying electrostatic interactions between the anion and the positively charged complex, as well as the two‐hydrogen bonding interactions with the amide protons. Additionally, there are secondary hydrogen‐bonding interactions between the dipyridylbenzene ligand and the isophthalamide carbonyl oxygens with mean C−O distances of 3.21 Å, giving rise to an additional stabilising effect of the out‐of‐plane geometry (Figure 1).

**Figure 2:**
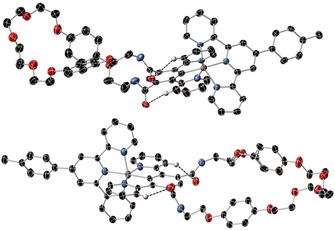
Single‐crystal X‐ray structure of (top) *syn*‐**10⋅Ru⋅PF_6_** and (bottom) anti‐**10⋅Ru⋅PF6** (ellipsoids are plotted at the 50 % probability level; counteranions, solvent and non‐hydrogen bonding hydrogen atoms omitted for clarity).

Crystals suitable for analysis by X‐ray diffraction were also obtained for the macrocyclic ruthenium receptor **10⋅Ru⋅PF_6_ i**n both the *syn*‐ and *anti*‐ geometry (Figure 2). Examination of the bonding in the solid state reveals that in both cases the supplementary hydrogen bonding interaction is present, despite the absence of a coordinating anion in the receptor cavity. The average C−O bond distance between the isophthalamide oxygens and the dipyridylbenzene ligand backbone are 3.20 and 3.11 Å for the *syn*‐ and *anti*‐ conformations, respectively.

### Photophysical characterisation

**Figure 3:**
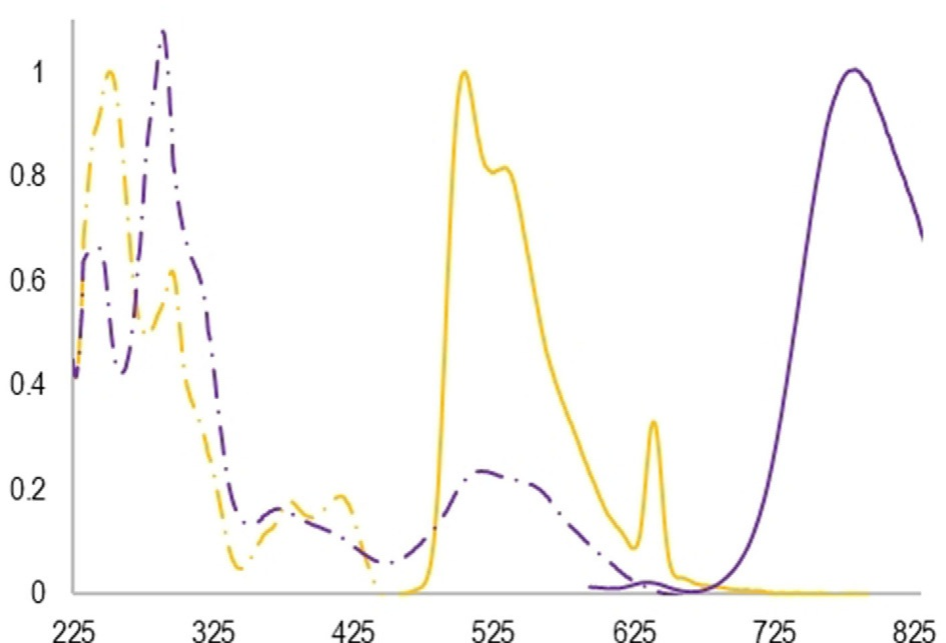
Absorption (dashed) and emission (solid lines) data for **7⋅Pt** (orange; *λ*_ex_=320 nm) and **7⋅Ru⋅PF_6_** (purple; *λ*_ex_=530 nm); intensity in arbitrary units.

The photophysical absorption and luminescent emissive properties of the novel receptors **7⋅Pt**, **7⋅Ru⋅PF_6_** (Figure 3), **10⋅Pt**, **10⋅Ru⋅PF_6_** and **12⋅Ru⋅(PF_6_)_2_** were investigated in aerated organic solvent media at 293 K (Table 1). The data shows that acyclic, macrocyclic and interlocked compounds generally retain the emissive properties of the corresponding parent unfunctionalised dipyridylbenzene complexes. The platinum receptors display a greater deviation of the emission to longer wavelength (Δ*λ*_em_=13 nm) in the isophthalamide functionalised receptors compared to previously reported dipyridylbenzene complexes. In the case of the ruthenium complexes this is significantly reduced (Δ*λ*_em_=2 nm), which can be rationalised by considering that the π* orbital—the MLCT acceptor—is located on the tolylterpyridine ligand rather than the dipyridylbenzene ligand, which is ancillary with respect to the emission.3c This contrasts the ^3^π–π emission of the platinum receptors which occur from the dipyridylbenzene moiety.

**Table 1:** Absorption and emission data for novel Pt and Ru receptors at 293 K; values are quoted in nm, extinction coefficients are displayed in parentheses.

Compound	Absorption [nm, *ϵ*/10^4^ m ^−1^ cm^−1^]	Emission [nm]
**7⋅Pt** ^[a]^	379 (0.86), 416 (0.90)	504
**10⋅Pt** ^[a]^	380 (0.90), 419 (1.00)	504
[Pt(dpyb)Cl]^[a]^	380 (0.87), 401 (0.70)	491
**7⋅Ru⋅PF_6_** ^[b]^	516 (1.42), 549 (1.28)	782
**10⋅Ru⋅PF_6_** ^[b]^	516 (1.28), 554 (1.17)	782
**12⋅Ru⋅(PF_6_)_2_** ^[b]^	516 (1.42), 557 (1.21)	782
[Ru(dpyb)(ttpy)] (PF_6_)_2_^[c]^	504 (1.08), 550 (0.83)	784

[a] CH_2_Cl_2_.4a [b] CH_2_Cl_2_/MeOH 95:5. [c] MeCN.^16^

### Luminescent anion binding titrations

**Figure 4:**
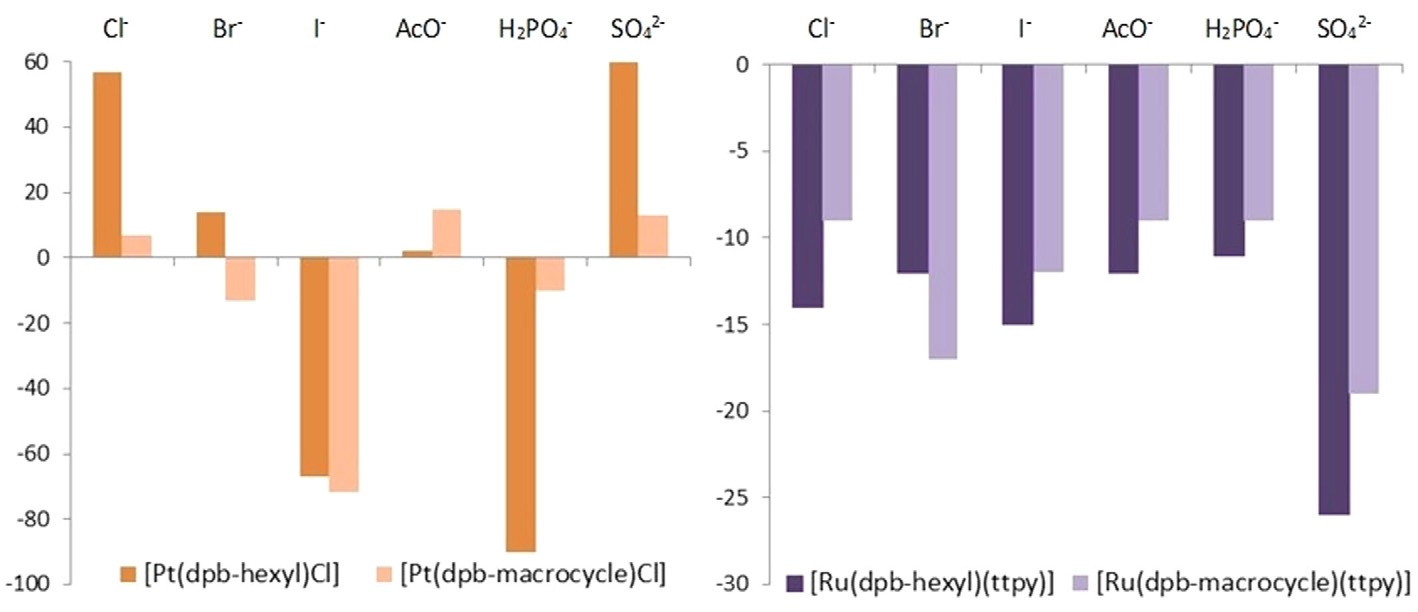
Percentage changes in luminescence emission intensity of (left) Pt receptors **7⋅Pt** and **10⋅Pt** (CH_2_Cl_2_, *λ*_em_=504 nm, *λ*_ex_=320 nm, 0–100 equivalents of anion) and (right) Ru receptors **7⋅Ru⋅PF_6_** and **10⋅Ru⋅PF_6_** (CH_2_Cl_2_, *λ*_em_=782 nm, *λ*_ex_=530 nm, 0–30 equivalents of anion) at 293 K.

Luminescent anion titrations of the acyclic and macrocyclic receptors were conducted for a range of halides and oxoanions (Figure 4). The acyclic platinum receptor **7⋅Pt** displayed the greatest change in emission across the chosen guests, exhibiting a ca. 60 % increase in emission in the presence of chloride and sulfate, and modest enhancement in the presence of bromide, which can be rationalised by rigidification of the receptor upon halide guest binding, thus disfavouring non‐radiative decay pathways. This was less prominent for the macrocyclic congener **10⋅Pt** which is clearly more conformationally restricted. In the case of iodide both receptors exhibited a significant (ca. 70 %) diminution of emission, which can be attributed to heavy atom quenching. The large decrease in emission intensity upon addition of dihydrogenphosphate ions can be tentatively attributed to photoinduced electron transfer which is common among basic oxoanions.^17^ Again, it is noteworthy that the macrocyclic analogue displayed much smaller changes upon addition of anionic substrates. For the acyclic and macrocyclic ruthenium receptors **7⋅Ru⋅PF_6_** and **10⋅Ru⋅PF_6_** quenching behaviour was exhibited for all anions. Furthermore, the percentage changes are much more modest compared with the platinum hosts, which is a consequence of the distal ^3^MLCT terpyridine moiety and dipyridylbenzene anion‐binding fragment.

Anion association constants for the acyclic and macrocyclic receptors were determined using global SPECFIT analysis of the titration data (Table 2).^18^ All systems displayed formation of 1:1 stoichiometric host‐guest complexes. The association constant values for **7⋅Pt** and **10⋅Pt** in CH_2_Cl_2_ show that all halides are bound with comparable strength, with the acyclic receptor exhibiting the highest affinity for the dihydrogenphosphate anion. Comparison between the two platinum hosts shows the magnitude of halide binding is similar in the acyclic and macrocyclic congeners. It is thought that supplementary secondary hydrogen‐bonding interactions evident in the solid‐state structures give rise to a highly preorganised isophthalamide motif. These intramolecular interactions increase the degree of preorganisation in the receptors to such an extent that little or no macrocyclic effect is observed. For both ruthenium receptors **7⋅Ru⋅PF_6_** and **10⋅Ru⋅PF_6_** the determined association constants for all anions studied were log*K*_a_>5 in CH_2_Cl_2_ and were repeated in more competitive media (CH_2_Cl_2_/MeOH 95:5). The stronger association for the ruthenium host systems is a result of the inherent positive charge and consequent additional electrostatic contributions between host and guest. In the more competitive solvent system both receptors display a modest preference for bromide amongst the halides. The sulfate anion has the strongest association, particularly for the acyclic ruthenium receptor **7⋅Ru⋅PF_6_**, which can be rationalised on electrostatic grounds. In analogy with the platinum systems, there is again little evidence for a discernible macrocyclic effect which may be attributed to the high degree of preorganisation of the acyclic receptor.

**Table 2:** Anion association constants determined by global‐fitting analysis; anions added as their tetra‐*n*‐butyl ammonium salts.

	**7⋅Pt** ^[a]^	**10⋅Pt** ^[a]^	**7⋅Ru⋅PF_6_** ^[b]^	**10⋅Ru⋅PF_6_** ^[b]^	**12⋅Ru⋅(PF_6_)_2_** ^[b]^
Cl^−^	3.43 (0.04)	3.43 (0.14)	3.89 (0.07)	3.65 (0.08)	4.61 (0.6), 2.84 (0.5)
Br^−^	3.83 (0.09)	3.13 (0.48)	4.16 (0.14)	4.09 (0.12)	–^[d]^
I^−^	3.39 (0.03)	3.90 (0.06)	3.90 (0.08)	3.77 (0.08)	–^[d]^
AcO^−^	^[c]^	2.27 (0.79)	3.25 (0.16)	3.34 (0.13)	–^[d]^
H_2_PO_4_^−^	4.12 (0.04)	2.57 (0.56)	3.34 (0.17)	3.41 (0.07)	–^[d]^
SO_4_^2−^	3.28 (0.04)	2.72 (0.54)	4.82 (0.14)	4.08 (0.06)	–^[d]^

[a] CH_2_Cl_2_. [b] CH_2_Cl_2_/MeOH 95:5 (v/v). [c] Data could not be fitted to a simple binding model. [d] Not performed.

An analogous titration with chloride was also performed for the interlocked ruthenium receptor **12⋅Ru⋅(PF_6_)_2_** and fitted to a 1:2 host‐guest stoichiometric binding model. The first anion binding event is presumed to occur within the interlocked [2]rotaxane cavity, resulting in an association constant which is greater by an order of magnitude than either the acyclic or macrocyclic ruthenium receptors. The second binding event is relatively weak and likely occurs via association, in a peripheral fashion, to the positively charged metal centre.

### Rotaxane ^1^H NMR anion binding titration

**Figure 5:**
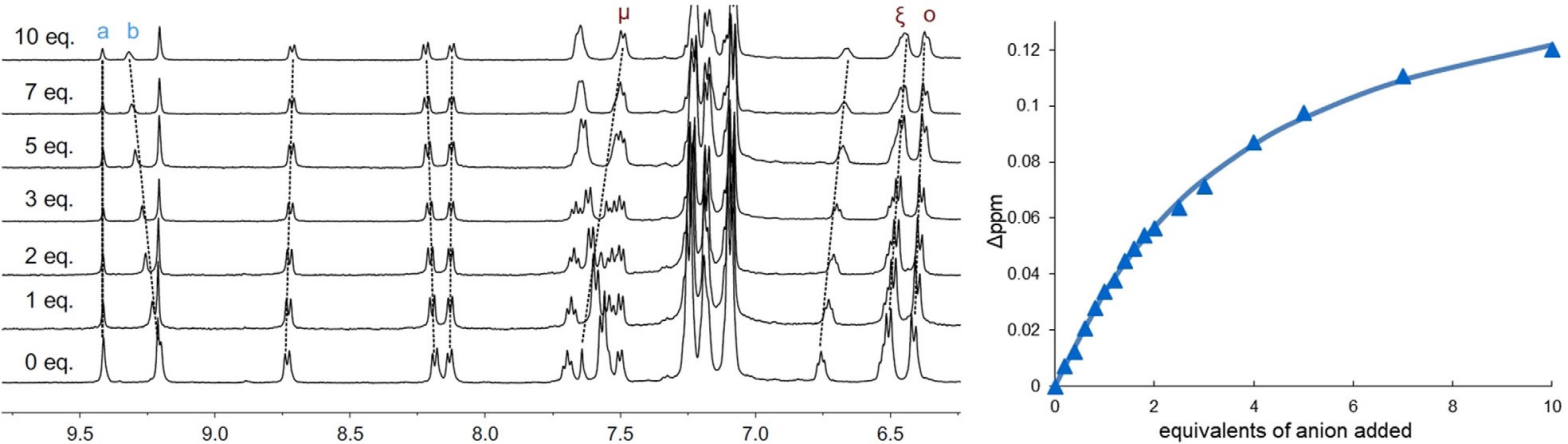
^1^H NMR (500 MHz, (CD_3_)_2_CO/D_2_O (7:3), 298 K) spectra of [2]rotaxane **12⋅Ru⋅(PF_6_)_2_** in the presence of increasing amounts of tetra‐*n‐*butylammonium chloride (left); anion binding curve of chemical shift change of proton *a*; symbols represent experimental data, continuous line represent calculated binding curve (right).

To elucidate the binding mode of chloride with the mechanically interlocked host **12⋅Ru⋅(PF_6_)_2_**, a ^1^H NMR anion binding titration was conducted in a competitive aqueous solvent mixture ((CD_3_)_2_CO/D_2_O 7:3) (Figure 5). The observed downfield shifts of the pyridinium axle protons *a* and *b* are indicative of the halide binding within the [2]rotaxane cavity. However, the shift of the internal macrocycle resonance *μ* are perturbed upfield which suggests little interaction of this proton with the chloride anion. Likewise, upfield shifts of the macrocycle hydroquinone protons ζ and ο are observed upon addition of chloride. This behaviour, in addition to the X‐ray single‐crystal analysis, suggests that chloride is bound only partially within the rotaxane cavity to maximise the electrostatic interactions with the cationic ruthenium complex, although time‐averaged *C*_2*v*_ symmetry persists throughout the titration. The titration data for chloride was analysed using WinEQNMR2 by monitoring the internal axle proton *a* to determine a 1:1 stoichiometric association constant of 196±13 m ^−1^,^19^ demonstrating **12⋅Ru⋅(PF_6_)_2_** rotaxane is capable of binding chloride in a highly competitive aqueous solvent system that contains 30 % water.

## Conclusions

Through the integration of the isophthalamide group into the dipyridylbenzene ligand structure, a series of novel acyclic, macrocyclic and interlocked rotaxane anion receptors incorporating luminescent cyclometallated platinum (II) and ruthenium (II) dipyridylbenzene complexes were prepared.

The acyclic and macrocyclic derivatives **7⋅Pt**, **7⋅Ru⋅PF_6_**, **10⋅Pt** and **10⋅Ru⋅PF_6_** were demonstrated to be effective luminescent sensors for a range of anions. In the case of the ruthenium receptors emission was observed in the near‐infrared region (*λ*_em_=782 nm), which is interesting from a biological standpoint. By virtue of additional favourable electrostatic interactions, the cationic ruthenium receptors displayed an enhanced affinity for anionic guests compared to the neutral platinum analogues. With both metal receptor systems, there was no discernible increase in anion binding affinity with the macrocyclic derivatives in comparison to the acyclic analogues which may be due to preorganising secondary hydrogen‐bonding interactions between the amide carbonyls and the dipyridylbenzene ligand backbone. Two mechanically interlocked rotaxane congeners were also synthesised. Preliminary luminescence spectroscopy anion binding titrations with rotaxane **12⋅Ru⋅(PF_6_)_2_** revealed a significant increase in chloride binding affinity compared to the non‐interlocked acyclic and macrocyclic receptors, owing to the complementary binding cavity of the interlocked host. ^1^H NMR titration experiments demonstrated **12⋅Ru⋅(PF_6_)_2_** to be capable of binding chloride even in competitive 30 % aqueous solvent mixtures.

## Experimental Section

### General considerations

Commercial grade chemicals and solvents were used without further purification. Where anhydrous solvents were used, they were degassed with N_2_ and passed through an MBraun MPSP‐800 column. Where degassed solvents were used, they were degassed via bubbling of N_2_ gas through the solution unless stated otherwise. Triethylamine was distilled from, and stored over potassium hydroxide. De‐ionised water dispensed from a Millipore Milli‐Q purification system was used in all cases. Tetrabutylammonium (TBA) salts were stored under vacuum in a desiccator. Microwave reactions were carried out using a Biotage Initiator 2.0 microwave. All synthetic procedures have been reliably repeated multiple times. Routine 300 MHz NMR spectra were recorded on a Varian Mercury 300 spectrometer, ^1^H NMR operating at 300 MHz, ^13^C{^1^H} at 76 MHz, ^19^F at 283 MHz and ^31^P at 121 MHz. Where the solubility of the compounds were too low, or not enough compound existed, a Bruker AVII500 with ^13^C Cryoprobe spectrometer was used for obtaining ^13^C{^1^H} at 126 MHz, however in some cases a complete ^13^C{^1^H} spectrum could not be obtained. All 500 MHz ^1^H Spectra and all ^1^H NMR titrations were recorded on a Varian Unity Plus 500 spectrometer. All chemical shift (*δ*) values are given in parts per million and are referenced to the solvent. In cases where solvent mixtures are used, the main solvent is used as the reference. Where an apparent multiplet (e.g. app. t.) is quoted, *J*_app_ is given. Low resolution ESI mass spectra were recorded on a Micromass LCT Premier XE spectrometer. Accurate masses were determined to four decimal places using Bruker μTOF and Micromass GCT spectrometers. UV/visible experiments were carried out on a PG instruments T60U spectrometer at 293 K. Steady‐state fluorescence spectra were recorded on Jobin–Yvon–Horiba Fluorolog‐3 spectrophotometer or a Varian Cary‐Eclipse spectrometer. 2,4‐dibromo‐1,5‐dimethylbenzene **1**,^11^ 2‐tributylstannylpyridine,^20^ 4‐tolyl‐2,2′:6′,2′′‐terpyridine,^21^ 4‐tolyl‐2,2′:6′,2′′‐terpyridine ruthenium trichloride,^12^ diamine **8**,10b 3,5‐bis‐hexylamide pyridinium chloride **9⋅Cl** 14 and pyridinium axle⋅Cl **11⋅Cl**,7c were synthesised as previously described.

### Synthesis of (dihexyl‐2,4‐di‐2‐pyridyl‐isophthalamide) platinum(II) chloride ([Pt^II^(dpb‐hexyl)Cl]) 7⋅Pt

Oxalyl chloride (0.110 mL, 1.37 mmol) was added dropwise to a suspension of Pt^II^(dpb‐acid)Cl **6⋅Pt** (0.189 g, 0.343 mmol, ) in anhydrous CH_2_Cl_2_ (100 mL) and anhydrous degassed catalytic DMF (1 drop). The reaction mixture was stirred under N_2_ at ambient temperature until homogenous (ca. 6 hours). The solvent was removed in vacuo and dried under high vacuum. Anhydrous, distilled Et_3_N (0.384 mL, 2.75 mmol) and hexylamine (0.270 mL, 2.06 mmol) were added to the acid chloride intermediate dissolved in CH_2_Cl_2_ (100 mL) at 0 °C. The reaction mixture was stirred for two hours at ambient temperature. The resulting solution was washed with sat. NaHCO_3(aq)_ (50 mL), 10 % w/w citric acid (50 mL) and sat. brine (50 mL) and dried over anhydrous MgSO_4_. The solvent was removed in vacuo. Purification by preparative thin‐layer chromatography (SiO_2;_ CH_2_Cl_2_/MeOH; 90:10) gave a yellow solid (15.2 mg, 6 %, 22 μmol). ^1^H NMR (500 MHz, CDCl_3_): *δ*_H_=9.55 (2 H, d, ^4^ *J*_HH_=5.8, ArH), 9.13 (2 H, t, ^3^ *J*_HH_=5.6, NH), 8.47 (2 H, app. t, *J*_app_=8.0, ArH), 8.10 (2 H, d, ^3^ *J*_HH_=8.3, ArH), 7.87 (2 H, app. t, *J*_app_=6.6, ArH), 1.45–1.56 (4 H, m, NHCH_2_), 1.20–1.38 (16 H, m, CH_2_), 0.69–0.86 ppm (6 H, m, CH_3_); ^13^C NMR (76 MHz, CDCl_3_): *δ*_C_=168.6, 164.3, 151.3, 137.7, 136.8, 134.2, 123.7, 122.4, 122.0, 40.1, 31.7, 29.6, 27.0, 22.8, 14.3 ppm; EI‐HRMS *m*/*z* calcd for [C_30_H_37_ClN_4_O_4_Pt−Cl]^+^ 680.2564, found 680.2791; UV/Vis *λ*_max_(CH_2_Cl_2_) nm^−1^; *ϵ*/dm^3^ mol^−1^ cm^−1^: 251 (48,100) 295 (29,700) 364 (5,800) 379 (8,600) 416 (9,000).

### Synthesis of (dihexyl 2,4‐di‐2‐pyridyl‐isophthalamide)(4‐tolylterpyridine) ruthenium(II) hexafluorophosphate ([Ru^II^(dpb‐hexyl)(ttpy)PF6]) 7⋅Ru⋅PF_6_

Oxalyl chloride (0.00400 mL, 0.0520 mmol) was added dropwise to a solution of [Ru^II^(dpb‐acid)(ttpy)PF_6_] 6**⋅**Ru**⋅**PF_6_ (9.6 mg, 0.0130 mmol) in anhydrous CH_2_Cl_2_ (50 mL) and anhydrous degassed catalytic DMF (1 drop). The reaction mixture was stirred under N_2_ at ambient temperature until homogenous. The solvent was removed in vacuo and dried under high vacuum. Anhydrous, distilled Et_3_N (0.0140 mL, 0.104 mmol) and hexylamine (0.0100 mL, 0.0780 mmol) were added to the acid chloride intermediate dissolved in anhydrous CH_2_Cl_2_ (50 mL) at 0 °C. The reaction mixture was stirred for two hours at ambient temperature under N_2_. The solvent was then removed in vacuo and the solid was dissolved in CH_2_Cl_2_ (15 mL) and washed with 0.1 m NH_4_PF_6(aq)_ (10×10 mL) and H_2_O (2×10 mL). The organic layer was dried over MgSO_4_, and the solvent removed in vacuo. Purification by preparative thin‐layer chromatography (SiO_2_; CH_2_Cl_2_/MeOH; 90:10) gave a purple solid (9.24 mg, 81 %). ^1^H NMR (500 MHz, CD_2_Cl_2_): *δ*_H_=8.77 (2 H, s, ArH), 8.35 (2 H, app. dt, *J*_app_=8.1, 1.2, ArH), 8.28 (2 H, app. dd, *J*_app_=8.4, 1.1, ArH), 8.01–7.93 (2 H, m, ArH), 7.65 (2 H, app. td, *J*_app_=7.8, 1.5, ArH), 7.58–7.47 (4 H, m, ArH), 7.29 (1 H, s, ArH), 7.28–7.23 (2 H, m, ArH), 7.09–7.05 (2 H, m, ArH), 6.99 (2 H, app. ddd, *J*_app_=7.3, 5.6, 1.4, ArH), 6.83 (2 H, t, ^3^ *J*_HH_=5.8, ArH), 6.63 (2 H, app. ddd, *J*_app_=7.2, 5.7, 1.4, ArH), 3.58 (4 H, app. td, *J*_app_=7.3, 5.8, NCH_2_), 2.54 (3 H, s, CH_3_), 1.77–1.68 (4 H, m, CH_2_), 1.52–1.43 (4 H, m, CH_2_), 1.33–1.39 (8 H, m CH_2_), 0.92–0.88 ppm (6 H, m, CH_3_); ^13^C NMR (126 MHz, CDCl_3_): *δ*_C_=171.6, 167.6, 157.9, 156.6, 153.1, 150.2, 135.2, 134.5, 130.7, 127.2, 123.6, 122.5, 121.6, 119.2, 53.6, 46.0, 40.5, 31.7, 29.9, 29.4, 26.9, 22.8, 21.6, 15.4, 14.2 ppm, remaining quaternary carbons undetected; ^19^F NMR (283 MHz, CDCl_3_): *δ*_F_=−72.5 ppm (d, ^1^ *J*=713 Hz, P*F*_6_); ^31^P NMR (121 MHz, CDCl_3_): *δ*_P_=−144.53 ppm (sept., ^1^ *J*=713 Hz, *P*F_6_); ESI‐HRMS *m*/*z* calcd for [C_52_H_54_F_6_N_7_O_2_PRu−PF_6_]^+^ 910.3391, found 910.3390; UV/Vis *λ*_max_(CH_2_Cl_2_) nm^−1^; *ϵ*/dm^3^ mol^−1^ cm^−1^: 241 (39,900) 289 (64,800) 374 (9,600) 616 (14,200) 549 (12,800)

### Synthesis of (2,4‐di‐2‐pyridyl‐isophthalamide macrocycle) platinum(II) chloride ([Pt^II^(dpb‐macrocycle)Cl]) 10⋅Pt

Oxalyl chloride (11 μL, 0.138 mmol) was added dropwise to a solution of Pt^II^(dpbacid)Cl **6⋅Pt** (19.0 mg, 0.0346 mmol) in anhydrous CH_2_Cl_2_ (100 mL) and anhydrous degassed catalytic DMF (1 drop). The reaction mixture was stirred under N_2_ at ambient temperature until homogenous (ca. 6 hours). The solvent was removed in vacuo and dried under high vacuum. Bis‐amine (0.0160 g, 0.0346 mmol), pyridinium thread **9⋅**Cl (0.0130 g, 0.0346 mmol) and anhydrous, distilled Et_3_N (0.0120 mL, 0.0865 mmol) were dissolved in anhydrous CH_2_Cl_2_ (100 mL) and stirred until homogenous. The acid chloride in anhydrous CH_2_Cl_2_ (100 mL) was added dropwise, and the reaction mixture stirred for three hours at ambient temperature under N_2_. The solvent was removed in vacuo and the resulting solid was purified by preparative thin‐layer chromatography (SiO_2_; CH_2_Cl_2_/MeOH; 98:2) to give a yellow solid (8.1 mg, 24 %, 8.2 μmol). ^1^H NMR (500 MHz, [D_6_]DMSO): *δ*_H_=9.35 (2 H, d, ^3^ *J*_HH_=5.6, ArH), 9.17 (2 H, t, ^3^ *J*_HH_=5.6 Hz, NH), 8.28 (2 H, app. t, *J*_app_=7.6, ArH), 7.95 (2 H, d, ^3^ *J*_HH_=7.4, ArH), 7.67 (2 H, app. t, *J*_app_=7.5, ArH), 7.12 (1 H, s, ArH), 6.91 (4 H, d, ^3^ *J*_HH_=9.0, ArH), 6.84 (4 H, d, ^3^ *J*_HH_=9.0, ArH), 4.11 (4 H, t, ^3^ *J*_HH_=5.4, CH_2_), 3.97 (4 H, t, ^3^ *J*_HH_=4.8, CH_2_), 3.65–3.75 (8 H, m, CH_2_), 3.48–3.59 ppm (8 H, m, CH_2_); ^13^C NMR (126 MHz, [D_6_]DMSO): *δ*_C_=167.9, 164.4, 152.8, 152.4, 140.6, 136.7, 135.3, 124.9, 123.1, 122.5, 115.6, 115.3, 70.0, 69.0, 69.0, 67.7, 66.5, 30.7 ppm; MALDI‐TOF MS *m*/*z* calcd for [C_42_H_43_ClN_4_O_9_Pt−Cl]^+^ 942.27, found 942.02; UV/Vis *λ*_max_(CH_2_Cl_2_) nm^−1^; *ϵ*/dm^3^ mol^−1^ cm^−1^: 234 (69,000) 253 (60,600), 295 (49,600) 361 (8,400) 380 (9,000) 419 (10,000).

### Synthesis of (2,4‐di‐2‐pyridyl‐isophthalamide macrocycle)(4‐tolylterpyridine) ruthenium(II) hexafluorophosphate ([Ru^II^(dpb‐macrocycle)(ttpy)PF_6_]) 10⋅Ru⋅PF_6_

Oxalyl chloride (0.0400 mL, 0.450 mmol) was added dropwise to a solution of [Ru^II^(dpb‐acid)(ttpy)PF_6_] 6**⋅**Ru**⋅**PF_6_ (0.100 g, 0.112 mmol) in anhydrous CH_2_Cl_2_ (100 mL) and anhydrous degassed catalytic DMF (1 drop). The reaction mixture was stirred under N_2_ at ambient temperature until homogenous (ca. 6 hours). The solvent was removed in vacuo and dried under high vacuum. Bis‐amine 8 (0.0521 g, 0.112 mmol), pyridinium thread 9**⋅**Cl (0.0430 g, 0.112 mmol) and anhydrous, distilled Et_3_N (0.0400 mL, 0.281 mmol) were dissolved in anhydrous CH_2_Cl_2_ (100 mL) and stirred until homogenous. The acid chloride, in anhydrous CH_2_Cl_2_ (100 mL), was added dropwise, and the reaction mixture stirred for three hours at ambient temperature under N_2_. The solvent was removed in vacuo. The resulting solid was dissolved in CH_2_Cl_2_ (15 mL) and washed with 0.1 m NH_4_PF_6(aq)_ (10×10 mL) and H_2_O (2×10 mL). The organic layer was dried over MgSO_4_, and the solvent removed in vacuo. The resulting solid was purified by column chromatography (SiO_2_; CH_2_Cl_2_/MeOH; 96:4) and preparative thin‐layer chromatography (SiO_2_; CH_2_Cl_2_/MeOH; 98:2) to give a purple solid (71.5 mg, 48 %, 54.2 μmol). ^1^H NMR (500 MHz, CDCl_3_): *δ*_H_=9.47 (2 H, t, ^3^ *J*_HH_=5.6, ArH), 8.76 (2 H, s, ArH), 8.55 (2 H, d, ^3^ *J*_HH_=8.5, ArH), 8.35 (2 H, d, ^3^ *J*_HH_=8.1, ArH), 7.97 (2 H, d, ^3^ *J*_HH_=7.8, ArH), 7.59 (1 H, s, ArH), 7.55 (2 H, t, ^3^ *J*_HH_=7.9, ArH), 7.50 (2 H, d, ^3^ *J*_HH_=7.9, ArH), 7.44 (2 H, d, ^3^ *J*_HH_=5.5, ArH), 7.37 (2 H, t, ^3^ *J*_HH_=8.0, ArH), 7.00 (2 H, t, ^3^ *J*_HH_=6.7, ArH), 6.91–6.84 (6 H, m, ArH), 6.62 (4 H, d, ^3^ *J*_HH_=8.6, ArH), 6.50 (2 H, t, ^3^ *J*_HH_=6.6, ArH), 4.27 (4 H, t, ^3^ *J*_HH_=6.1, CH_2_), 3.94 (4 H, app. q, *J*_app_=6.0, CH_2_), 3.84 (4 H, t, ^3^ *J*_HH_=5.0, CH_2_), 3.77 (4 H, t, ^3^ *J*_HH_=4.9, CH_2_), 3.71 (8H s, CH_2_), 2.50 ppm (3 H, s, CH_3_); ^13^C NMR (126 MHz, CDCl_3_): *δ*_c_=165.5, 158.3, 153.9, 151.9, 148.6, 147.0, 145.5, 145.0, 143.6, 136.7, 135.2, 134.9, 133.9, 132.0, 131.2, 130.8, 127.5, 126.0, 124.5, 120.6, 116.3, 115.0, 114.9, 70.9, 70.7, 70.2, 68.5, 65.9, 64.0, 34.5, 31.6 ppm; ^19^F NMR (283 MHz, CDCl_3_): *δ*_F_=−72.0 (d, ^1^ *J*=713 Hz, P*F*_6_); ^31^P NMR (121 MHz, CDCl_3_): *δ*_P_=−144.3 ppm (sept., ^1^ *J*_PF_=714 Hz, PF_6_); ESI‐HRMS *m*/*z* calcd for [C_64_H_60_F_6_N_7_O_9_PRu−PF_6_]^+^ 1172.3508, found 1172.3528; UV/Vis *λ*_max_(CH_2_Cl_2_) nm^−1^; *ϵ*/dm^3^ mol^−1^ cm^−1^: 233 (40,400) 288 (64,300) 373 (9,300) 516 (12,900) 554 (11,700)

### Synthesis of [2]rotaxane (2,4‐di‐2‐pyridyl‐isophthalamide macrocycle) platinum(II) chloride ([Pt^II^(dpb‐macrocycle)Cl] [2]rotaxane) 12⋅Pt⋅Cl

Oxalyl chloride (0.0400 mL, 0.496 mmol) was added dropwise to a solution of Pt^II^(dpb‐acid)Cl 6**⋅**Pt (0.0682 g, 0.124 mmol) in anhydrous CH_2_Cl_2_ (100 mL) and anhydrous degassed catalytic DMF (1 drop). The reaction mixture was stirred under N_2_ at 40 °C until homogenous (ca. 6 hours). The solvent was removed in vacuo and dried under high vacuum. Bis‐amine 8 (0.0576 g, 0.124 mmol), pyridinium axle 11**⋅**Cl (0.133 g, 0.124 mmol) and anhydrous, distilled Et_3_N (0.043 mL, 0.310 mmol) were dissolved in anhydrous CH_2_Cl_2_ (100 mL) and stirred until homogenous. The acid chloride in anhydrous CH_2_Cl_2_ (100 mL) was added dropwise, and the reaction mixture stirred for three hours at ambient temperature under N_2_. The solvent was removed in vacuo. Purification by column chromatography (SiO_2_; CH_2_Cl_2_/MeOH 99:1→95:5), preparative thin‐layer chromatography (SiO_2_; CH_2_Cl_2_/MeOH 99:1→95:5) and size‐exclusion chromatography (Bio‐Beads S‐X1/ CHCl_3_) gave the compound as a yellow solid (7.6 mg, 3 %, 3.7 μmol). ^1^H NMR (500 MHz, CDCl_3_/CD_3_OD 9:1) 9.58 (1 H, s, PyH), 9.32 (2 H, d, ^3^ *J*_HH_=5.8, ArH), 9.13 (1 H, s, ArH), 8.75 (2 H, s, PyH), 8.04 (1 H, s, ArH), 7.79 (2 H, d, ^3^ *J*_HH_=8.3, ArH), 7.67 (6 H, app. dd, *J*_app_=20.6, 8.3, ArH + StH), 7.51–7.46 (1 H, m, ArH), 7.39 (1 H, s, ArH), 7.33–7.07 (20 H, m, ArH + StH), 7.04 (8 H, d, ^3^ *J*_HH_=8.5, StH), 6.64 (4 H, d, ^3^ *J*_HH_=8.5, HQH), 6.28 (4 H, d, ^3^ *J*_HH_=8.6, HQH), 4.09 (4 H, s, OCH_2_), 3.92 (4 H, s, OCH_2_), 3.75 (4 H, s, OCH_2_), 3.65–3.56 (8 H, m, OCH_2_), 3.48–3.42 (4 H, m, NCH_2_), 3.30 (3 H, s, NCH_3_), 1.22 (36 H, s, C(CH_3_)_3_); ^13^C NMR (126 MHz, CDCl_3_/CD_3_OD 9:1): *δ*_c_=169.1, 164.7, 158.7, 152.9, 151.9, 148.7, 146.9, 144.8, 143.5, 139.3, 134.9, 133.9, 133.6, 131.9, 131.0, 130.6, 128.8, 127.4, 125.9, 124.3, 123.6, 123.1, 119.6, 115.5, 115.0, 70.7, 70.6, 70.2, 68.3, 67.5, 66.2, 63.9, 57.8, 40.2, 38.7, 34.3, 31.3, 30.4, 29.7, 28.9, 23.7, 23.0, 22.7, 17.9, 14.0, 10.9 ppm; MALDI‐TOF MS *m*/*z* calcd for [C_116_H_121_Cl_2_N_7_O_11_Pt−Cl]^+^ 2018.32, found 2018.89.

### Synthesis of [2]rotaxane (2,4‐di‐2‐pyridyl‐isophthalamide)(4‐tolylterpyridine) ruthenium(II) hexafluorophosphate ([Ru^II^(dpb‐macrocycle)(ttpy)PF_6_] [2]rotaxane) 12⋅Ru⋅(PF_6_)_2_

Oxalyl chloride (0.08 mL, 0.1 mmol) was added dropwise to a solution of [Ru^II^(dpb‐acid)(ttpy)PF_6_] 6**⋅**Ru**⋅**PF_6_ (0.023 g, 0.025 mmol) in anhydrous CH_2_Cl_2_ (10 mL) and anhydrous degassed catalytic DMF (1 drop). The reaction mixture was stirred under N_2_ at 40 °C until homogenous (ca. 6 hours). The solvent was removed in vacuo and dried under high vacuum. Bis‐amine 8 (0.012 g, 0.025 mmol), pyridinium axle 11**⋅**Cl (0.027 g, 0.025 mmol) and anhydrous, distilled Et_3_N (0.008 mL, 0.0625 mmol) were dissolved in anhydrous CH_2_Cl_2_ (10 mL) and stirred until homogenous. The acid chloride in anhydrous CH_2_Cl_2_ (10 mL) was added dropwise, and the reaction mixture stirred for three hours at ambient temperature under N_2_. The solvent was removed in vacuo. Purification by column chromatography (SiO_2_; CH_2_Cl_2_/MeOH; 95:5) and by preparative thin‐layer chromatography (SiO_2_; EtOAc/MeOH; 100:0→98:2). This was dissolved in CH_2_Cl_2_ (15 mL) and washed with 0.1 m NH_4_PF_6(aq)_ (10×10 mL) and H_2_O (2×10 mL). The organic layer was dried over MgSO_4_, and the solvent removed in vacuo to give the compound as a purple solid (4.9 mg, 6.5 % over two‐steps, 1.95 μmol); ^1^H NMR (500 MHz, CD_2_Cl_2_): *δ*_H_=9.47 (1 H, br.s, ArH), 9.37 (1 H, br.s, ArH),8.78 (2 H, s, ArH), 8.67 (1 H, s, ArH + StH), 8.32 (4 H, d, ^3^ *J*_HH_=8.2, ArH±StH), 8.00 (4 H, d, ^3^ *J*_HH_=7.9, ArH + StH), 7.79 (4 H, s, ArH), 7.55 (5 H, app. dd, *J*_app_=15.0, 8.2, ArH), 7.38 (4 H, d, ^3^ *J*_HH_=8.5, StH), 7.30 (11 H, d, ^3^ *J*_HH_=7.5, ArH + StH), 7.23–7.20 (9 H, m, ArH + StH), 7.06 (4 H, d, ^3^ *J*_HH_=5.9, StH), 6.79 (4 H, d, ^3^ *J*_HH_=8.0, HQH), 6.62 (2 H, t, ^3^ *J*_HH_=6.6, ArH), 6.33 (4 H, d, ^3^ *J*_HH_=8.6, HQH), 4.10 (4 H, s, OCH_2_), 3.93 (4 H, s, OCH_2_), 3.77 (4 H, s, OCH_2_), 3.70 (4 H, s, OCH_2_), 3.63 (4 H, s, OCH_2_), 3.44 (4 H, s, NCH_2_), 2.53 (3 H, s, ArCH_3_), 1.24 ppm (36 H, s, C(CH_3_)_3_); ^13^C NMR (126 MHz, CDCl_3_/CD_3_OD 9:1): *δ*_c_=165.2, 165.1, 164.5, 164.4, 158.4, 158.3, 153.2, 152.2, 152.1, 148.7, 147.0, 146.0, 144.6, 144.5, 143.6, 143.5, 138.0, 136.6, 135.5, 134.8, 133.4, 131.8, 131.0, 130.6, 129.3, 128.9, 127.4, 127.3, 127.0, 125.8, 125.8, 124.4, 124.3, 123.8, 121.6, 120.6, 120.4, 114.9, 70.7, 70.6, 70.0, 68.3, 65.5, 63.8, 41.1, 41.0, 39.2, 34.3, 31.3, 29.7 ppm; ^19^F NMR (472 MHz, CD_2_Cl_2_): *δ*_F_=−70.9 (d, ^1^ *J*=742 Hz, PF_6_); ^31^P NMR (121 MHz, CDCl_3_) δ_P_=−144.35 ppm (sept., ^1^ *J*_PF_=714 Hz, PF_6_); ESI‐HRMS *m*/*z* calcd for [C_138_H_138_F_6_N_10_O_11_PRu−2PF_6_]^2+^ 1106.4793, found 1106.4795; UV/Vis *λ*_max_(CH_2_Cl_2_) nm^−1^; *ϵ*/dm^3^ mol^−1^ cm^−1^: 251 (96,300) 289 (64,000) 371 (9,800) 516 (14,200) 557 (12,100).

### Luminescent anion binding titrations

Luminescence experiments were carried out on a Varian Cary‐Eclipse spectrometer for the platinum (II) receptors using an excitation wavelength of 320 nm at 293 K. To a 2.5 mL, 1×10^−5^ m solution of each receptor was added aliquots of the tetrabutylammonium salts dissolved in a stock solution made up with the receptor, such that the same concentration of the host was maintained throughout the titration experiments. Luminescence experiments were carried out on a Horiba Fluorolog spectrometer for the ruthenium(II) receptors using an excitation wavelength of 530 nm at 293 K. To a 1 mL, 1×10^−4^ m solution of each receptor was added aliquots of the tetrabutylammonium salts dissolved in a stock solution made up with the receptor, such that the same concentration of the host was maintained throughout the titration experiments. In both cases the titration data was analysed and association constants determined using the SPECFIT program.^18^

### ^1^H NMR anion titration data

Initial NMR sample volumes and concentrations were 500 μL and 2.0 mm respectively. Solutions (100 mm) of anion were added as their tetrabutylammonium salts. Spectra were recorded at 0, 0.2, 0.4, 0.6, 0.8, 1.0, 1.2, 1.4, 1.6, 1.8, 2.0, 2.5, 3.0, 4.0, 5.0, 7.0 and 10.0 equivalents. In all cases where association constants were calculated, bound and unbound species were found to be in fast exchange on the NMR timescale. Association constants were obtained by analysis of the resulting data using the WinEQNMR2 computer program.^19^ Binding stoichiometry was investigated by visual analysis of the titration data, and using approximations of Job plots. Estimates for the association constant and the limiting chemical shifts were added to the program's input file. The parameters were refined by non‐linear least‐squares analysis using WINEQNMR2 to achieve the best fit between observed and calculated chemical shifts. The input parameters for the final chemical shift and association constant were adjusted based on the program output until convergence was reached. Comparison of the calculated and experimental binding isotherms demonstrated that an appropriate model with an appropriate stoichiometry were used.

## Conflict of interest

The authors declare no conflict of interest.

## Supporting information

As a service to our authors and readers, this journal provides supporting information supplied by the authors. Such materials are peer reviewed and may be re‐organized for online delivery, but are not copy‐edited or typeset. Technical support issues arising from supporting information (other than missing files) should be addressed to the authors.

Supplementary
