# Supplementary material for: Luminescent Anion Sensing by Transition‐Metal Dipyridylbenzene Complexes Incorporated into Acyclic, Macrocyclic and Interlocked Hosts
Source: Chemistry. 2020 Apr 1;26(23):5288–96. doi: 10.1002/chem.202000661 (PMC7216984; doi:10.1002/chem.202000661)
Supplement: Supplementary file 1 — Supplementary [file CHEM-26-5288-s001.pdf]

Chemistry–A European Journal

Supporting Information

Luminescent Anion Sensing by Transition-Metal Dipyridylbenzene Complexes Incorporated into Acyclic, Macrocyclic and Interlocked Hosts

Richard C. Knighton,* Sophie Dapin, and Paul D. Beer*[a]

Contents

1. Experimental and ^{1}H, ^{13}C NMR and HRMS spectra **2**

General Considerations *2*

Synthesis of 2,4-dibromo-isophthalic acid 2 .... *2*

Synthesis of dimethyl-2,4-dibromo-isophthalate ester 3 .... *3*

Synthesis of dimethyl-2,4-di-2-pyridyl-isophthalate ester (dpb-ester) 4 .... *4*

Synthesis of (dimethyl-2,4-di-2-pyridyl-isophthalate ester) platinum(II) chloride ([Pt^{II}(dpbester)Cl]) 5·Pt *6*

Synthesis of (dimethyl-2,4-di-2-pyridyl-isophthalate ester)(4-tolylterpyridine) ruthenium(II) hexafluorophosphate ([Ru^{II}(dpb-ester)(ttpy)PF$_6$]) 5·Ru·PF$_6$ *7*

Synthesis of (2,4-di-2-pyridyl-isophthalic acid) platinum(II) chloride ([Pt^{II}(dpbacid)Cl]) 6·Pt *8*

Synthesis of (2,4-di-2-pyridyl-isophthic acid)(4-tolylterpyridine) ruthenium(II) hexafluorophosphate ([Ru^{II}(dpb-acid)(ttpy)PF$_6$]) 6·Ru·PF$_6$ *9*

Synthesis of (dihexyl-2,4-di-2-pyridyl-isophthalamide) platinum(II) chloride ([Pt^{II}(dpb-hexyl)Cl]) 7·Pt *10*

Synthesis of (dihexyl 2,4-di-2-pyridyl-isophthalamide)(4-tolylterpyridine) ruthenium(II) hexafluorophosphate ([Ru^{II}(dpb-hexyl)(ttpy)PF6]) 7·Ru·PF$_6$ *11*

Synthesis of (2,4-di-2-pyridyl-isophthalamide macrocycle) platinum(II) chloride ([Pt^{II}(dpb-macrocycle)Cl]) 10·Pt *12*

Synthesis of (2,4-di-2-pyridyl-isophthalamide macrocycle)(4-tolylterpyridine) ruthenium(II) hexafluorophosphate ([Ru^{II}(dpb-macrocycle)(ttpy)PF$_6$]) 10·Ru·PF$_6$ *15*

Synthesis of [2]rotaxane (2,4-di-2-pyridyl-isophthalamide macrocycle) platinum(II) chloride ([Pt^{II}(dpb-macrocycle)Cl] [2]rotaxane) 12·Pt·Cl *17*

Synthesis of [2]rotaxane (2,4-di-2-pyridyl-isophthalamide)(4-tolylterpyridine) ruthenium(II) hexafluorophosphate ([Ru^{II}(dpb-macrocycle)(ttpy)PF$_6$] [2]rotaxane) 12·Ru·(PF$_6$)$_2$ *19*

2. UV-vis and luminescence data **22**

2.1 Absorption, Emission and Excitation profiles for novel receptors *22*

2.2 Luminescent Anion binding titrations *25*

2.2.1 Anion titrations with [PtII(dpb-hexyl)Cl] 7·Pt *25*

2.2.2 Anion titrations with [PtII(dpb-macrocycle)Cl] 10·Pt *28*

2.2.3 Anion titrations with [RuII(dpb-hexyl)(ttpy)]PF6 7·Ru·PF6 .... *31*

2.2.4 Anion titrations with [RuII(dpb-macrocycle)(ttpy)]PF6 10·Ru·PF6 .... *34*

2.2.5 Anion titrations with [2]rotaxane [Ru^{II}(dpb-macrocycle)(ttpy)]PF$_6$ 12·Ru·(PF$_6$)$_2$ *37*

3. ^{1}H NMR anion titration data **38**

4. Single crystal X-ray crystallography **38**

dimethyl-2,4-di-2-pyridyl-isophthalate ester 4 (CCDC 1978092) 39

[Ru^{II}(dpb-hexyl)(ttpy)]Cl 7·Ru·PF_6 (CCDC 1978093) 40

syn-[Ru^{II}(dpb-macrocycle)(ttpy)]PF_6 10·Ru·PF_6 (CCDC 1978096) 42

anti-[Ru^{II}(dpb-macrocycle)(ttpy)]PF_6 10·Ru·PF_6 (CCDC 1978101) 44

5. References **46**

1. Experimental and ^{1}H, ^{13}C NMR and HRMS spectra

General Considerations

Commercial grade chemicals and solvents were used without further purification. Where anhydrous solvents were used, they were degassed with N_2 and passed through an MBraun MPSP-800 column Where degassed solvents were used, they were degassed via bubbling of N_2 gas through the solution unless stated otherwise. Triethylamine was distilled from and stored over potassium hydroxide. De-ionised water dispensed from a Millipore Milli-Q purification system was used in all cases. Tetrabutylammonium (TBA) salts were stored under vacuum in a desiccator. Microwave reactions were carried out using a Biotage Initiator 2.0 microwave. All synthetic procedures have been reliably repeated multiple times. Routine 300 MHz NMR spectra were recorded on a Varian Mercury 300 spectrometer, ^{1}H NMR operating at 300 MHz, ^{13}C{^{1}H} at 76 MHz, ^{19}F at 283 MHz and ^{31}P at 121 MHz . Where the solubility of the compounds were too low, or not enough compound existed, a Bruker AVII500 with ^{13}C Cryoprobe spectrometer was used for obtaining ^{13}C{^{1}H} at 126 MHz, however in some cases a compete ^{13}C{^{1}H} spectrum could not be obtained. All 500 MHz ^{1}H Spectra and all ^{1}H NMR titrations were recorded on a Varian Unity Plus 500 spectrometer. All chemical shift (δ) values are given in parts per million and are referenced to the solvent. In cases where solvent mixtures are used, the main solvent is used as the reference. Where an apparent multiplet (*e.g.* app. t.) is quoted, J_{app} is given. Low resolution ESI mass spectra were recorded on a Micromass LCT Premier XE spectrometer. Accurate masses were determined to four decimal places using Bruker µTOF and Micromass GCT spectrometers. UV/visible experiments were carried out on a PG instruments T60U spectrometer at 293 K. Steady-state fluorescence spectra were recorded on JobinYvon-Horiba Fluorolog-3 spectrophotometer or a Varian Cary-Eclipse spectrometer.

2,4-dibromo-1,5-dimethylbenzene **1**,[1] 2-tributylstannylpyridine,[2] 4-tolyl-2,2′:6′,2′′-terpyridine,[3] 4-tolyl-2,2′:6′,2′′-terpyridine ruthenium trichloride,[4] diamine **8**,[5] 3,5-bis-hexylamide pyridinium chloride **9·Cl**[6] and pyridinium axle·Cl **11·Cl**,[7] were synthesised as previously described.

Synthesis of 2,4-dibromo-isophthalic acid 2

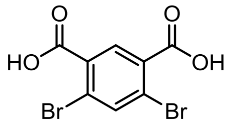

This compound is known and has been previously synthesised *via* a different procedure.[8]

KOH (3.20 g, 58.1 mmol) was added to a solution of 1,5-dibromo-2,4-dimethylbenzene **1** (15.0 g, 56.8 mmol) in water (300 mL). The resulting solution was diluted with pyridine (150 mL) and heated to 70 °C. 5.00 g portions of $KMnO_4$ were added in 12 hour intervals until no further decolourisation of the solution was observed upon its addition (*ca.* 2 weeks). The resulting solution was filtered through Celite and the solution concentrated to 150 mL *in vacuo.* This solution was acidified to pH = 5 with conc. $HCl_{(aq)}$. The precipitate was collected *via* filtration, washed with water (3 × 200 mL) and hexane (2 × 100 mL) and dried *in vacuo* to give a white solid (16.5 g, 89%, 50.9 mmol). ^{1}H NMR (300 MHz, CD_3OD) δ_H 8.27 (1H, s, ArH), 8.10 (1H, s, ArH); ^{13}C NMR (76 MHz, CD_3OD) δ_C 167.9, 140.8, 134.9, 133.6, 126.2; ESI-HRMS m/z calcd. for $[C_8H_4Br_2O_4 - H]^+$ 322.8384, found 322.8376.

^{1}H NMR spectrum (400 MHz, $CDCl_3$, 298 K)

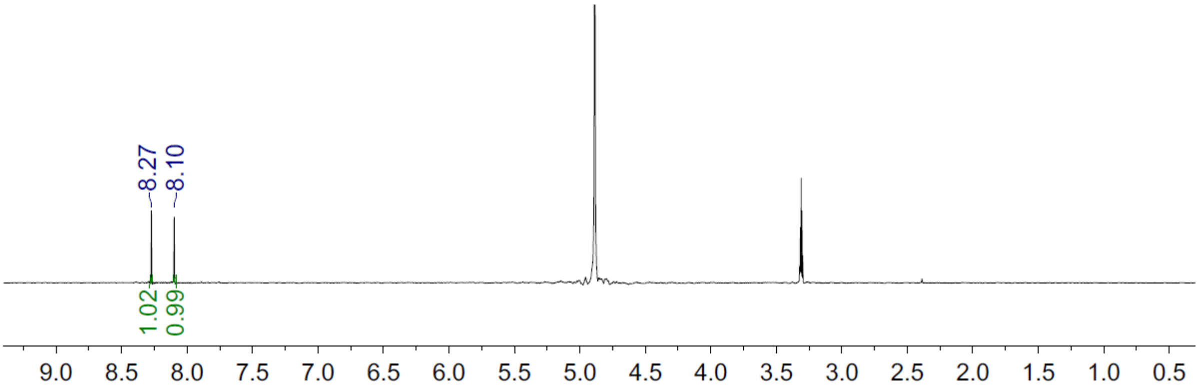

^{13}C NMR spectrum (76 MHz, $CDCl_3$, 298 K)

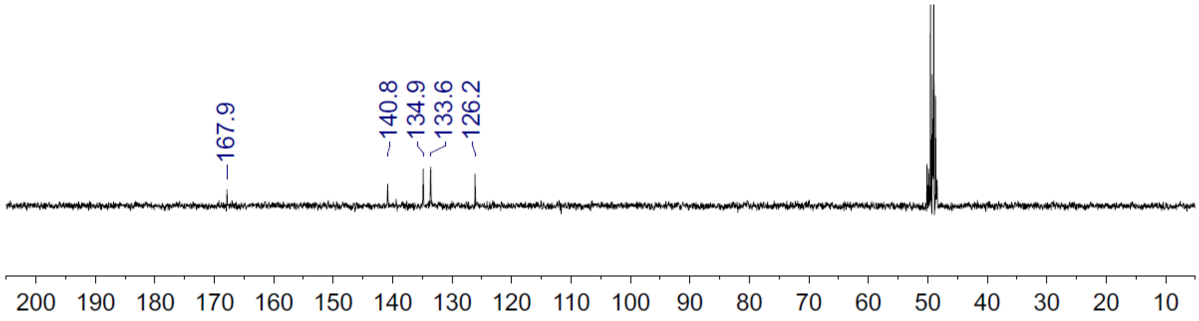

Synthesis of dimethyl-2,4-dibromo-isophthalate ester 3

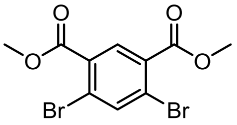

This compound is known and has been previously synthesised *via* a different procedure.[9]

5 drops of conc. $H_2SO_{4(aq)}$ were added to a solution of 4,6-dibromoisophthalic acid **2** (1.00 g, 3.11 mmol) in MeOH (15 mL) in a 20 mL microwave vial. The reaction mixture was subjected to microwave irradiation at 100 °C for 20 minutes. This was repeated four times and the reaction mixtures combined upon completion. The resulting solution was poured onto ice water, basified with 25 % $NH_4OH_{(aq)}$ and extracted with Et_2O (3 ×

100 mL). The combined organic layers were dried over anhydrous $MgSO_4$ and the solvent removed *in vacuo* to give a white solid (3.86 g, 89%, 10.9 mmol). ^{1}H NMR (300 MHz, $CDCl_3$) δ_H 8.29 (1H, s, ArH), 8.02 (1H, s, ArH), 3.94 (6H, s, CH_3); ^{13}C NMR (76 MHz, $CDCl_3$) δ_C 165.0, 140.0, 134.2, 130.8, 126.1, 53.0; FI-HRMS m/z calcd. for $[C_{10}H_8Br_2O_4]^+$ 351.8768, found 351.8769.

^{1}H NMR spectrum (400 MHz, CD_3OD, 298 K)

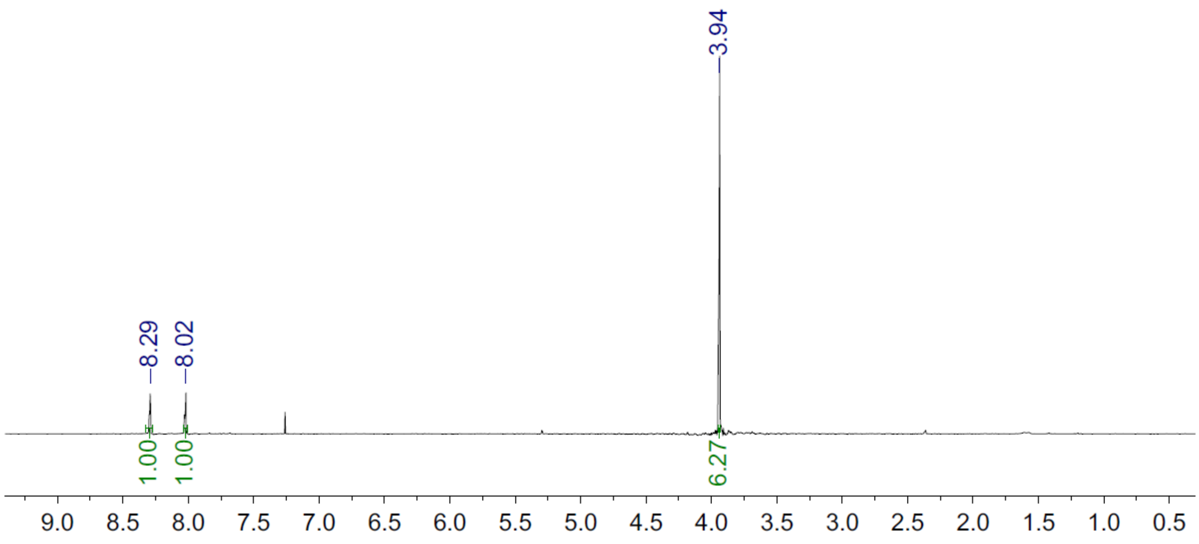

^{13}C NMR spectrum (76 MHz, CD_3OD, 298 K)

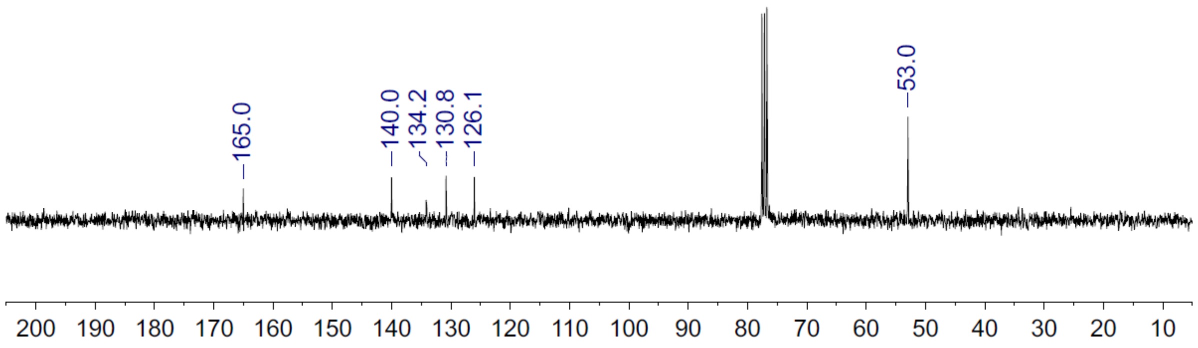

Synthesis of dimethyl-2,4-di-2-pyridyl-isophthalate ester (dpb-ester) 4

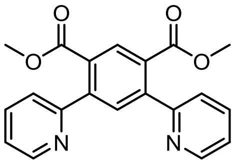

Dimethyl 2,4-dibromoisophthalate **3** (2.10 g, 5.96 mmol), 2-(tributylstannyl)pyridine (6.00 mL, 18.0 mmol), $Pd(PPh_3)_2Cl_2$ (0.20 g, 0.285 mmol) and LiCl (2.00 g, 47.2mmol) were dissolved in anhydrous toluene (100 mL), degassed *via* 3 freeze-pump-thaw cycles, and placed under N_2. The reaction mixture was then heated under reflux for 48 hours under N_2. The resulting solution was cooled, sat. $KF_{(aq)}$ (10 mL) was added and the solution stirred for 30 minutes. The solution was filtered through Celite, washed with water (50 mL) and toluene (50 mL) and extracted with CH_2Cl_2 after adding sat. $NaHCO_{3(aq)}$ (10 mL). Purification by column chromatography (SiO_2; hexane/Et_2O; 100:0→10:90) gave the product as an off-white solid (1.21 g, 58%, 3.47 mmol). ^{1}H NMR

(300 MHz, $CDCl_3$) δ_H 8.66 (2H, d, $^3J_{HH}$ = 5.3, ArH), 8.32 (1H, s, ArH), 7.78 – 7.68 (3H, m, ArH), 7.49 (2H, d, $^3J_{HH}$ = 7.6, ArH), 7.26 (2H, app. t, J_{app} = 6.5, Ar*H*), 3.74 (6H, s, C*H*$_3$); ^{13}C NMR (76 MHz, $CDCl_3$) δ_C 167.9, 157.2, 149.1, 143.4, 136.3, 131.5, 131.5, 131.0, 122.9, 122.6, 52.2; ESI-HRMS m/z calcd. for $[C_{20}H_{16}N_2O_4]^+$ 371.1002, found 371.1006.

^{1}H NMR spectrum (400 MHz, $CDCl_3$, 298 K)

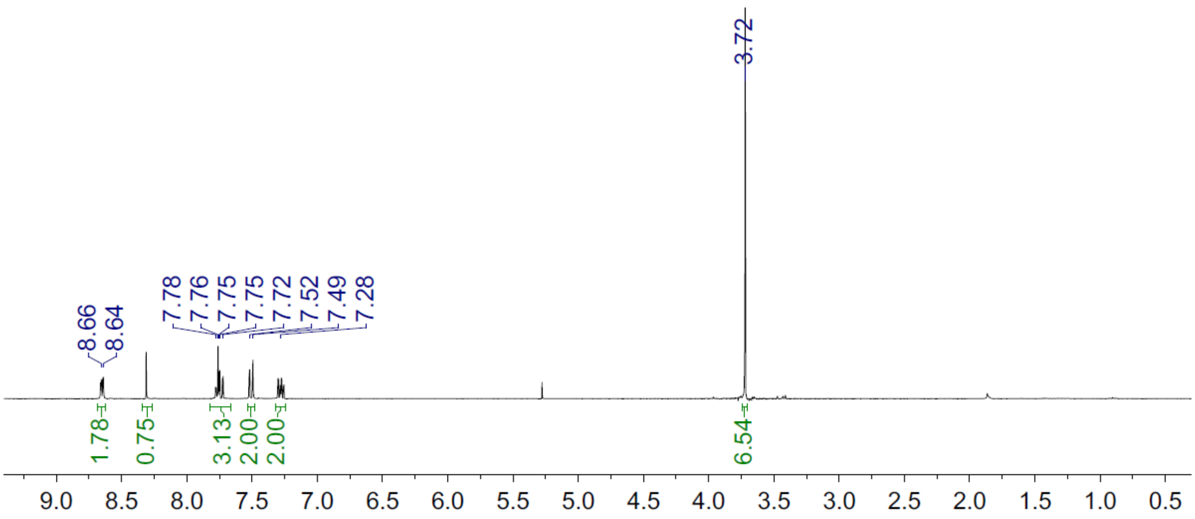

^{13}C NMR spectrum (76 MHz, $CDCl_3$, 298 K)

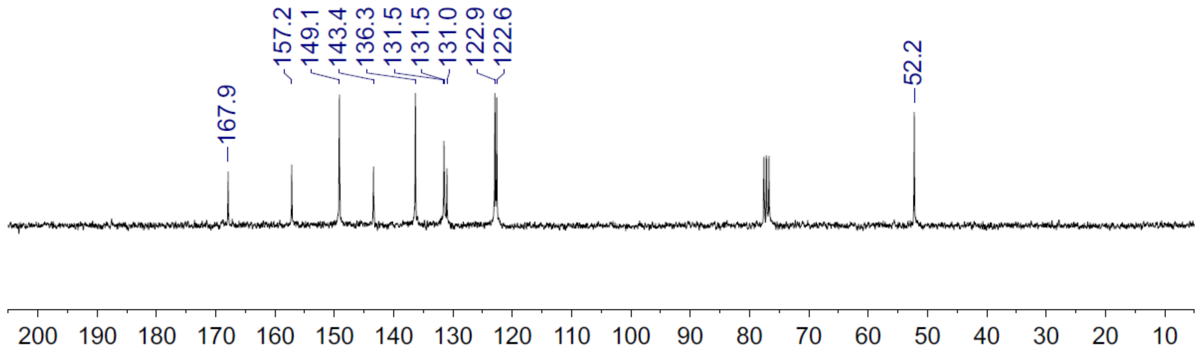

HRMS spectrum (ESI)

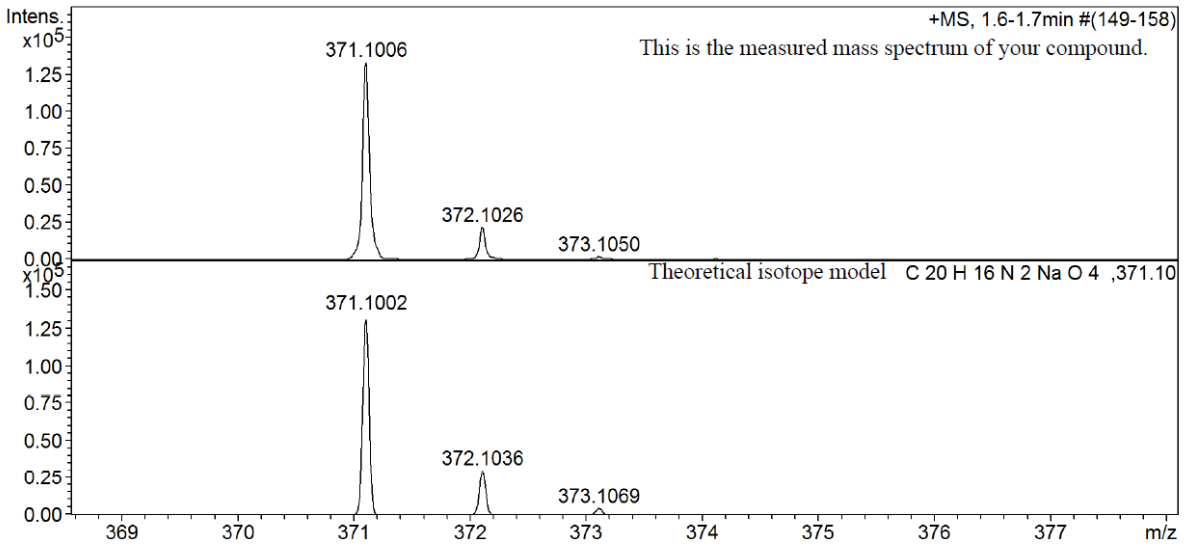

Synthesis of (dimethyl-2,4-di-2-pyridyl-isophthalate ester) platinum(II) chloride ([Pt^{II}(dpbester)Cl]) 5·Pt

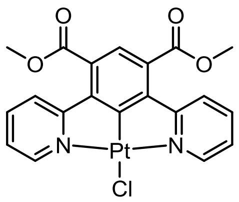

K_2PtCl_4 (50 mg, 0.1200 mmol) dissolved in H_2O (10 mL) was added to dimethyl 4,6-di(pyridin-2-yl)isophthalate **4** (42 mg, 0.120 mmol) in acetonitrile (30 mL). The reaction mixture was heated under vigorous reflux for 72 hours. The resulting solution was cooled, filtered and washed with H_2O (10 mL), EtOH (10 mL), acetonitrile (10 mL) and Et_2O (10 mL). The solvent was removed *in vacuo* and subsequent drying under high vacuum gave a yellow solid (45 mg, 64%, 78 µmol). ^{1}H NMR (500 MHz, d^6-DMSO) δ_H 9.38 (2H, d, $^3J_{HH}$ = 6.3, Ar<u>H</u>), 8.31 (2H, d, app. t, J_{app} = 7.5, Ar<u>H</u>), 8.12 (2H, d, $^3J_{HH}$ = 7.8, Ar<u>H</u>), 7.69 (2H, app. t, J_{app} = 7.2, Ar<u>H</u>), 7.56 (1H, s, Ar<u>H</u>); ^{13}C NMR (126 MHz, d^6-DMSO) δ_C 167.1, 163.3, 151.7, 140.6, 139.0, 129.5, 125.7, 124.4, 123.8, 123.6, 53.3; EI-HRMS m/z calcd. for $[C_{20}H_{15}ClN_2O_4Pt - Cl]^+$ 542.0680, found 542.0695.

^{1}H NMR spectrum (400 MHz, d^6-DMSO, 298 K)

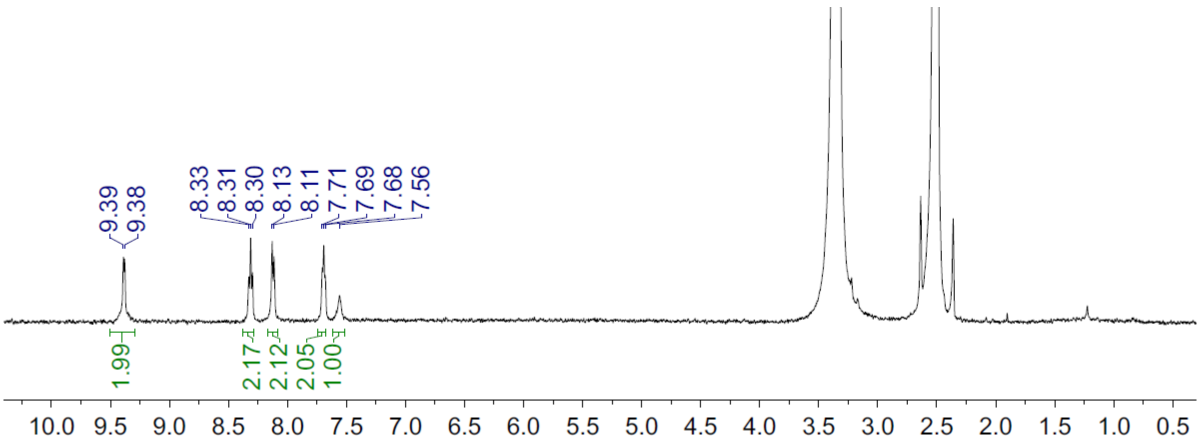

^{13}C NMR spectrum (76 MHz, d^6-DMSO, 298 K) CH_2Cl_2 indicated by *

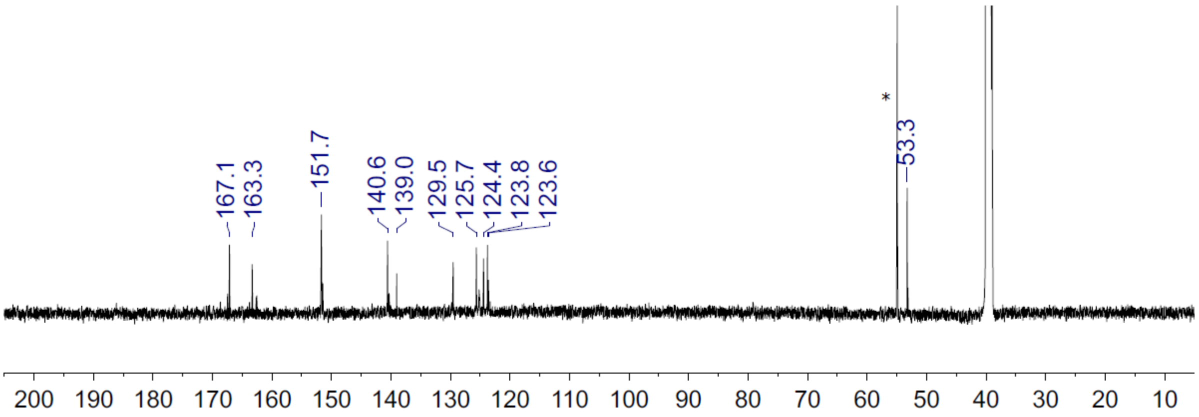

HRMS spectrum (EI)

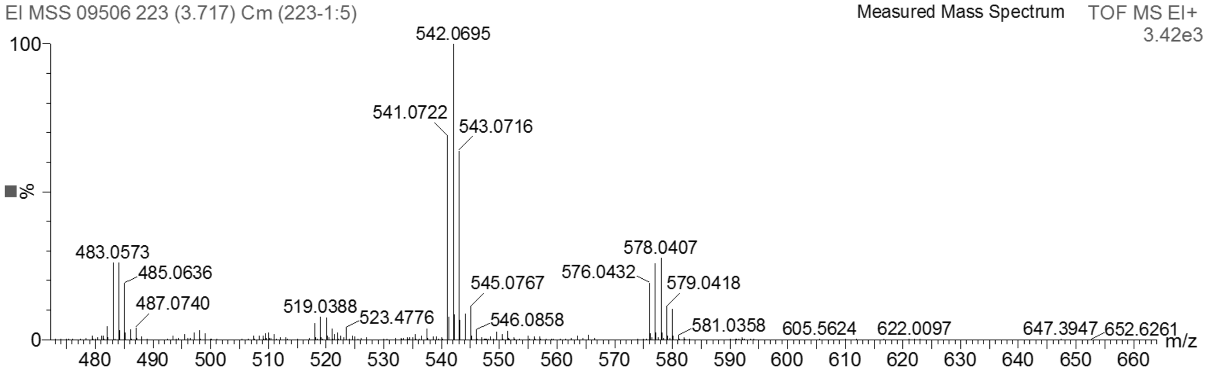

Synthesis of (dimethyl-2,4-di-2-pyridyl-isophthalate ester)(4-tolylterpyridine) ruthenium(II) hexafluorophosphate ($[Ru^{II}(dpb\text{-}ester)(ttpy)PF_6]$) 5·Ru·$PF_6$

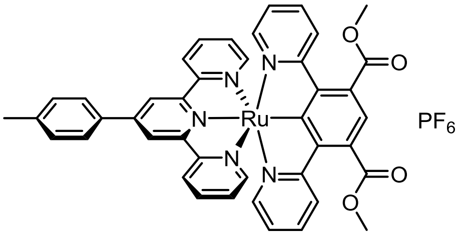

$RuCl_3$(ttpy) (0.200 g, 0.377 mmol) was added to a solution of $AgBF_4$ (0.265 g, 1.36 mmol) in acetone (40 mL) in the dark. The reaction mixture was heated under reflux for two hours. The resulting solution was cooled and filtered through Celite. The solvent was removed *in vacuo* and the solid dissolved in *n*-butanol (40 mL). Dimethyl 2,4-di(pyridin-2-yl)isophthalate **4** (0.210 g, 0.602 mmol) was added and heated to reflux for 18 hours under N_2. The solution was cooled and concentrated to 10 mL *in vacuo.* Sat. $NH_4PF_{6(aq)}$ (2 mL) was added and the solution filtered. The resulting solid was purified by column chromatography (SiO_2; CH_2Cl_2/MeOH; 98:2) to give the product as a purple solid (0.220 g, 76%, 0.24 mmol). ^1H NMR (300 MHz, $(CD_3)_2CO$) δ_H 10.16 (2H, s, ArH), 9.72 (2H, d, $^3J_{HH}$ = 8.2, ArH), 9.01 (4 H, app. dd, J_{app} = 17.7, 8.2, ArH), 8.72 – 8.62 (2H, m, ArH), 8.58 – 8.45 (3H, m, ArH), 8.32 (4H, app. dd, J_{app} = 17.7, 6.8, ArH), 8.05 – 7.84 (4H, m, ArH), 7.67 (2H, t, $^3J_{HH}$ = 6.5, ArH), 4.92 (6H, s, CO_2CH_3), 3.31 (3H, s, $ArCH_3$); ^{13}C NMR (76 MHz, $(CD_3)_2CO$) δ_C 170.8, 167.0, 160.0, 155.5, 154.1, 152.5, 136.5, 136.1, 131.0, 130.0, 129.1, 128.3, 127.6, 124.9, 124.3, 123.7, 121.0, 53.2, 21.3, remaining quaternary carbons not detected; ^{19}F NMR (283 MHz, $CDCl_3$) δ_F -73.0 (d, $^1J_{PF}$ = 713 Hz, PF_6); ^{31}P NMR (121 MHz, $CDCl_3$) δ_P -144.32 (sept., $^1J_{PF}$ = 714 Hz, PF_6); ESI-HRMS m/z calcd. for $[C_{42}H_{32}F_6N_5O_4PRu - PF_6]^+$ 772.1504, found 772.1502.

^{1}H NMR spectrum (300 MHz, $(CD_3)_2CO$, 298 K)

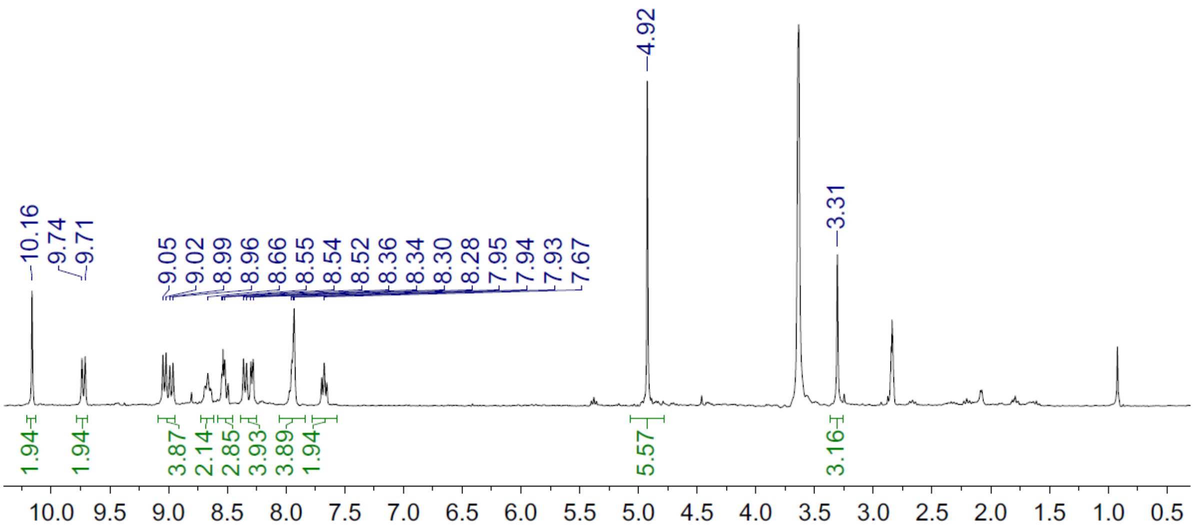

^{13}C NMR spectrum (76 MHz, $(CD_3)_2CO$, 298 K)

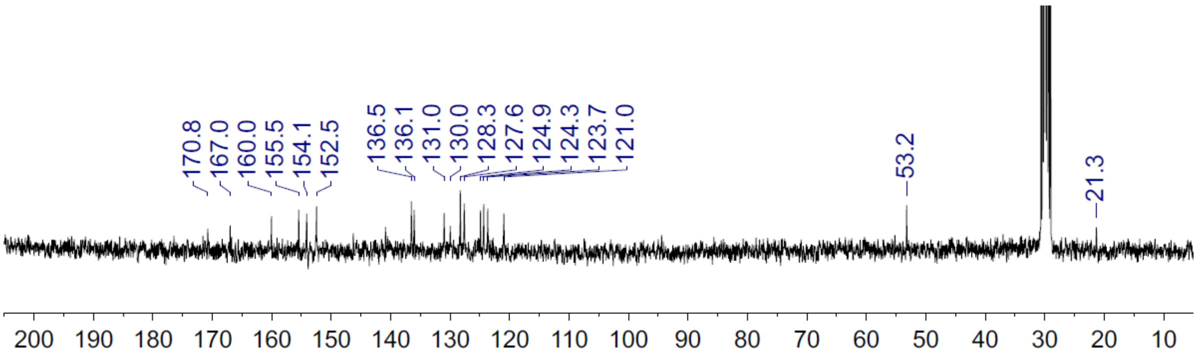

HRMS spectrum (ESI)

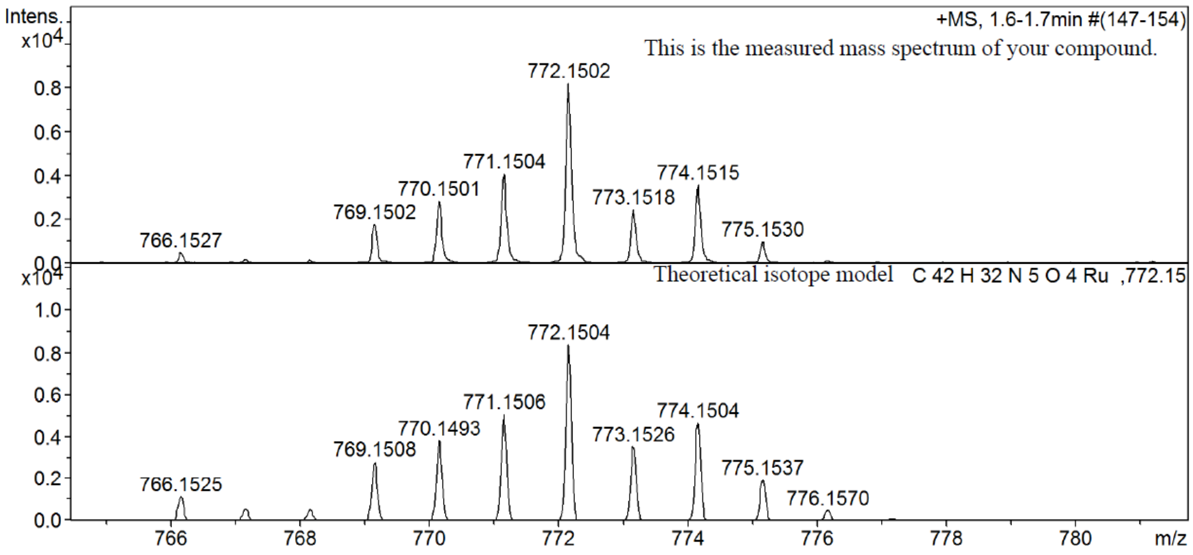

Synthesis of (2,4-di-2-pyridyl-isophthalic acid) platinum(II) chloride ([Pt^{II}(dpbacid)Cl]) 6·Pt

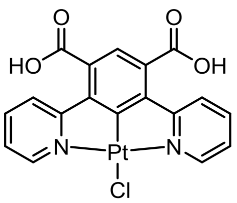

[Pt^{II}(dpbester)Cl] **5·Pt** (0.200 g, 0.344 mmol) was suspended in a solution of KOH (1.93 g, 34.0 mmol) dissolved in MeOH (5 mL). The reaction mixture was stirred at 40 °C for 18 hours. H_2O (2 mL) was added and the MeOH was removed *in vacuo*. The resulting solution was neutralised with conc. $HCl_{(aq)}$, filtered and washed with H_2O (2 × 5 mL) and MeOH (2 × 5 mL). The yellow precipitate was dried *in vacuo* to give a yellow solid, the platinum bis-acid intermediate(186 mg, 99%, 0.339 mmol). This was used in the next step without characterisation due to extremely low solubility.

Synthesis of (2,4-di-2-pyridyl-isophthic acid)(4-tolylterpyridine) ruthenium(II) hexafluorophosphate ([Ru^{II}(dpb-acid)(ttpy)PF_6]) 6·Ru·PF_6

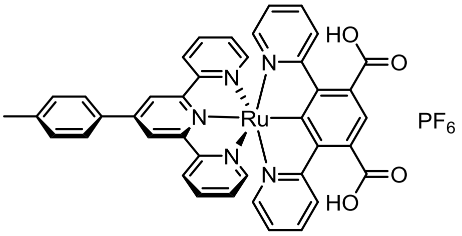

10 % w/w $NaOH_{(aq)}$ (0.9 mL) was added to a solution of [Ru^{II}(dpb-ester)(ttpy)PF_6] **5·Ru·PF_6** (10 mg, 12 µmol) dissolved in dioxane (6.7 mL) and MeOH (2.4 mL). The reaction mixture was stirred at ambient temperature for 18 hours. H_2O (0.5 mL) was added and MeOH and dioxane were removed *in vacuo*. The resulting solution was then neutralised with conc. $HCl_{(aq)}$, cooled for 30 minutes at 4 °C and filtered, and washed with H_2O (2 × 2 mL) and Et_2O (2 × 2 mL). The solvent was removed *in vacuo* and subsequent drying under high vacuum gave the compounds as a purple solid (5.1 mg, 52%). Due to solubility issues, this was not characterised and used in the next step without further purification. ^1H NMR (500 MHz, CD_3CN) δ_H 8.99 (2H, s, ArH), 8.57 (2H, d, $^3J_{HH}$ = 7.0, ArH), 8.31 (2H, d, $^3J_{HH}$ = 7.8, ArH), 8.11 (2H, d, $^3J_{HH}$ = 7.8, Ar*H*), 7.76 (1H, s, ArH), 7.73 (2H, app. t, J_{app} = 7.8, ArH), 7.64 (2H, app. t, J_{app} = 7.3, ArH), 7.56 (2H, d, $^3J_{HH}$ = 6.7, ArH) 7.28 (2H, d, $^3J_{HH}$ = 4.9, ArH), 7.04 (2H, d, $^3J_{HH}$ = 6.1, ArH), 6.97 (2H, app. t, J_{app} = 5.8, ArH), 6.77 (2H, app. t, J_{app} = 5.8, ArH), 2.52 (3H, s, CH_3); ESI-LRMS m/z calcd. for $[C_{40}H_{28}F_6N_5O_4PRu - PF_6]^+$ 744.1, found 744.1.

^{1}H NMR spectrum (400 MHz, CD_3OD, 298 K) Et_2O indicated by *

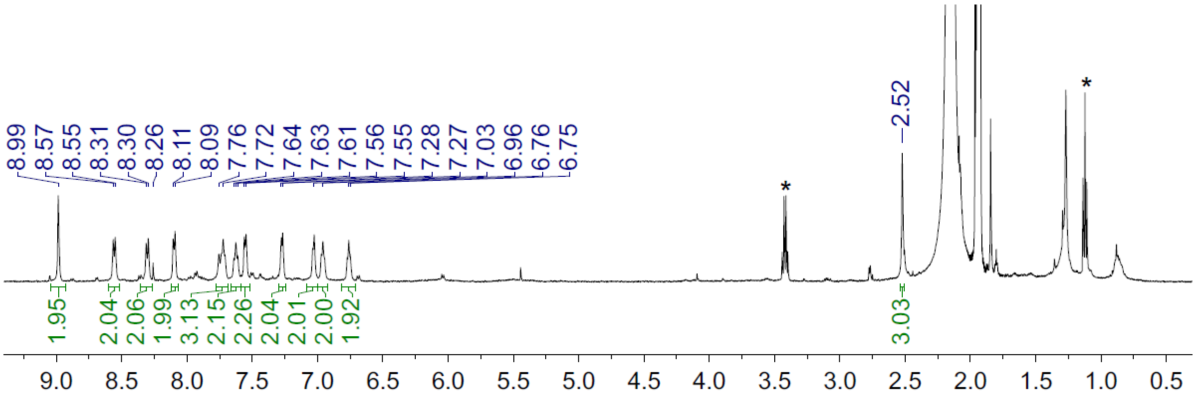

Synthesis of (dihexyl-2,4-di-2-pyridyl-isophthalamide) platinum(II) chloride ([Pt^{II}(dpb-hexyl)Cl]) 7·Pt

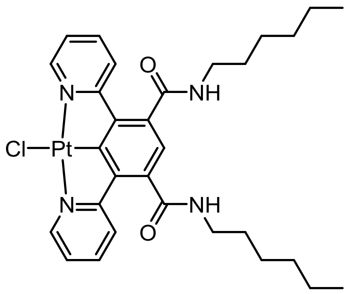

Oxalyl chloride (0.110 mL, 1.37 mmol) was added dropwise to a suspension of Pt^{II}(dpb-acid)Cl **6·Pt** (0.189 g, 0.343 mmol,) in anhydrous CH_2Cl_2 (100 mL) and anhydrous degassed catalytic DMF (1 drop). The reaction mixture was stirred under N_2 at ambient temperature until homogenous (*ca.* 6 hours). The solvent was removed *in vacuo* and dried under high vacuum. Anhydrous, distilled Et_3N (0.384 mL, 2.75 mmol) and hexylamine (0.270 mL, 2.06 mmol) were added to the acid chloride intermediate dissolved in CH_2Cl_2 (100 mL) at 0 °C. The reaction mixture was stirred for two hours at ambient temperature. The resulting solution was washed with sat. $NaHCO_{3(aq)}$ (50 mL), 10 % w/w citric acid (50 mL) and sat. brine (50 mL) and dried over anhydrous $MgSO_4$. The solvent was removed *in vacuo*. Purification by preparative thin-layer chromatography (SiO_2; CH_2Cl_2/MeOH; 90:10) gave a yellow solid (15.2 mg, 6%, 22 µmol). ^{1}H NMR (500 MHz, $CDCl_3$) δ_H 9.55 (2H, d, $^4J_{HH}$ = 5.8, ArH), 9.13 (2H, t, $^3J_{HH}$ = 5.6, NH), 8.47 (2H, app. t, J_{app} = 8.0, ArH), 8.10 (2H, d, $^3J_{HH}$ = 8.3, ArH), 7.87 (2H, app. t, J_{app} = 6.6, ArH), 1.45 – 1.56 (4H, m, NHCH$_2$), 1.20 – 1.38 (16H, m, CH$_2$), 0.69 – 0.86 (6H, m, CH$_3$); ^{13}C NMR (76 MHz, $CDCl_3$) δ_C 168.6, 164.3, 151.3, 137.7, 136.8, 134.2, 123.7, 122.4, 122.0, 40.1, 31.7, 29.6, 27.0, 22.8, 14.3; EI-HRMS m/z calcd. for $[C_{30}H_{37}ClN_4O_4Pt - Cl]^+$ 680.2564, found 680.2791; UV-Vis $\lambda_{max}(CH_2Cl_2)$/nm; ε/dm^3 mol^{-1} cm^{-1}: 251 (48,100) 295 (29,700) 364 (5,800) 379 (8,600) 416 (9,000).

^{1}H NMR spectrum (500 MHz, $CDCl_3$, 298 K)

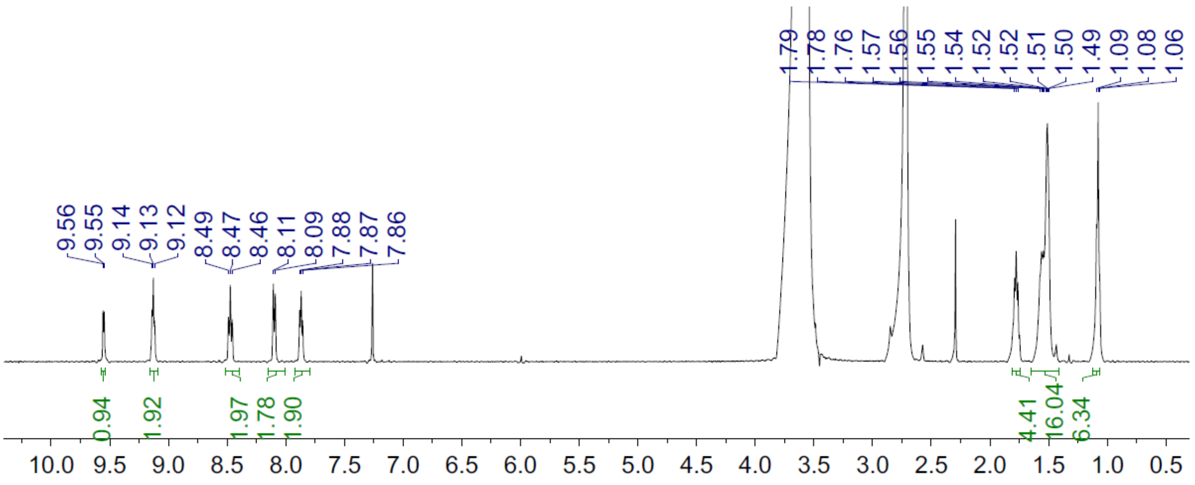

^{13}C NMR spectrum (76 MHz, $CDCl_3$, 298 K)

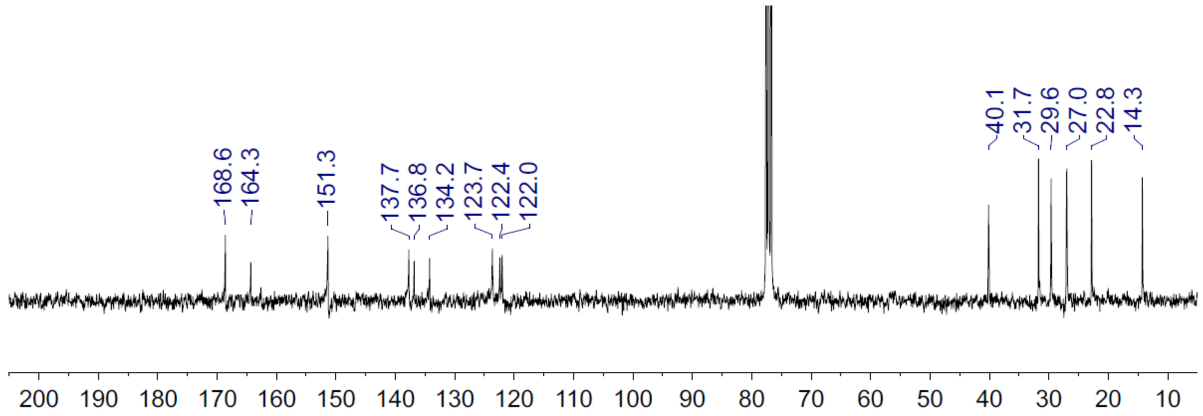

HRMS spectrum (EI)

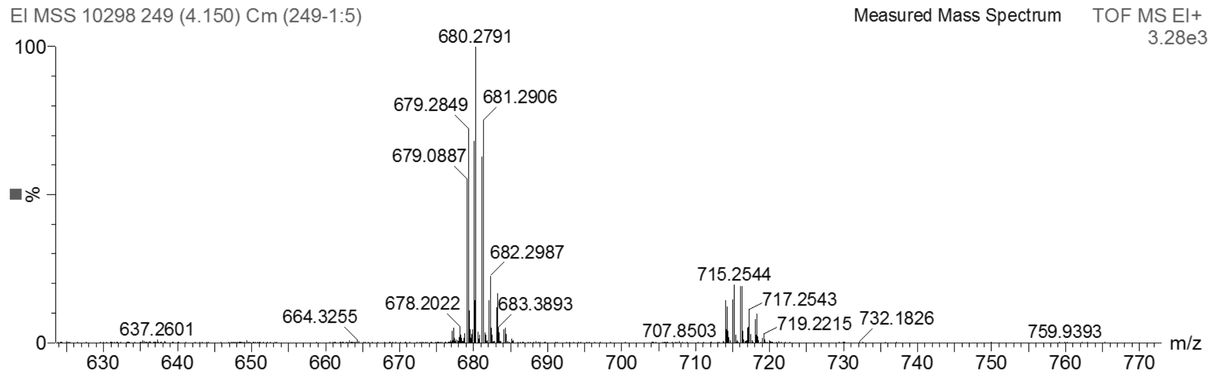

Synthesis of (dihexyl 2,4-di-2-pyridyl-isophthalamide)(4-tolylterpyridine) ruthenium(II) hexafluorophosphate ([Ru^{II}(dpb-hexyl)(ttpy)PF6]) 7·Ru·PF$_6$

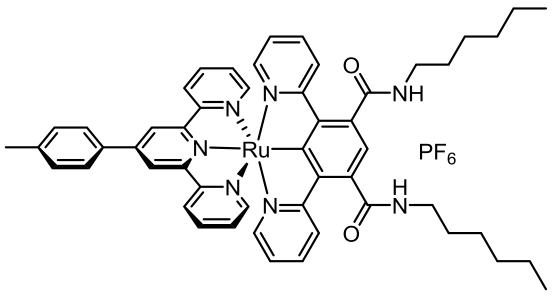

Oxalyl chloride (0.00400 mL, 0.0520 mmol) was added dropwise to a solution of [Ru^{II}(dpb-acid)(ttpy)PF$_6$] **6·Ru·PF$_6$** (9.6 mg, 0.0130 mmol) in anhydrous CH_2Cl_2 (50 mL) and anhydrous degassed catalytic DMF (1 drop). The reaction mixture was stirred under N_2 at ambient temperature until homogenous. The solvent was removed *in vacuo* and dried under high vacuum. Anhydrous, distilled Et_3N (0.0140 mL, 0.104 mmol) and hexylamine (0.0100 mL, 0.0780 mmol) were added to the acid chloride intermediate dissolved in anhydrous CH_2Cl_2 (50 mL) at 0 °C. The reaction mixture was stirred for two hours at ambient temperature under N_2. The solvent was then removed *in vacuo* and the solid was dissolved in CH_2Cl_2 (15 mL) and washed with 0.1 M $NH_4PF_{6(aq)}$ (10 × 10 mL) and H_2O (2 × 10 mL). The organic layer was dried over $MgSO_4$, and the solvent removed *in vacuo*. Purification by preparative thin-layer chromatography (SiO_2; CH_2Cl_2/MeOH; 90:10) gave a purple solid (9.24 mg, 81 %). ^{1}H NMR (500 MHz, CD_2Cl_2) δ_H 8.77 (2H, s, ArH), 8.35 (2H, app. dt, *J*=8.1, 1.2, ArH), 8.28 (2H, app. dd, *J*=8.4, 1.1, ArH), 8.01 – 7.93 (2H, m, ArH), 7.65 (2H, app. td, *J*=7.8, 1.5, ArH), 7.58 – 7.47 (4H, m, ArH), 7.29 (1H, s, ArH), 7.28 – 7.23 (2H, m, ArH), 7.09 – 7.05 (2H, m, ArH), 6.99 (2H, app. ddd, *J*=7.3, 5.6, 1.4, ArH),

6.83 (2H, t, *J*=5.8, ArH), 6.63 (2H, app. ddd, *J*=7.2, 5.7, 1.4, ArH), 3.58 (4H, app. td, *J*=7.3, 5.8, NCH_2), 2.54 (3H, s, CH_3), 1.77 – 1.68 (4H, m, CH_2), 1.52 – 1.43 (4H, m, CH_2), 1.33 – 1.39 (8H, m CH_2), 0.92 – 0.88 (6H, m, CH_3); ^{13}C NMR (126 MHz, $CDCl_3$) δ_C 171.6, 167.6, 157.9, 156.6, 153.1, 150.2, 135.2, 134.5, 130.7, 127.2, 123.6, 122.5, 121.6, 119.2, 53.6, 46.0, 40.5, 31.7, 29.9, 29.4, 26.9, 22.8, 21.6, 15.4, 14.2, remaining quaternary carbons undetected; ^{19}F NMR (283 MHz, $CDCl_3$) δ_F -72.5 (d, ^1J = 713 Hz, PF_6); ^{31}P NMR (121 MHz, $CDCl_3$) δ_P -144.53 (sept., ^1J = 713 Hz, PF_6); ESI-HRMS m/z calcd. for $[C_{52}H_{54}F_6N_7O_2PRu - PF_6]^+$ 910.3391, found 910.3390; UV-Vis $\lambda_{max}(CH_2Cl_2)$/nm; ε/dm^3 mol^{-1} cm^{-1}: 241 (39,900) 289 (64,800) 374 (9,600) 616 (14,200) 549 (12,800)

Synthesis of (2,4-di-2-pyridyl-isophthalamide macrocycle) platinum(II) chloride ([Pt^{II}(dpb-macrocycle)Cl]) 10·Pt

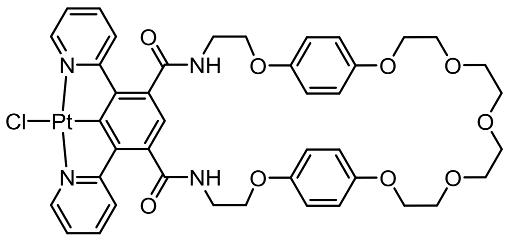

Oxalyl chloride (11 µL, 0.138 mmol) was added dropwise to a solution of Pt^{II}(dpbacid)Cl **6·Pt** (19.0 mg, 0.0346 mmol) in anhydrous CH_2Cl_2 (100 mL) and anhydrous degassed catalytic DMF (1 drop). The reaction mixture was stirred under N_2 at ambient temperature until homogenous (*ca.* 6 hours). The solvent was removed *in vacuo* and dried under high vacuum. Bis-amine (0.0160 g, 0.0346 mmol), pyridinium thread **9**·Cl (0.0130 g, 0.0346 mmol) and anhydrous, distilled Et_3N (0.0120 mL, 0.0865 mmol) were dissolved in anhydrous CH_2Cl_2 (100 mL) and stirred until homogenous. The acid chloride in anhydrous CH_2Cl_2 (100 mL) was added dropwise, and the reaction mixture stirred for three hours at ambient temperature under N_2. The solvent was removed *in vacuo* and the resulting solid was purified by preparative thin-layer chromatography (SiO_2; CH_2Cl_2/MeOH; 98:2) to give a yellow solid (8.1 mg, 24%, 8.2 µmol). ^{1}H NMR (500 MHz, DMSO-*d6*) δ_H 9.35 (2H, d, $^3J_{HH}$ = 5.6, ArH), 9.17 (2H, t, $^3J_{HH}$ = 5.6 Hz, NH), 8.28 (2H, app. t, J_{app} = 7.6, ArH), 7.95 (2H, d, $^3J_{HH}$ = 7.4, ArH), 7.67 (2H, app. t, J_{app} = 7.5, ArH), 7.12 (1H, s, ArH), 6.91 (4H, d, $^3J_{HH}$ = 9.0, ArH), 6.84 (4H, d, $^3J_{HH}$ = 9.0, ArH), 4.11 (4H, t, $^3J_{HH}$ = 5.4, CH_2), 3.97 (4H, t, $^3J_{HH}$ = 4.8, CH_2), 3.65 — 3.75 (8H, m, CH_2), 3.48—3.59 (8H, m, CH_2); ^{13}C NMR (126 MHz, DMSO-*d6*) δ_C 167.9, 164.4, 152.8, 152.4, 140.6, 136.7, 135.3, 124.9, 123.1, 122.5, 115.6, 115.3, 70.0, 69.0, 69.0, 67.7, 66.5, 30.7; MALDI-TOF MS m/z calcd. for $[C_{42}H_{43}ClN_4O_9Pt - Cl]^+$ 942.27, found 942.02; UV-Vis $\lambda_{max}(CH_2Cl_2)$/nm; ε/dm^3 mol^{-1} cm^{-1}: 234 (69,000) 253 (60,600), 295 (49,600) 361 (8,400) 380 (9,000) 419 (10,000).

^{1}H NMR spectrum (400 MHz, DMSO-*d6*, 298 K)

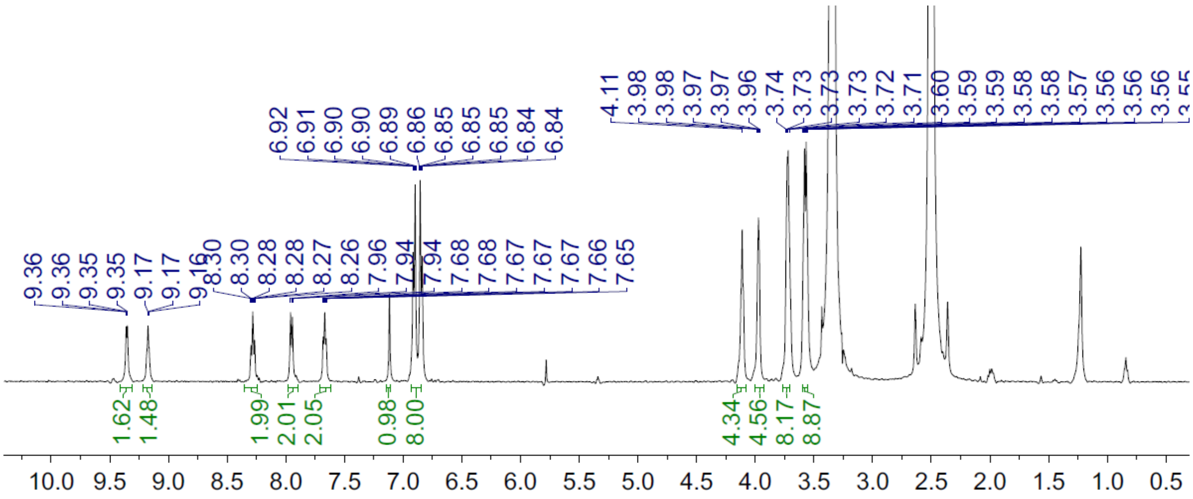

^{13}C NMR spectrum (76 MHz, DMSO-*d_6*, 298 K)

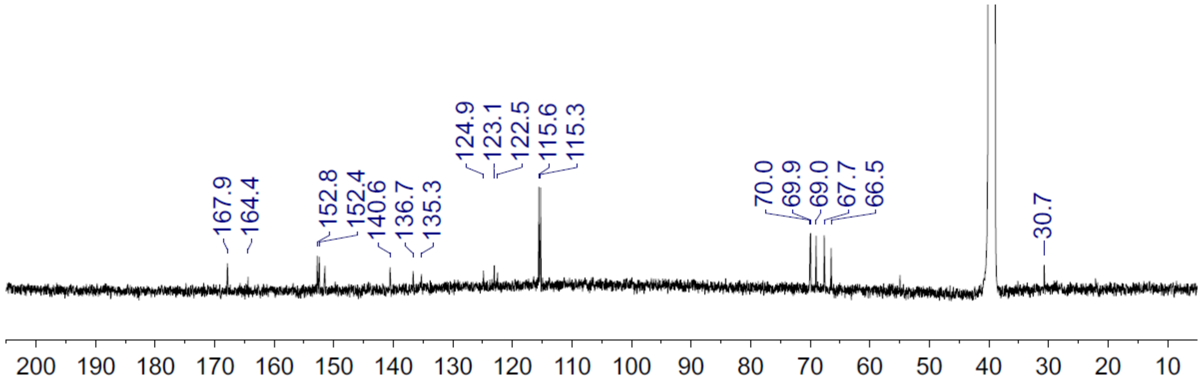

MS spectrum (MALDI-TOF)

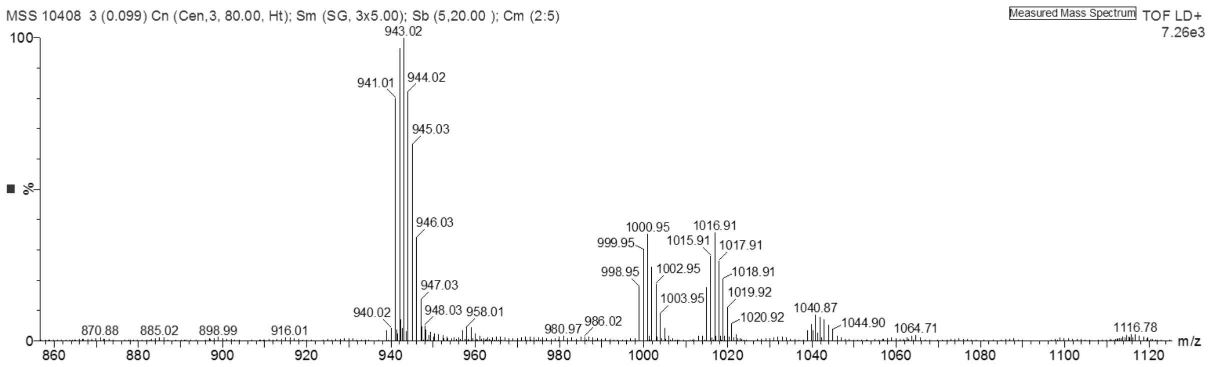

^{1}H NMR spectrum (500 MHz, CD_2Cl_2, 298 K)

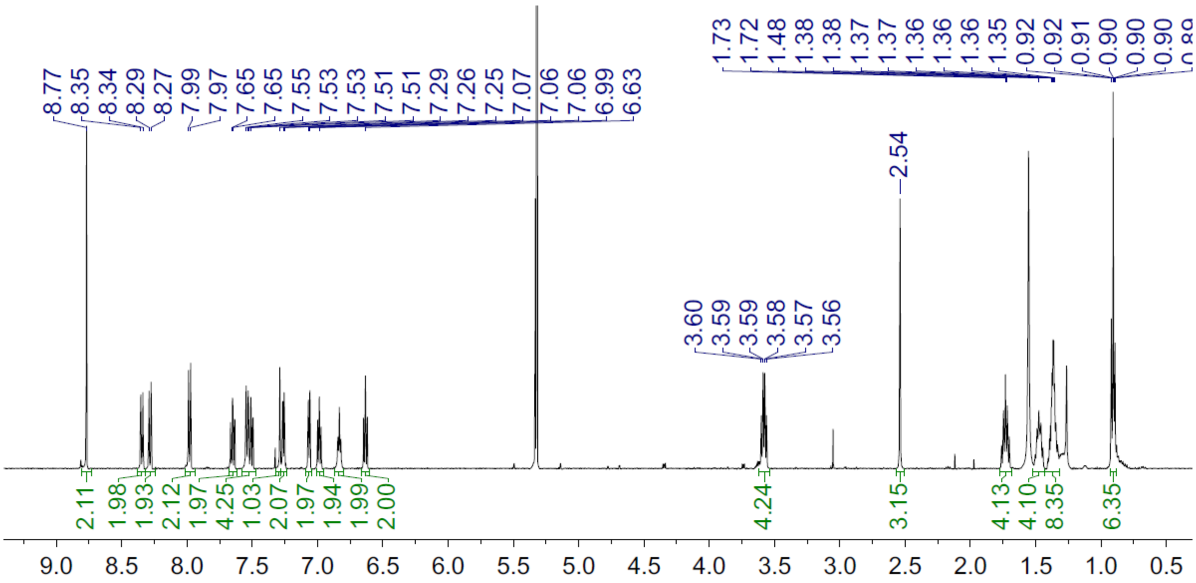

^{13}C NMR spectrum (126 MHz, $CDCl_3$, 298 K)

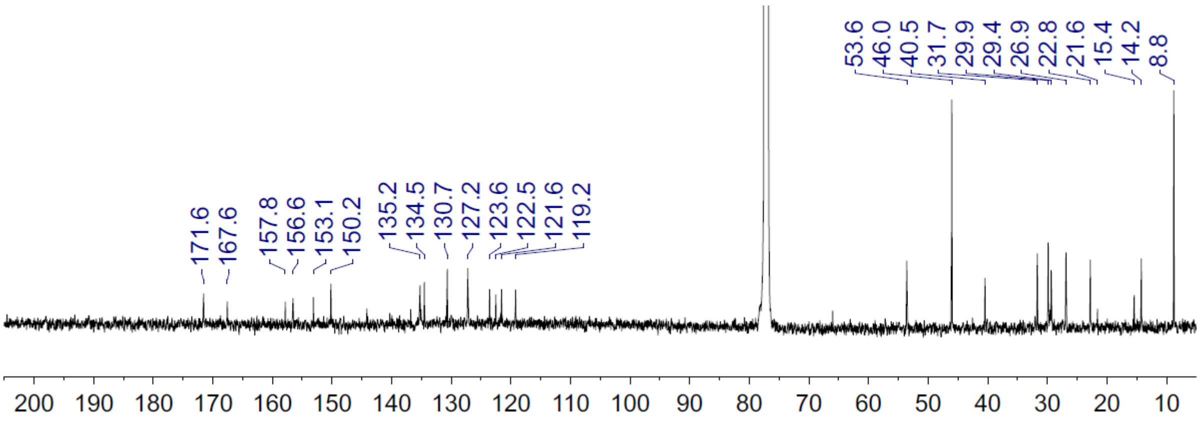

HRMS spectrum (ESI)

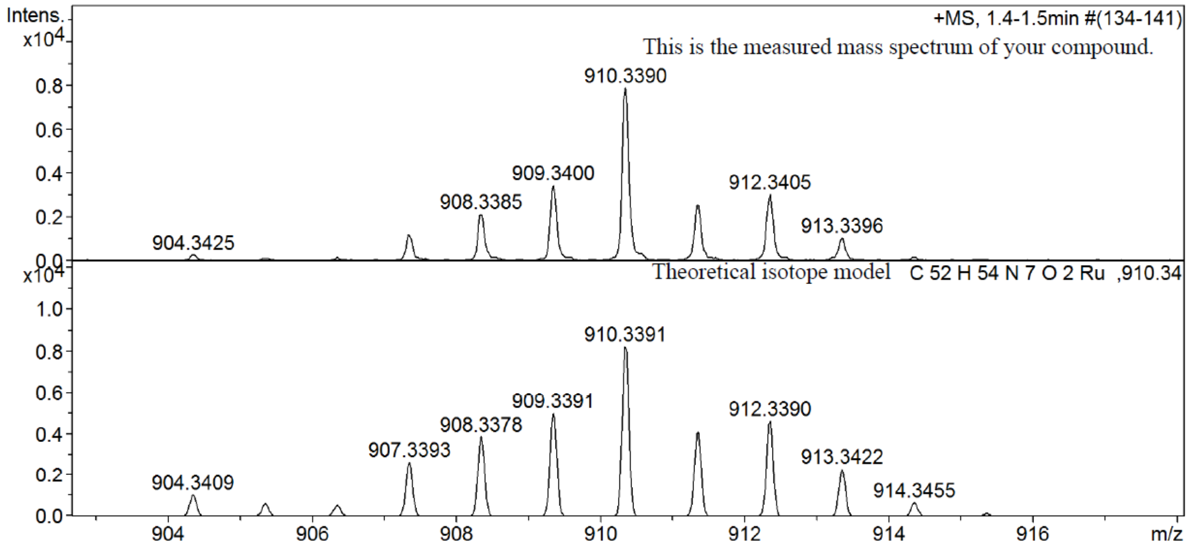

Synthesis of (2,4-di-2-pyridyl-isophthalamide macrocycle)(4-tolylterpyridine) ruthenium(II) hexafluorophosphate ([Ru^{II}(dpb-macrocycle)(ttpy)PF_6]) 10·Ru·PF_6

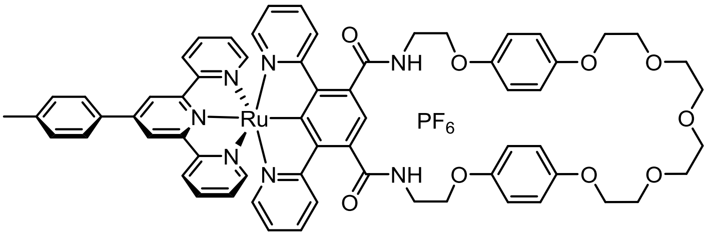

Oxalyl chloride (0.0400 mL, 0.450 mmol) was added dropwise to a solution of [Ru^{II}(dpb-acid)(ttpy)PF_6] **6·Ru·PF_6** (0.100 g, 0.112 mmol) in anhydrous CH_2Cl_2 (100 mL) and anhydrous degassed catalytic DMF (1 drop). The reaction mixture was stirred under N_2 at ambient temperature until homogenous (*ca.* 6 hours). The solvent was removed *in vacuo* and dried under high vacuum. Bis-amine **8** (0.0521 g, 0.112 mmol), pyridinium thread **9·Cl** (0.0430 g, 0.112 mmol) and anhydrous, distilled Et_3N (0.0400 mL, 0.281 mmol) were dissolved in anhydrous CH_2Cl_2 (100 mL) and stirred until homogenous. The acid chloride in anhydrous CH_2Cl_2 (100 mL) was added dropwise, and the reaction mixture stirred for three hours at ambient tmperature under N_2. The solvent was removed *in vacuo*. The resulting solid was dissolved in CH_2Cl_2 (15 mL) and washed with 0.1 M $NH_4PF_{6(aq)}$ (10 × 10 mL) and H_2O (2 × 10 mL). The organic layer was dried over $MgSO_4$, and the solvent removed *in vacuo*. The resulting solid was purified by column chromatography (SiO_2; CH_2Cl_2/MeOH; 96:4) and preparative thin-layer chromatography (SiO_2; CH_2Cl_2/MeOH; 98:2) to give a purple solid (71.5 mg, 48%, 54.2 µmol). ^1H NMR (500 MHz, $CDCl_3$) δ_H 9.47 (2H, t, $^3J_{HH}$ = 5.6, Ar$\underline{H}$), 8.76 (2H, s, Ar$\underline{H}$), 8.55 (2H, d, $^3J_{HH}$ = 8.5, Ar$\underline{H}$), 8.35 (2H, d, $^3J_{HH}$ = 8.1, Ar$\underline{H}$), 7.97 (2H, d, $^3J_{HH}$ = 7.8, Ar$\underline{H}$), 7.59 (1H, s, Ar$\underline{H}$), 7.55 (2H, t, $^3J_{HH}$ = 7.9, Ar$\underline{H}$), 7.50 (2H, d, $^3J_{HH}$ = 7.9, Ar$\underline{H}$), 7.44 (2H, d, $^3J_{HH}$ = 5.5, Ar$\underline{H}$), 7.37 (2H, t, $^3J_{HH}$ = 8.0, Ar$\underline{H}$), 7.00 (2H, t, $^3J_{HH}$ = 6.7, Ar$\underline{H}$), 6.91 – 6.84 (6H, m, Ar$\underline{H}$), 6.62 (4H, d, $^3J_{HH}$ = 8.6, Ar$\underline{H}$), 6.50 (2H, t, $^3J_{HH}$ = 6.6, Ar$\underline{H}$), 4.27 (4H, t, $^3J_{HH}$ = 6.1, C$\underline{H}_2$), 3.94 (4H, app. q, J_{app} = 6.0, C$\underline{H}_2$), 3.84 (4H, t, $^3J_{HH}$ = 5.0, C$\underline{H}_2$), 3.77 (4H, t, $^3J_{HH}$ = 4.9, C$\underline{H}_2$), 3.71 (8H s, C$\underline{H}_2$), 2.50 (3H, s, C$\underline{H}_3$); ^{13}C NMR (126 MHz, $CDCl_3$) δ_c 165.52, 158.31, 153.86, 151.94, 148.64, 147.04, 145.48, 145.03, 143.62, 136.70, 135.15, 134.87, 133.85, 131.99, 131.22, 130.77, 127.52, 125.96, 124.45, 120.58, 116.30, 115.01, 114.87, 70.90, 70.74, 70.23, 68.50, 65.86, 63.97, 34.48, 31.55; ^{19}F NMR (283 MHz, $CDCl_3$) δ_F -71.96 (d, ^1J = 713 Hz, PF_6); ^{31}P NMR (121 MHz, $CDCl_3$) δ_P -144.31 (sept., $^1J_{PF}$ = 714 Hz, $\underline{P}F_6$); ESI-HRMS m/z calcd. for $[C_{64}H_{60}F_6N_7O_9PRu - PF_6]^+$ 1172.3508, found 1172.3528; UV-Vis λ_{max}(CH_2Cl_2)/nm; ε/dm^3 mol^{-1} cm^{-1}: 233 (40,400) 288 (64,300) 373 (9,300) 516 (12,900) 554 (11,700)

^{1}H NMR spectrum (400 MHz, CD_3OD, 298 K)

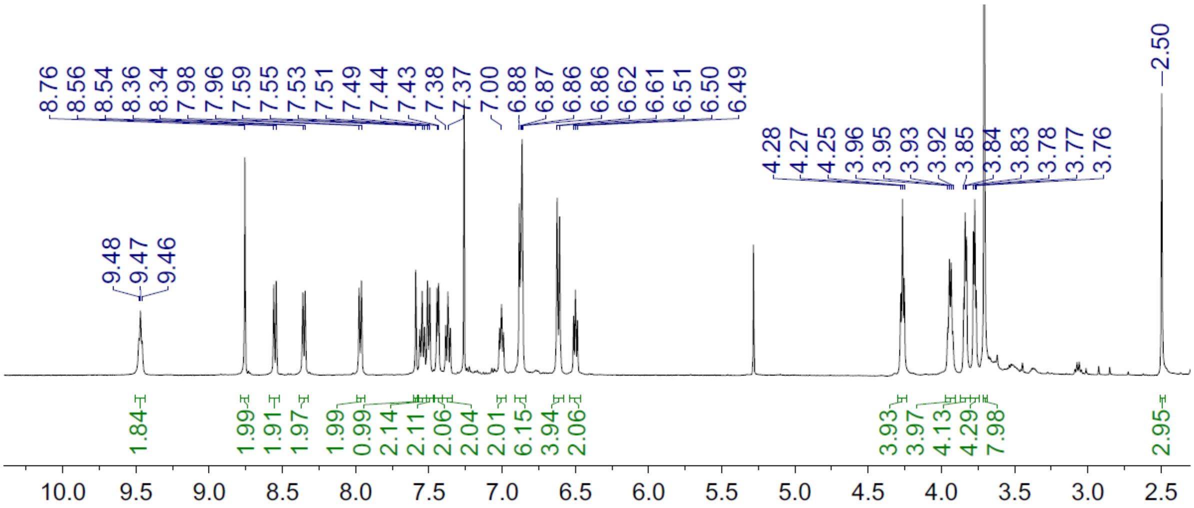

^{13}C NMR spectrum (76 MHz, CD_3OD, 298 K)

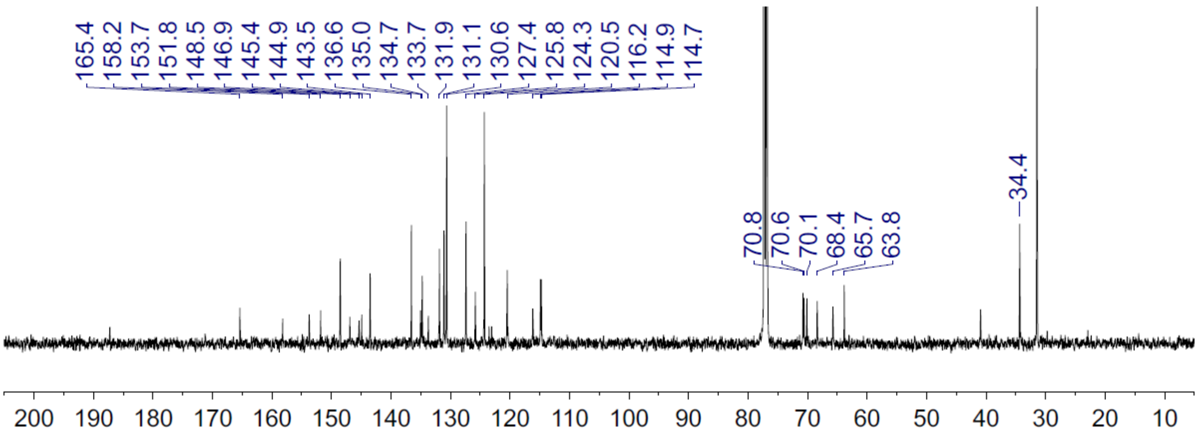

HRMS spectrum (ESI)

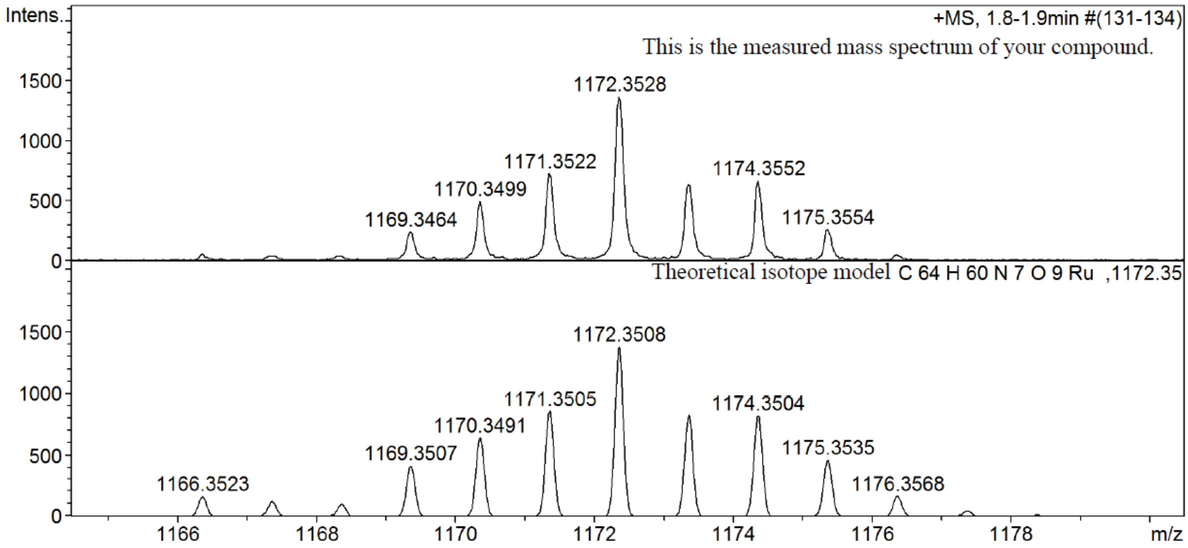

Synthesis of [2]rotaxane (2,4-di-2-pyridyl-isophthalamide macrocycle) platinum(II) chloride ([Pt^{II}(dpb-macrocycle)Cl] [2]rotaxane) 12·Pt·Cl

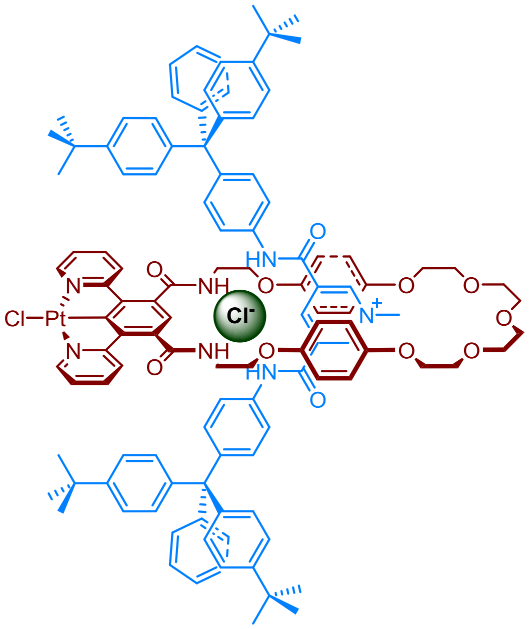

Oxalyl chloride (0.0400 mL, 0.496 mmol) was added dropwise to a solution of Pt^{II}(dpb-acid)Cl **6·Pt** (0.0682 g, 0.124 mmol) in anhydrous CH_2Cl_2 (100 mL) and anhydrous degassed catalytic DMF (1 drop). The reaction mixture was stirred under N_2 at 40 °C until homogenous (*ca.* 6 hours). The solvent was removed *in vacuo* and dried under high vacuum. Bis-amine **8** (0.0576 g, 0.124 mmol), pyridinium axle **11·Cl** (0.133 g, 0.124 mmol) and anhydrous, distilled Et_3N (0.043 mL, 0.310 mmol) were dissolved in anhydrous CH_2Cl_2 (100 mL) and stirred until homogenous. The acid chloride in anhydrous CH_2Cl_2 (100 mL) was added dropwise, and the reaction mixture stirred for three hours at ambient temperature under N_2. The solvent was removed *in vacuo*. Purification by column chromatography (SiO_2; CH_2Cl_2/MeOH 99:1→95:5), preparative thin-layer chromatography (SiO_2; CH_2Cl_2/MeOH 99:1→95:5) and size-exclusion chromatography (Bio-Beads S-X1/ $CHCl_3$) gave the compound as a yellow solid (7.6 mg, 3 %, 3.7 µmol). ^{1}H NMR (500 MHz, $CDCl_3$/CD_3OD 9:1) 9.58 (1H, s, PyH), 9.32 (2H, d, $^{3}J_{HH}$ = 5.8, ArH), 9.13 (1H, s, ArH), 8.75 (2H, s, PyH), 8.04 (1H, s, ArH), 7.79 (2H, d, $^{3}J_{HH}$ = 8.3, ArH), 7.67 (6H, app. dd, J_{app} = 20.6, 8.3, ArH + StH), 7.51 – 7.46 (1H, m, ArH), 7.39 (1H, s, ArH), 7.33 – 7.07 (20H, m, ArH + StH), 7.04 (8H, d, $^{3}J_{HH}$ = 8.5, StH), 6.64 (4H, d, $^{3}J_{HH}$ = 8.5, HQH), 6.28 (4H, d, $^{3}J_{HH}$ = 8.6, HQH), 4.09 (4H, s, OCH_2), 3.92 (4H, s, OCH_2), 3.75 (4H, s, OCH_2), 3.65 – 3.56 (8H, m, OCH_2), 3.48 – 3.42 (4H, m, NCH_2), 3.30 (3H, s, NCH_3), 1.22 (36H, s, $C(CH_3)_3$); ^{13}C NMR (126 MHz, $CDCl_3$/CD_3OD 9:1) δ_c 169.1, 164.7, 158.7, 152.9, 151.9, 148.7, 146.9, 144.8, 143.5, 139.3, 134.9, 133.9, 133.6, 131.9, 131.0, 130.6, 128.8, 127.4, 125.9, 124.3, 123.6, 123.1, 119.6, 115.5, 115.0, 70.7, 70.6, 70.2, 68.3, 67.5, 66.2, 63.9, 57.8, 40.2, 38.7, 34.3, 31.3, 30.4, 29.7, 28.9, 23.7, 23.0, 22.7, 17.9, 14.0, 10.9; MALDI-TOF MS m/z calcd. for $[C_{116}H_{121}Cl_2N_7O_{11}Pt - Cl]^+$ 2018.32, found 2018.89.

^{1}H NMR spectrum (500 MHz, $CDCl_3/CD_3OD$ 9:1, 298 K)

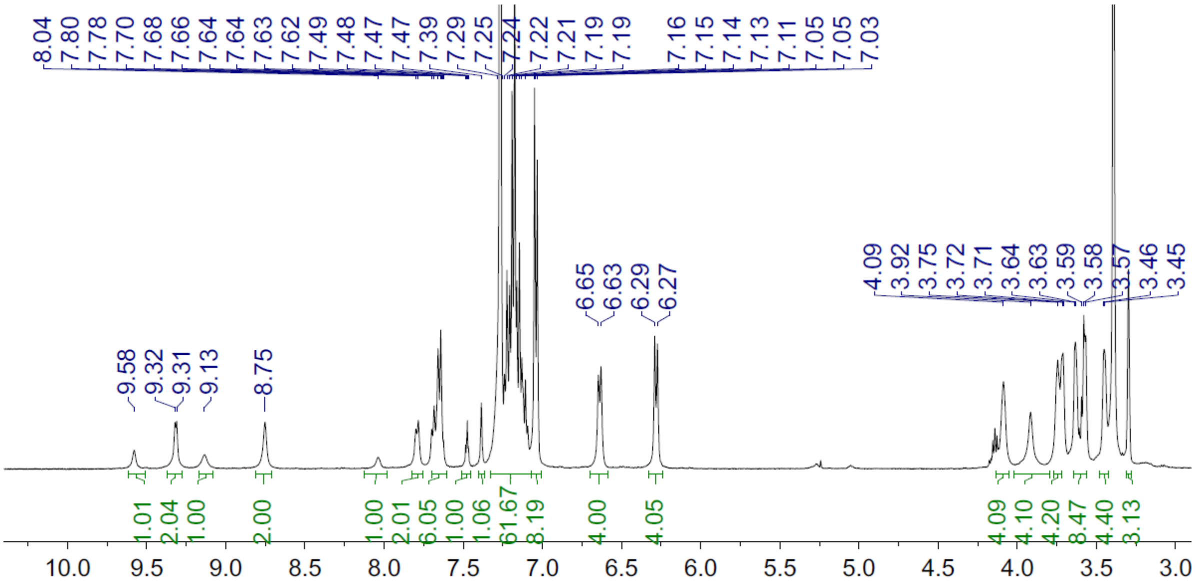

^{13}C NMR spectrum (126 MHz, $CDCl_3/CD_3OD$ 9:1, 298 K)

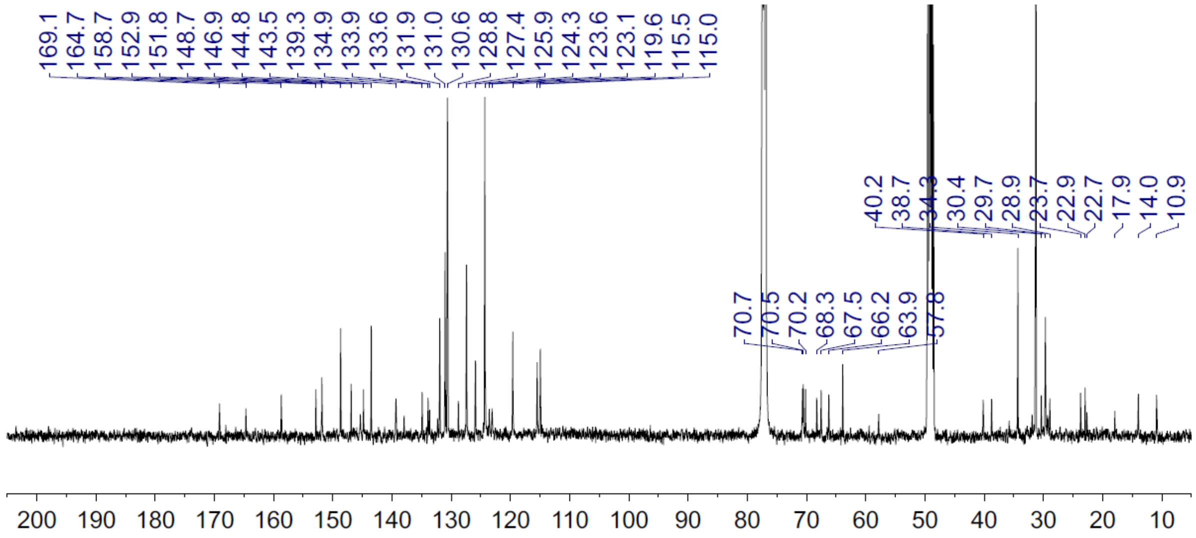

^{1}H-^{1}H ROESY 2D Correlation NMR spectrum (500 MHz, CD_2Cl_2, 298 K)

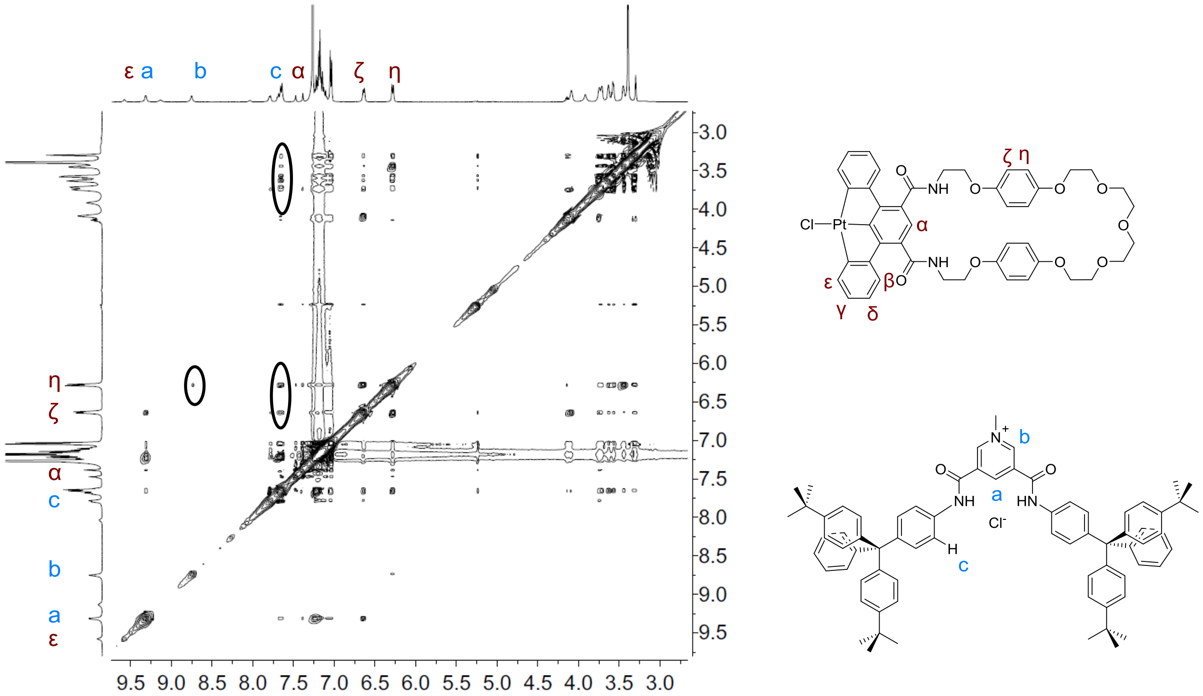

HRMS spectrum (ESI)

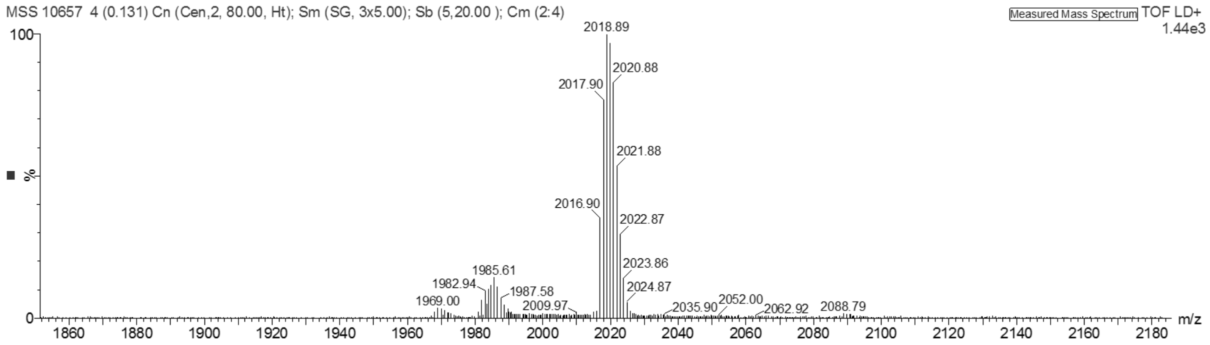

Synthesis of [2]rotaxane (2,4-di-2-pyridyl-isophthalamide)(4-tolylterpyridine) ruthenium(II) hexafluorophosphate ([Ru^{II}(dpb-macrocycle)(ttpy)PF_6] [2]rotaxane) 12·Ru·$(PF_6)_2$

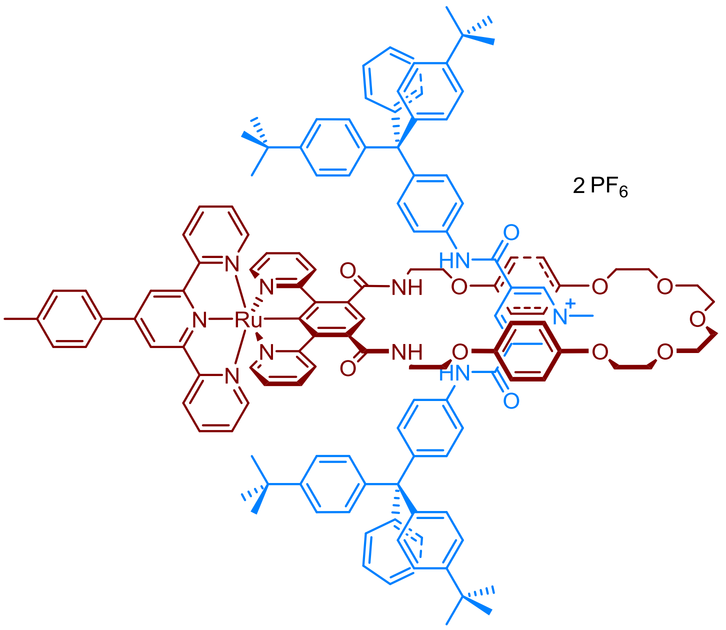

Oxalyl chloride (0.08 mL, 0.1 mmol) was added dropwise to a solution of [Ru^{II}(dpb-acid)(ttpy)PF_6] **6·Ru·PF$_6$** (0.023 g, 0.025 mmol) in anhydrous CH_2Cl_2 (10 mL) and anhydrous degassed catalytic DMF (1 drop). The reaction mixture was stirred under N_2 at 40°C until homogenous (*ca.* 6 hours). The solvent was removed *in vacuo* and dried under high vacuum. Bis-amine **8** (0.012 g, 0.025 mmol), pyridinium axle **11·Cl** (0.027 g, 0.025 mmol) and anhydrous, distilled Et_3N (0.008 mL, 0.0625 mmol) were dissolved in anhydrous CH_2Cl_2 (10 mL) and stirred until homogenous. The acid chloride in anhydrous CH_2Cl_2 (10 mL) was added dropwise, and the reaction mixture stirred for three hours at ambient temperature under N_2. The solvent was removed *in vacuo*. Purification by column chromatography (SiO_2; CH_2Cl_2/MeOH; 95:5) and by preparative thin-layer chromatography (SiO_2; EtOAc/MeOH; 100:0→98:2). This was dissolved in CH_2Cl_2 (15 mL) and washed with 0.1 M $NH_4PF_{6(aq)}$ (10 × 10 mL) and H_2O (2 × 10 mL). The organic layer was dried over $MgSO_4$, and the solvent removed *in vacuo* to give the compound as a purple solid (4.9 mg, 6% over two-steps, 1.95 µmol); ^{1}H NMR (500 MHz, CD_2Cl_2) δ_H 9.47 (1H, br.s, ArH), 9.37 (1H, br.s, ArH),8.78 (2H, s, ArH), 8.67 (1H, s, ArH + StH), 8.32 (4H, d, $^3J_{HH}$ = 8.2, ArH + StH), 8.00 (4H, d, $^3J_{HH}$ = 7.9, ArH + StH), 7.79 (4H, s, ArH), 7.55 (5H, app. dd, J_{app} = 15.0, 8.2, ArH), 7.38 (4H, d, $^3J_{HH}$ = 8.5, StH), 7.30 (11H, d, $^3J_{HH}$ = 7.5, ArH + StH), 7.23 – 7.20 (9H, m, ArH + StH), 7.06 (4H, d, $^3J_{HH}$ = 5.9, StH), 6.79 (4H, d, $^3J_{HH}$ = 8.0, HQH), 6.62 (2H, t, $^3J_{HH}$ = 6.6, ArH), 6.33 (4H, d, $^3J_{HH}$ = 8.6, HQH), 4.10 (4H, s, OCH_2), 3.93 (4H, s, OCH_2), 3.77 (4H, s, OCH_2), 3.70 (4H, s, OCH_2), 3.63 (4H, s, OCH_2), 3.44 (4H, s, NCH_2), 2.53 (3H, s, $ArCH_3$), 1.24 (36H, s, $C(CH_3)_3$); ^{13}C NMR (126 MHz, $CDCl_3$/CD_3OD 9:1) δ_C 165.2, 165.1, 164.5, 164.4, 158.4, 158.3, 153.2, 152.2, 152.1, 148.7, 147.0, 146.0, 144.6, 144.5, 143.6, 143.5, 138.0, 136.6, 135.5, 134.8, 133.4, 131.8, 131.0, 130.6, 129.3, 128.9, 127.4, 127.3, 127.0, 125.8, 125.8, 124.4, 124.3, 123.8, 121.6, 120.6, 120.4, 114.9, 70.7, 70.6, 70.0, 68.3, 65.5, 63.8, 41.1, 41.0, 39.2, 34.3, 31.3, 29.7; ^{19}F NMR (472 MHz, CD_2Cl_2) δ_F -70.9 (d, ^1J = 742 Hz, PF_6); ^{31}P NMR (121 MHz, $CDCl_3$) δ_P -144.35 (sept., $^1J_{PF}$ = 714 Hz, PF_6); ESI-HRMS m/z calcd. for $[C_{138}H_{138}F_6N_{10}O_{11}PRu - 2PF_6]^{2+}$ 1106.4793, found 1106.4795; UV-Vis λ_{max}(CH_2Cl_2)/nm; ε/dm^3 mol^{-1} cm^{-1}: 251 (96,300) 289 (64,000) 371 (9,800) 516 (14,200) 557 (12,100).

^{1}H NMR spectrum (500 MHz, CD_2Cl_2, 298 K)

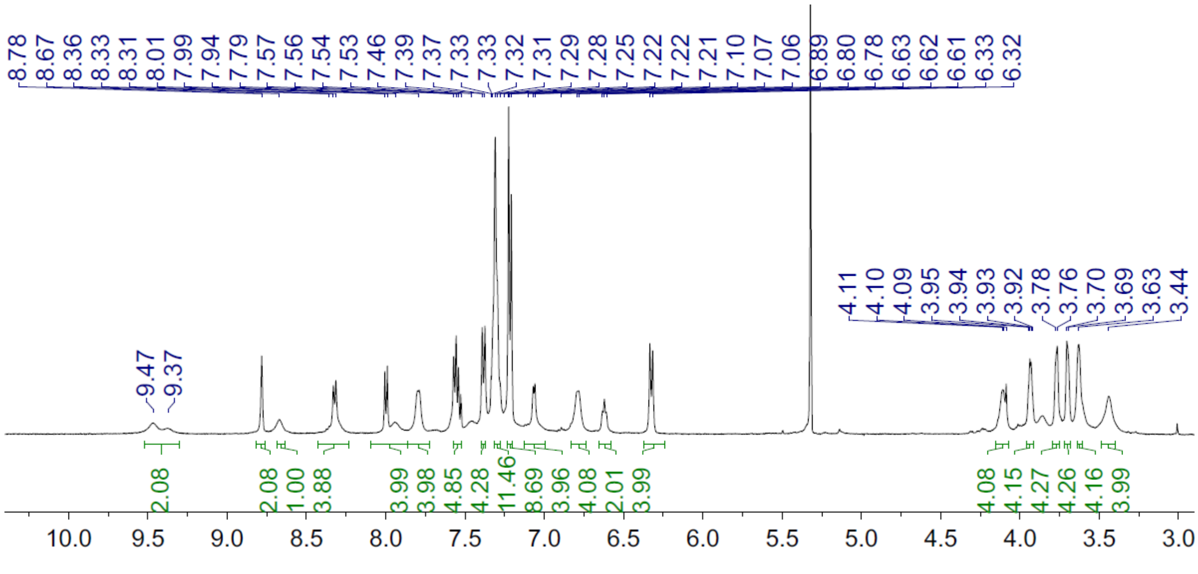

^{13}C NMR spectrum (126 MHz, $CDCl_3$/CD_3OD 9:1, 298 K)

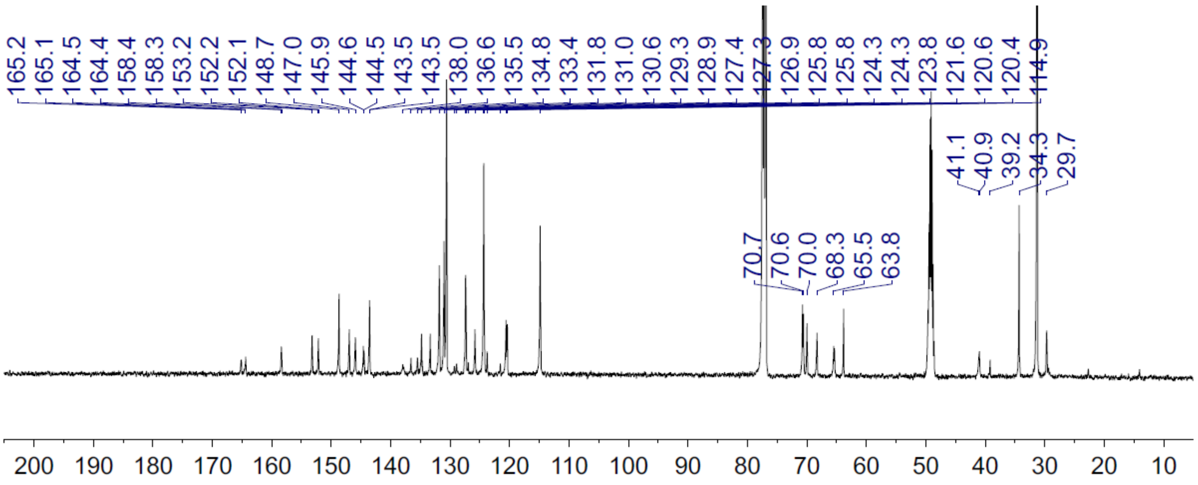

^{1}H-^{1}H ROESY 2D Correlation NMR spectrum (500 MHz, CD_2Cl_2, 298 K)

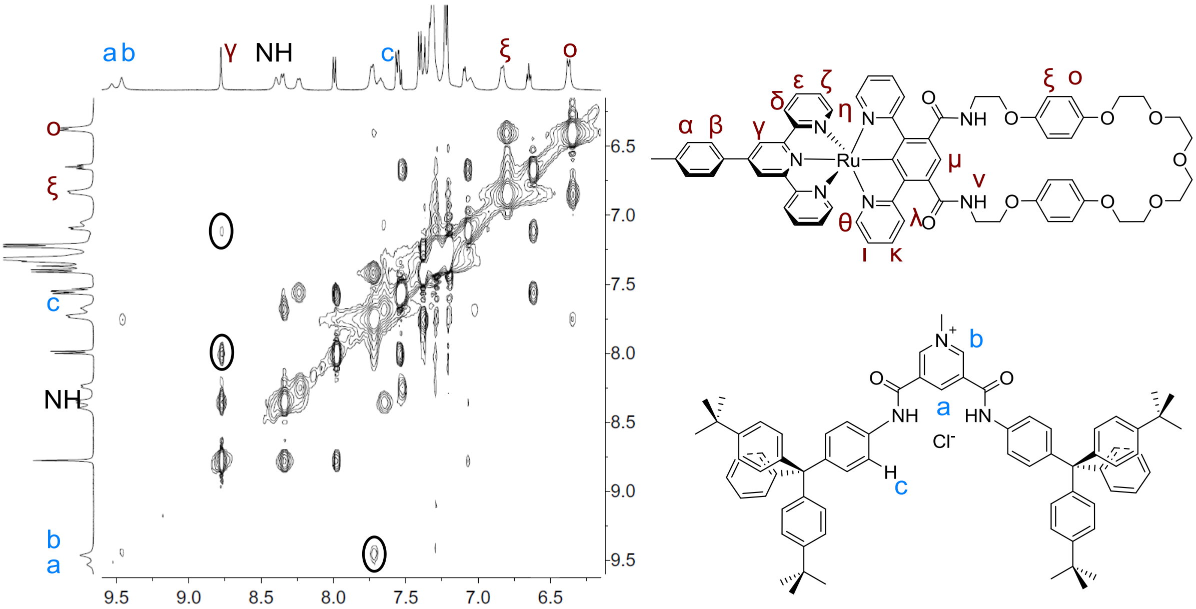

HRMS spectrum (ESI)

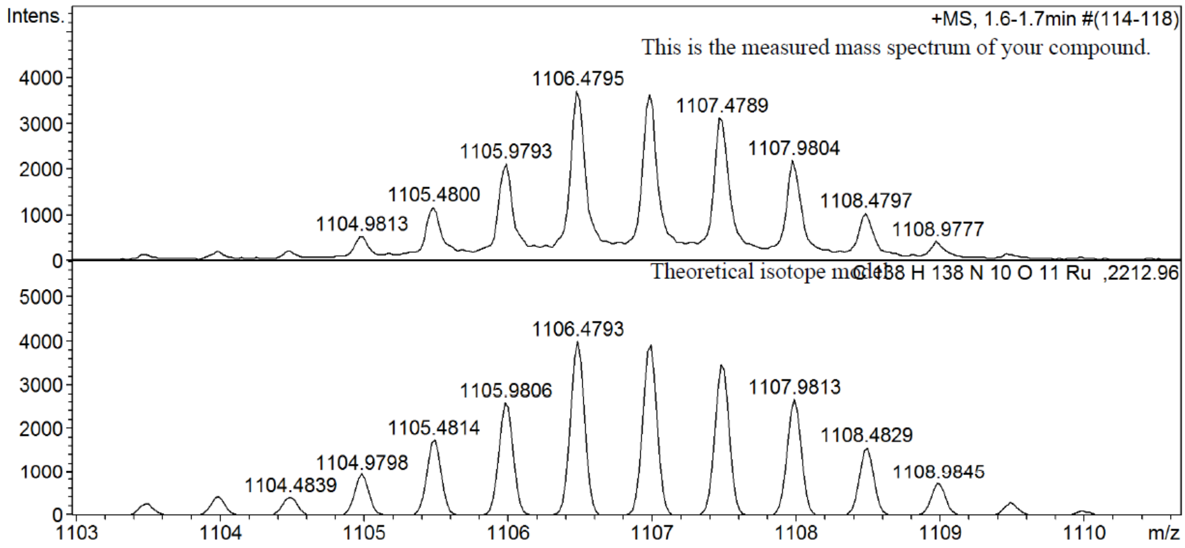

2. UV-vis and luminescence data

2.1 Absorption, Emission and Excitation profiles for novel receptors

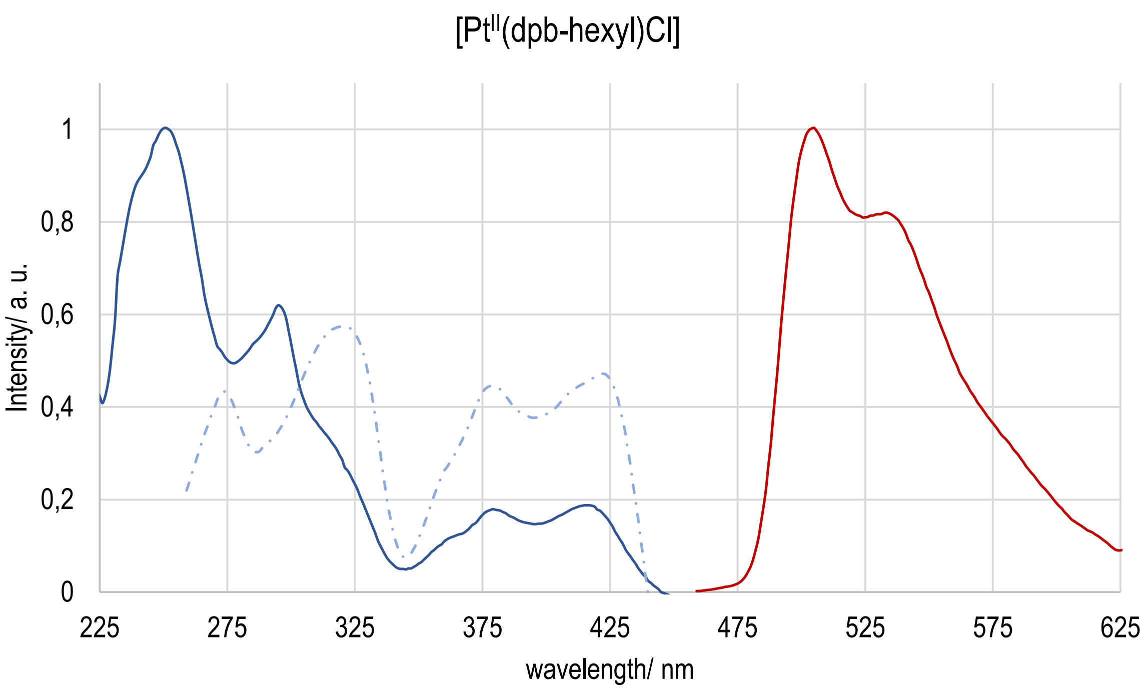

Photophysical properties of [Pt^{II}(dpb-hexyl)Cl] in CH_2Cl_2 (Absorption = blue, emission = red and excitation = dashed)

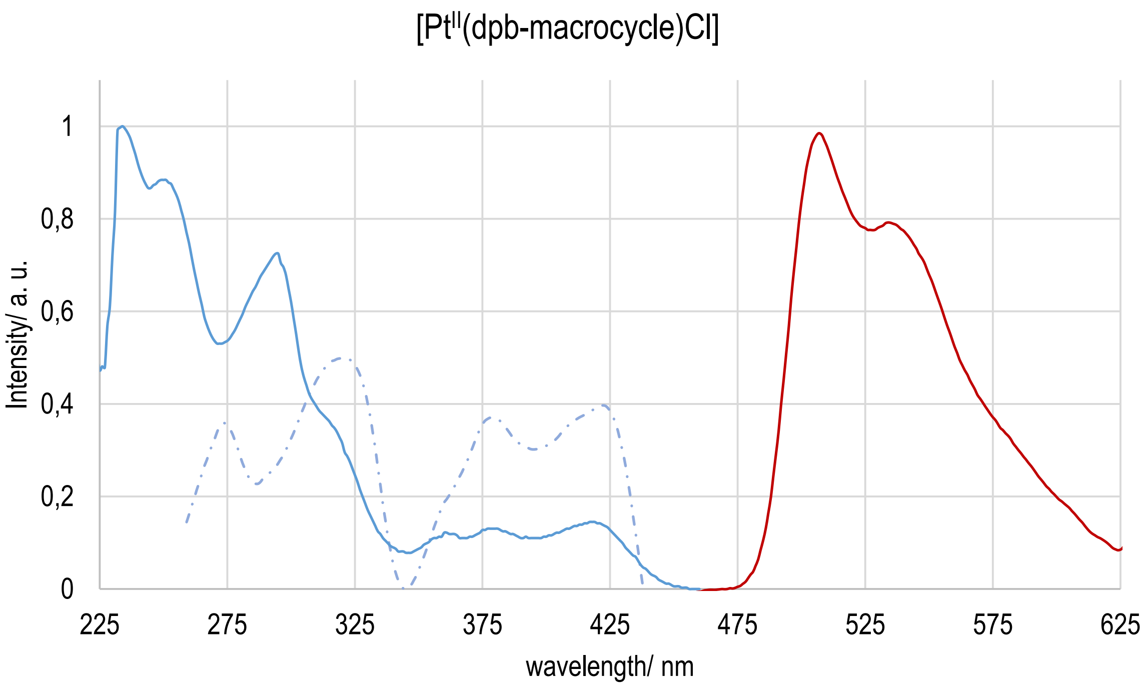

Photophysical properties of [Pt^{II}(dpb-macrocycle)Cl] in CH_2Cl_2 (Absorption = blue, emission = red and excitation = dashed)

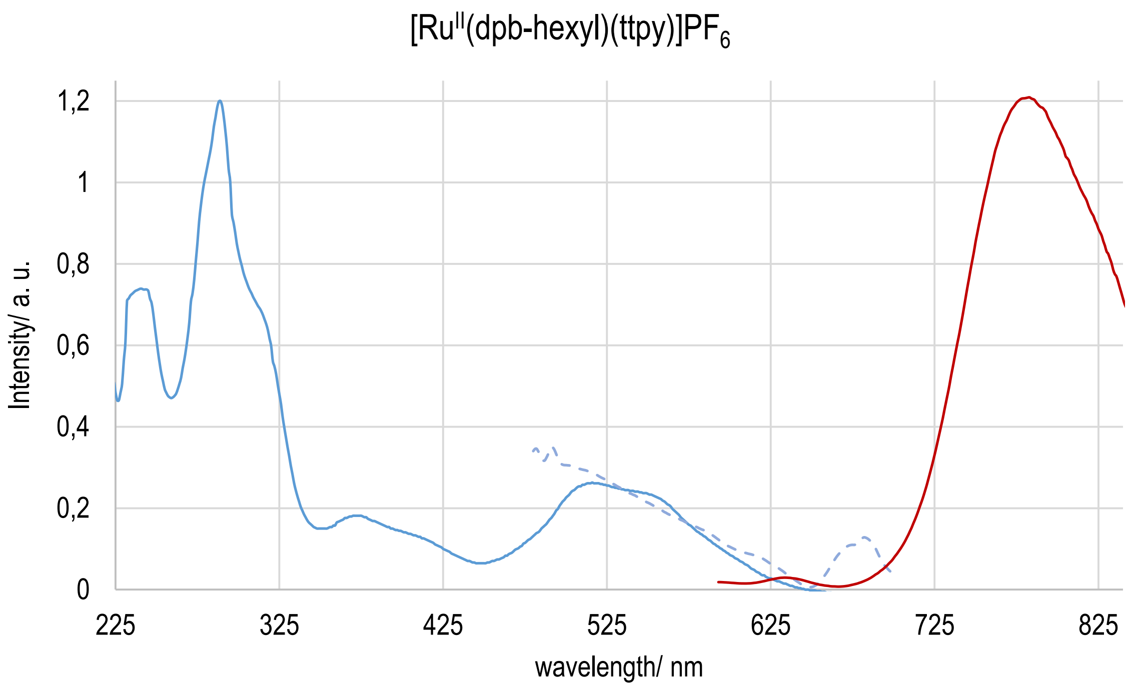

Photophysical properties of [Ru^{II}(dpb-hexyl)(ttpy)]PF_6 in CH_2Cl_2/MeOH 95:5 (Absorption = blue, emission = red and excitation = dashed)

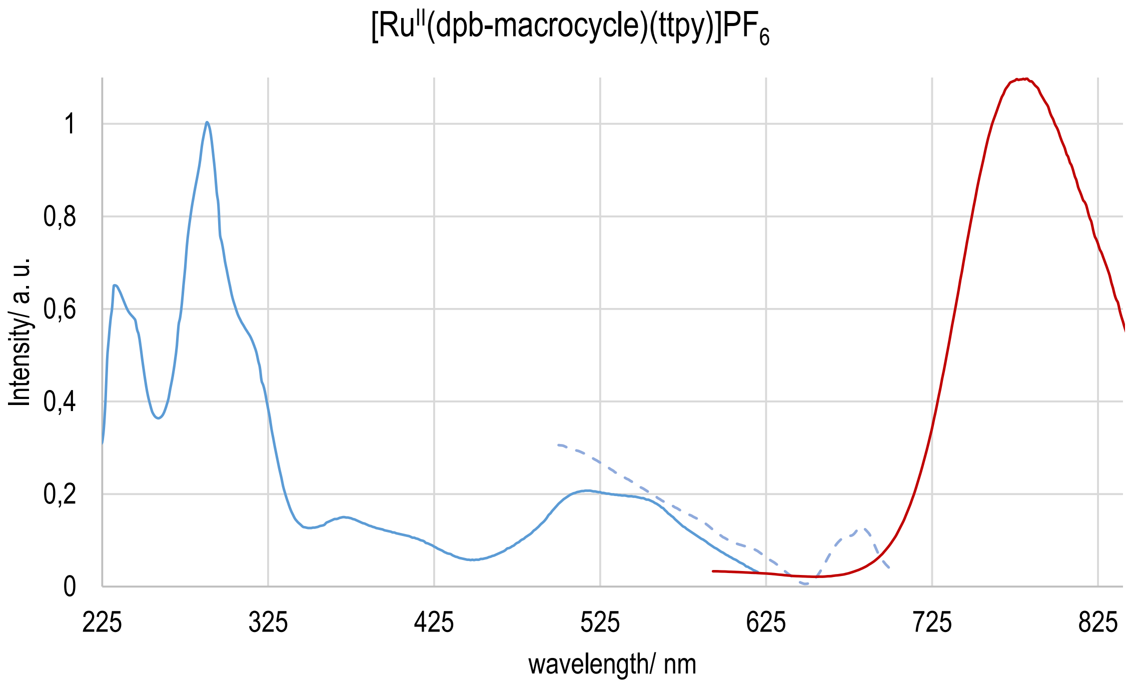

Photophysical properties of [Ru^{II}(dpb-macrocycle)(ttpy)]PF_6 in CH_2Cl_2/MeOH 95:5 (Absorption = blue, emission = red and excitation = dashed)

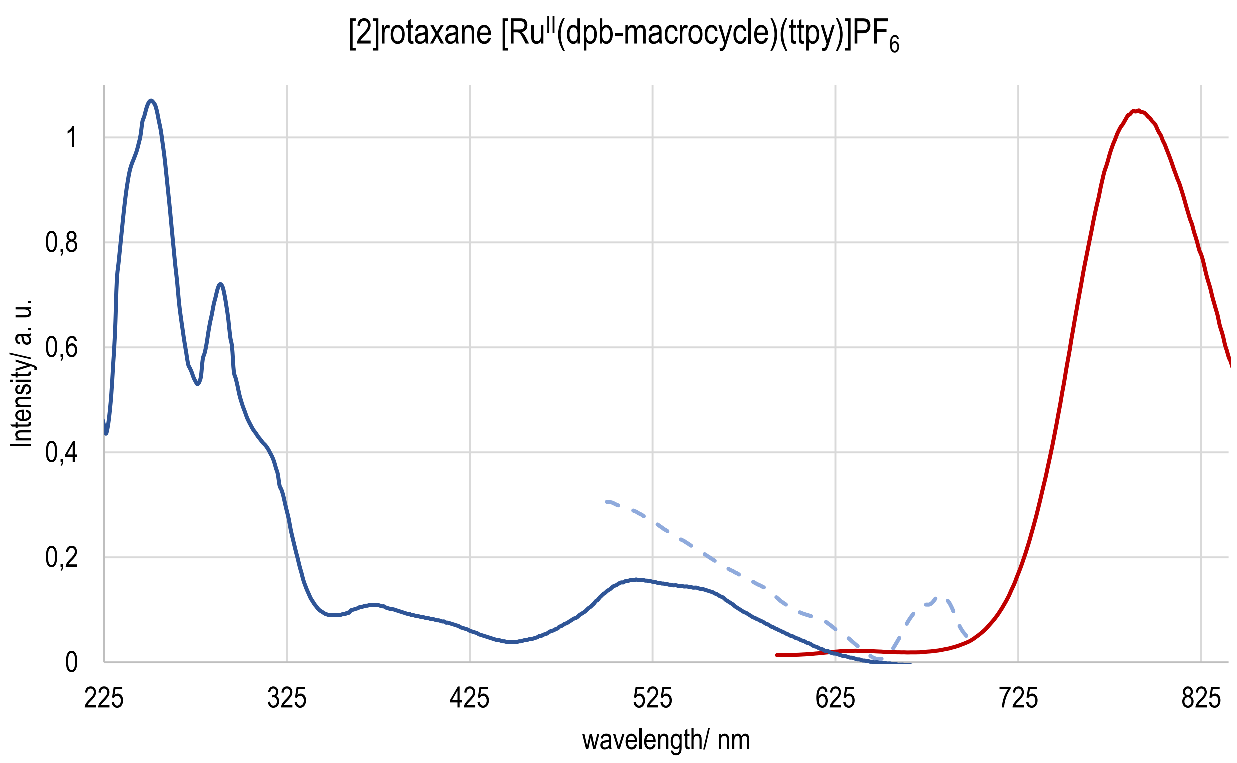

Photophysical properties of [Ru^{II}(dpb-macrocycle)(ttpy)]PF$_6$ in CH_2Cl_2/MeOH 95:5 (Absorption = blue, emission = red and excitation = dashed)

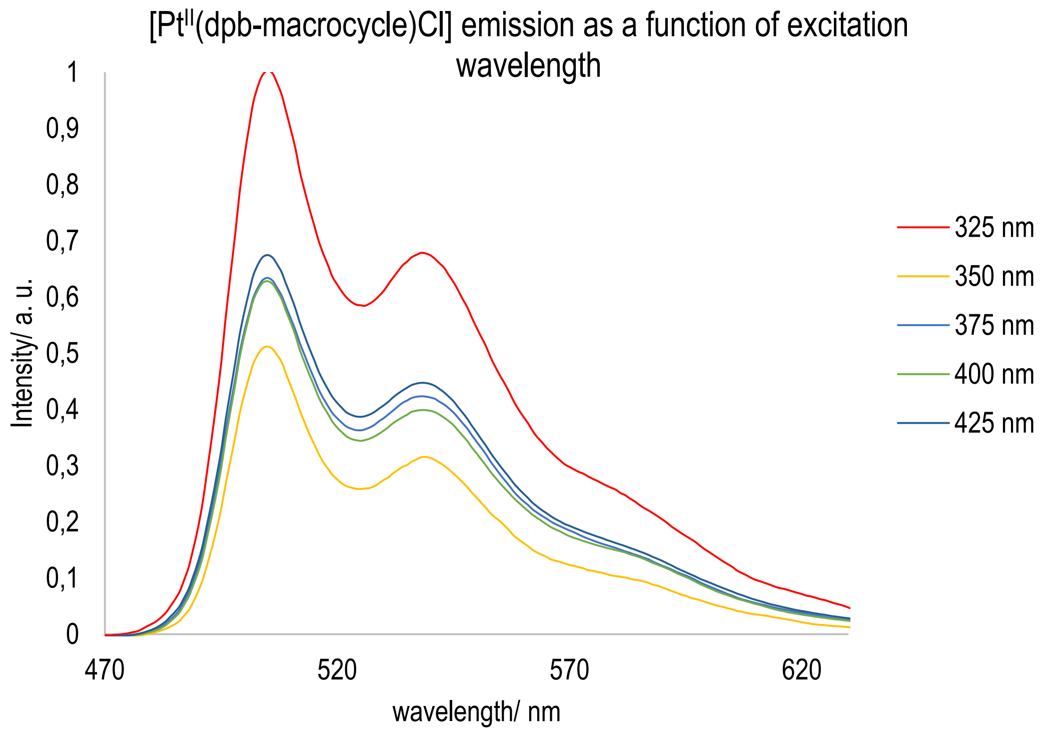

Emissive properties of [Pt^{II}(dpb-macroycle)Cl] in CH_2Cl_2 as a function of changing excitation wavelength

2.2 Luminescent Anion binding titrations

Luminescence experiments were carried out on a Varian Cary-Eclipse spectrometer for the platinum (II) receptors using an excitation wavelength of 320 nm at 293 K. To a 2.5 mL, 1 × 10^{-5} M solution of each receptor was added aliquots of the tetrabutylammonium salts dissolved in a stock solution made up with the receptor, such that the same concentration of the host was maintained throughout the titration experiments. Luminescence experiments were carried out on a Horiba Fluorolog spectrometer for the ruthenium (II) receptors using an excitation wavelength of 530 nm at 293 K. To a 1 mL, 1 × 10^{-4} M solution of each receptor was added aliquots of the tetrabutylammonium salts dissolved in a stock solution made up with the receptor, such that the same concentration of the host was maintained throughout the titration experiments. In both cases the titration data was analysed and association constants determined using the SPECFIT program.[10,11]

2.2.1 Anion titrations with [PtII(dpb-hexyl)Cl] 7·Pt

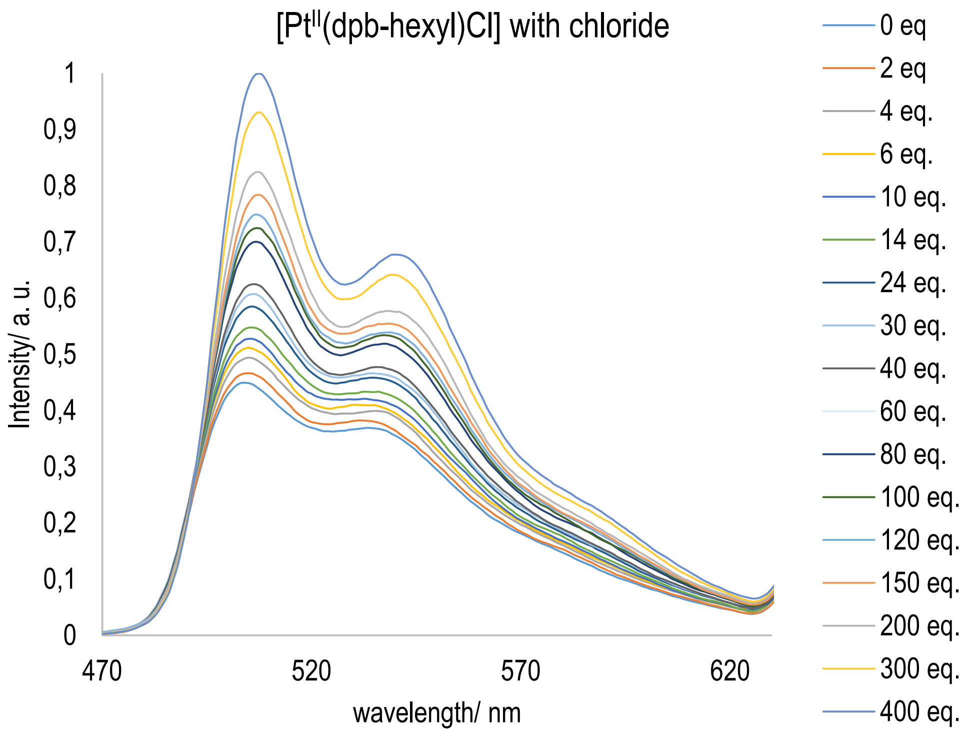

Emission spectra of [Pt^{II}(dpb-hexyl)Cl] in the presence of increasing amounts of chloride (CH_2Cl_2, 293 K; λ_{ex} = 320 nm)

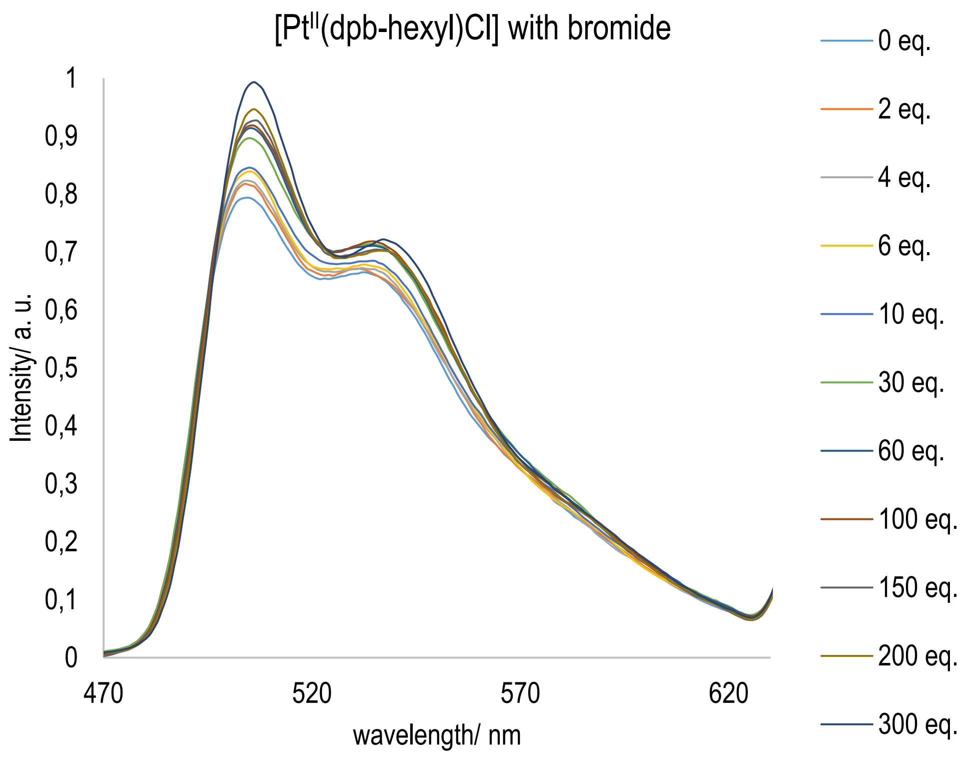

Emission spectra of [Pt^{II}(dpb-hexyl)Cl] in the presence of increasing amounts of bromide (CH_2Cl_2, 293 K; λ_{ex} = 320 nm)

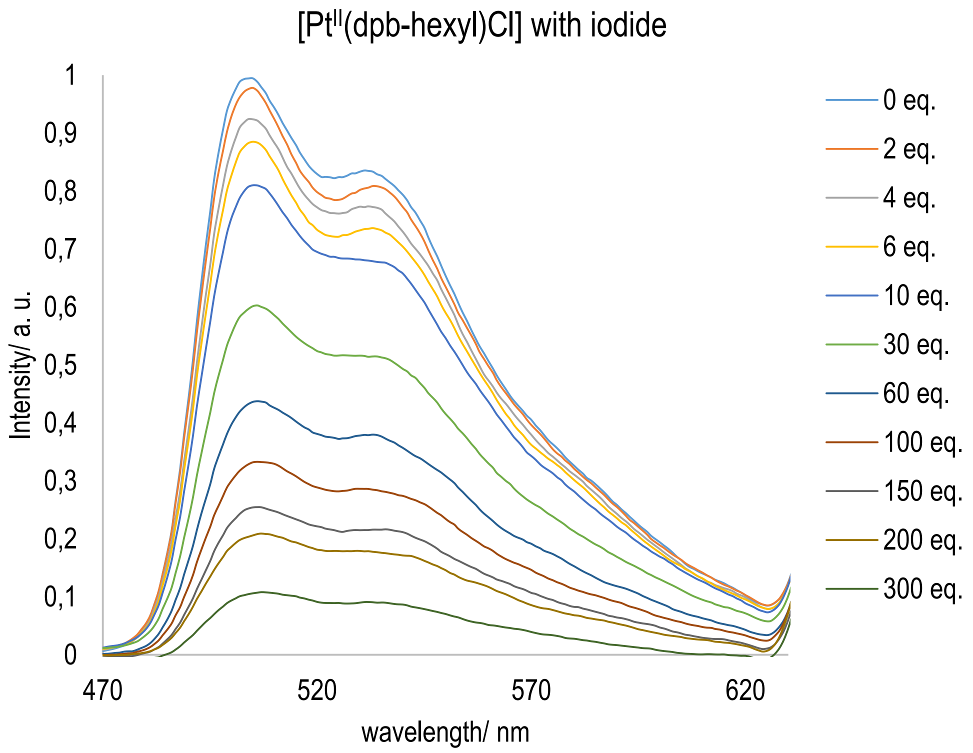

Emission spectra of [Pt^{II}(dpb-hexyl)Cl] in the presence of increasing amounts of iodide (CH_2Cl_2, 293 K; λ_{ex} = 320 nm)

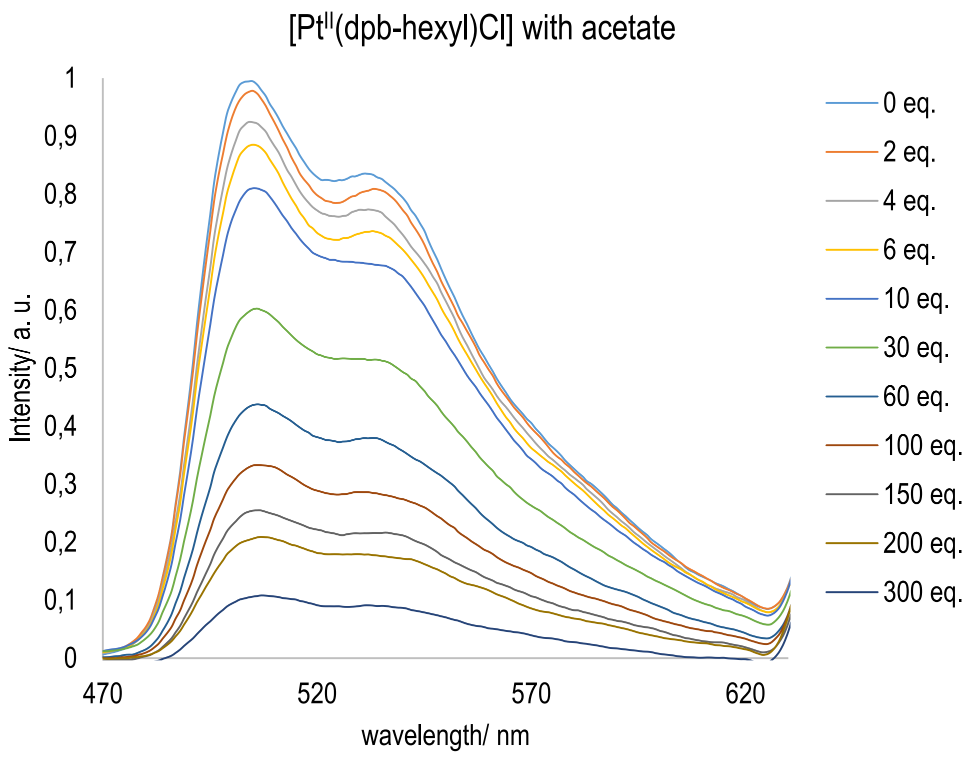

Emission spectra of [Pt^{II}(dpb-hexyl)Cl] in the presence of increasing amounts of acetate (CH_2Cl_2, 293 K; λ_{ex} = 320 nm)

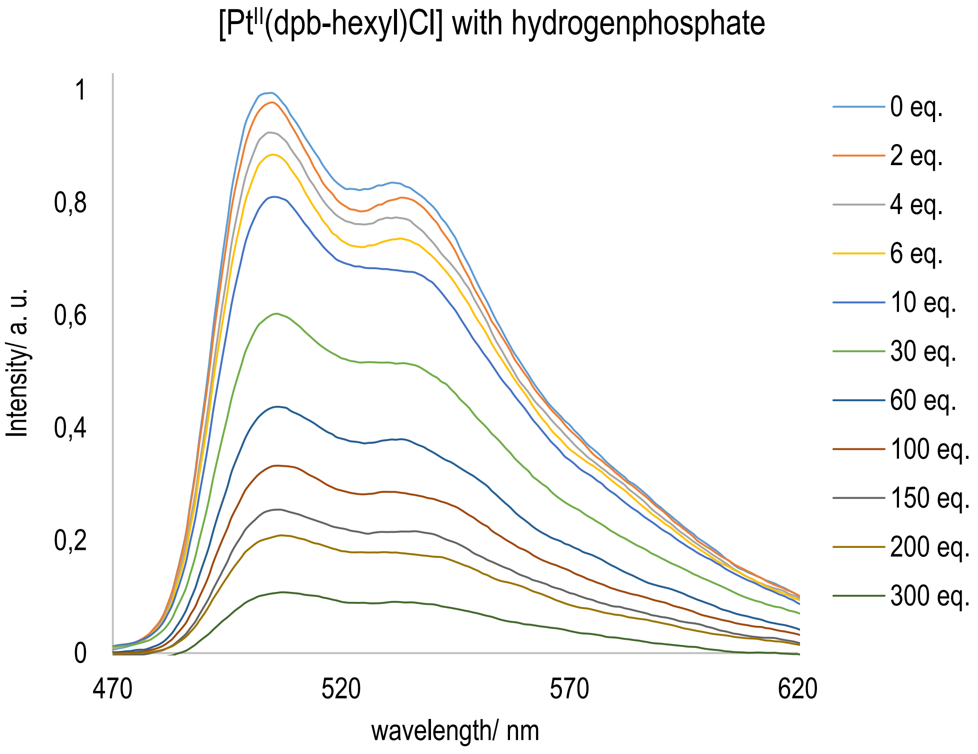

Emission spectra of [Pt^{II}(dpb-hexyl)Cl] in the presence of increasing amounts of dihydrogenphosphate (CH_2Cl_2, 293 K; λ_{ex} = 320 nm)

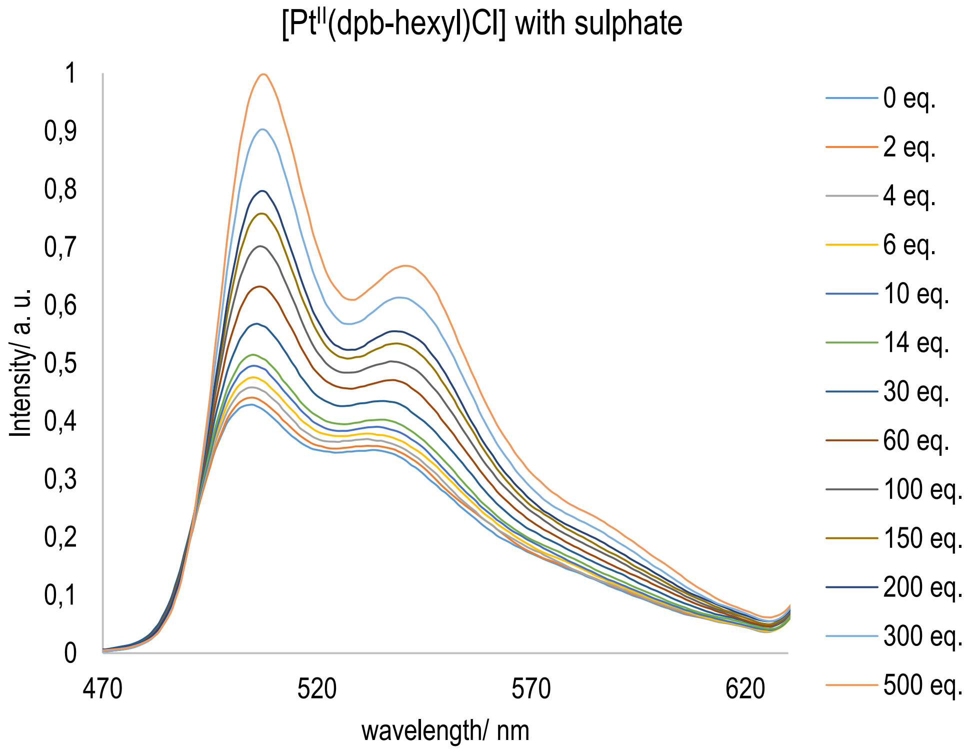

Emission spectra of [Pt^{II}(dpb-hexyl)Cl] in the presence of increasing amounts of sulphate (CH_2Cl_2, 293 K; λ_{ex} = 320 nm)

2.2.2 Anion titrations with [PtII(dpb-macrocycle)Cl] 10·Pt

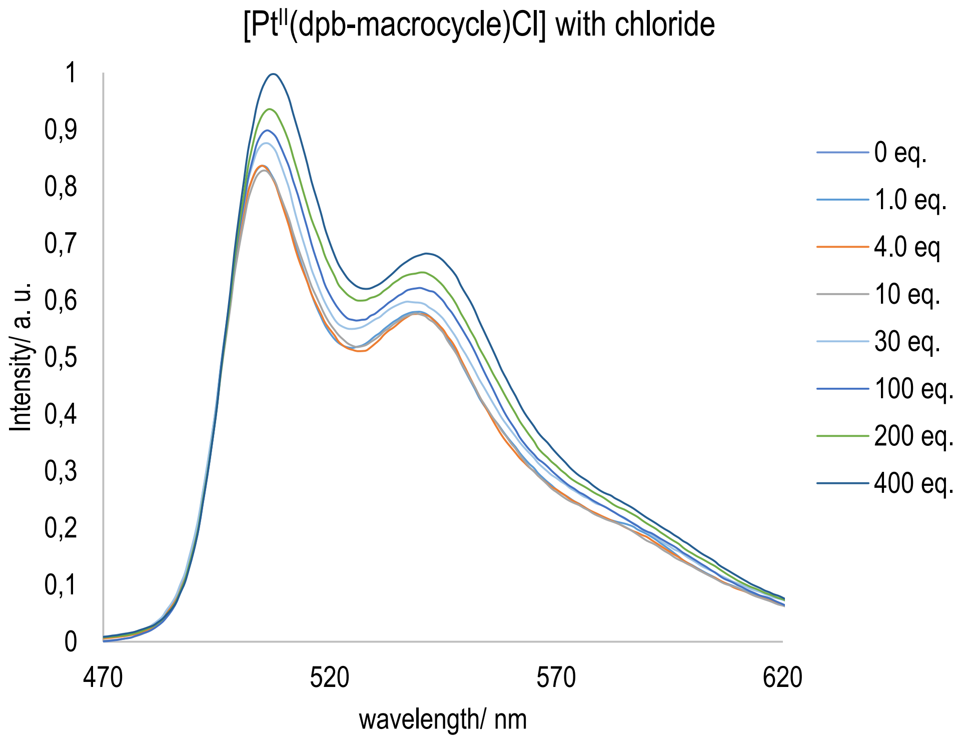

Emission spectra of [Pt^{II}(dpb-macrocycle)Cl] in the presence of increasing amounts of chloride (CH_2Cl_2, 293 K; λ_{ex} = 320 nm)

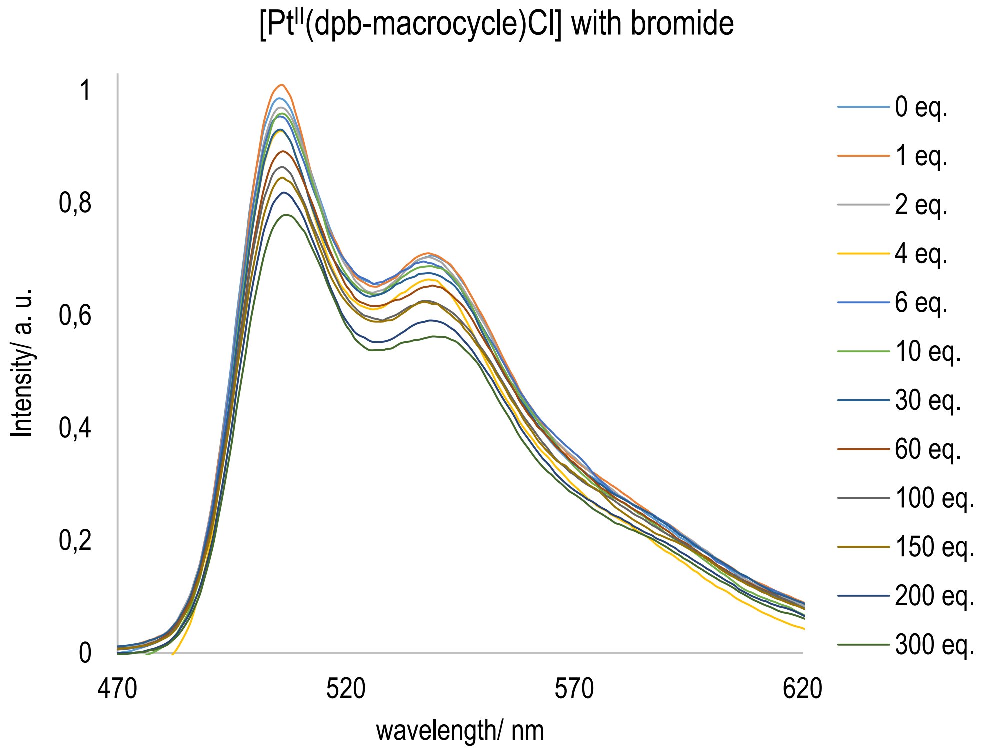

Emission spectra of [Pt^{II}(dpb-macrocycle)Cl] in the presence of increasing amounts of bromide (CH_2Cl_2, 293 K; λ_{ex} = 320 nm)

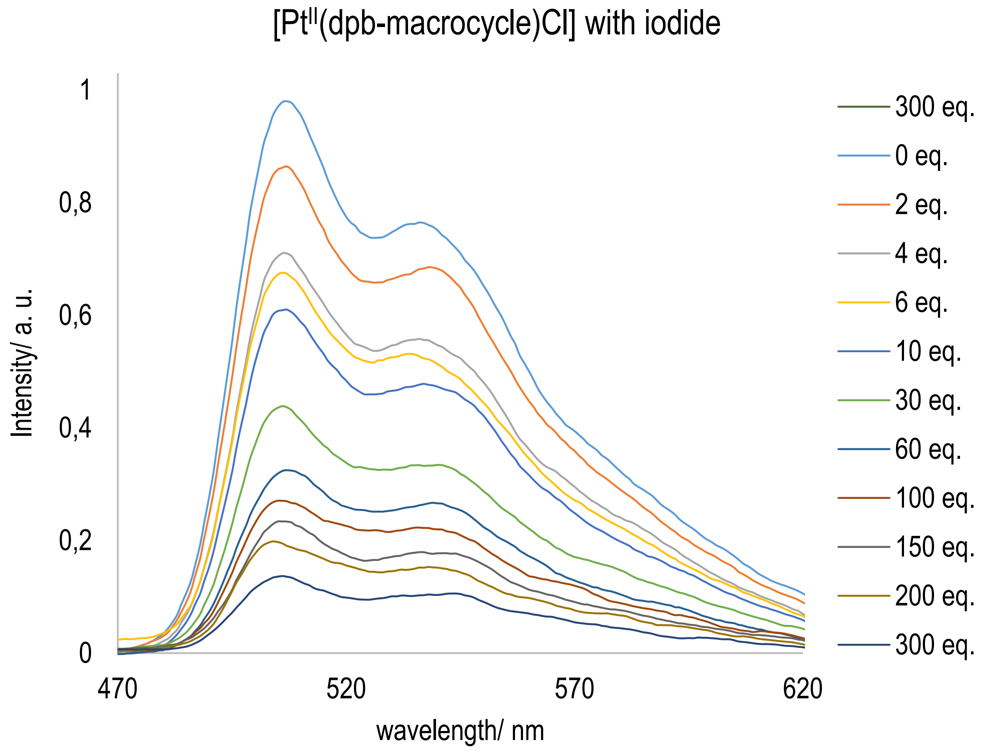

Emission spectra of [Pt^{II}(dpb-macrocycle)Cl] in the presence of increasing amounts of iodide (CH_2Cl_2, 293 K; λ_{ex} = 320 nm)

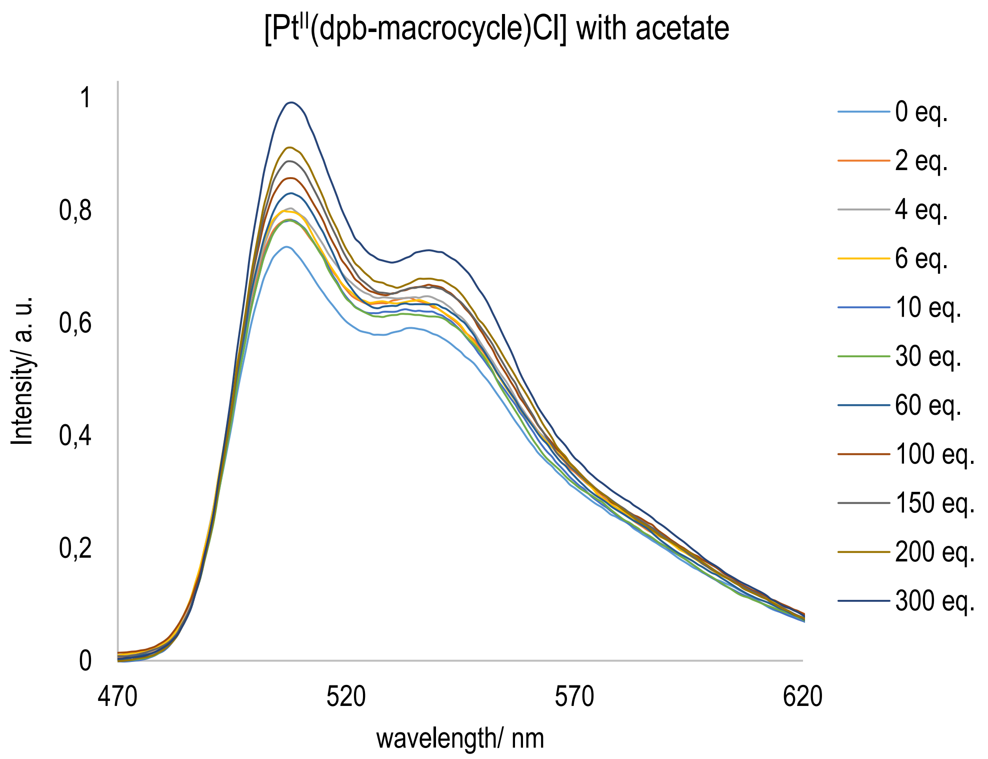

Emission spectra of [Pt^{II}(dpb-macrocycle)Cl] in the presence of increasing amounts of acetate (CH_2Cl_2, 293 K; λ_{ex} = 320 nm)

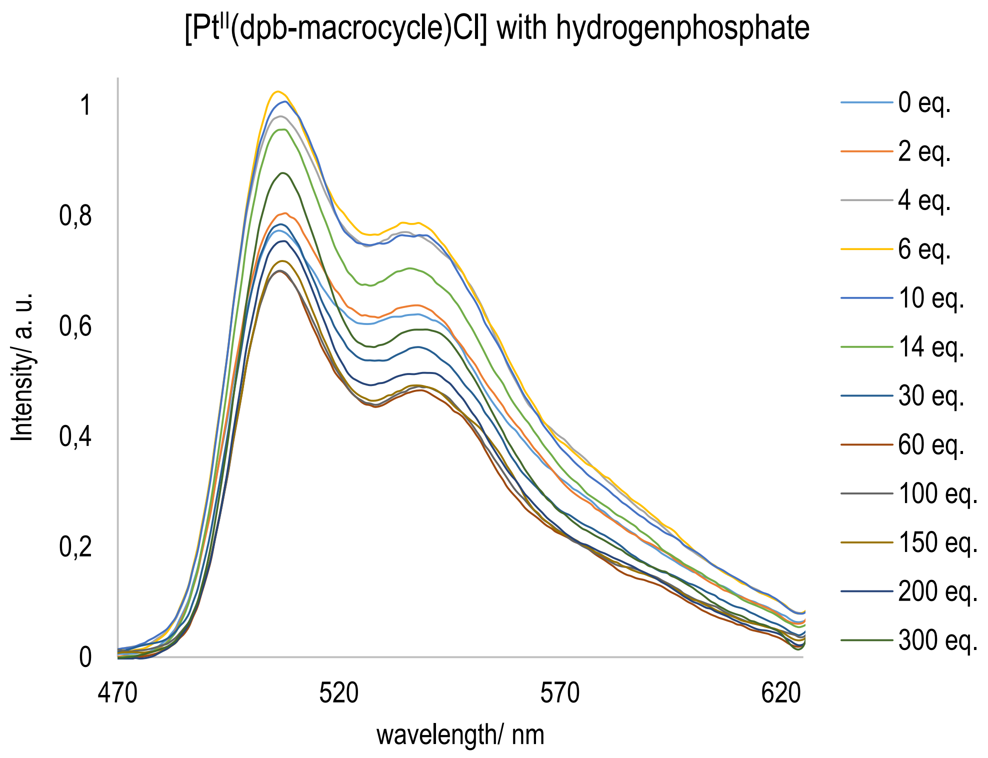

Emission spectra of [Pt^{II}(dpb-macrocycle)Cl] in the presence of increasing amounts of dihydrogenphosphate (CH_2Cl_2, 293 K; λ_{ex} = 320 nm)

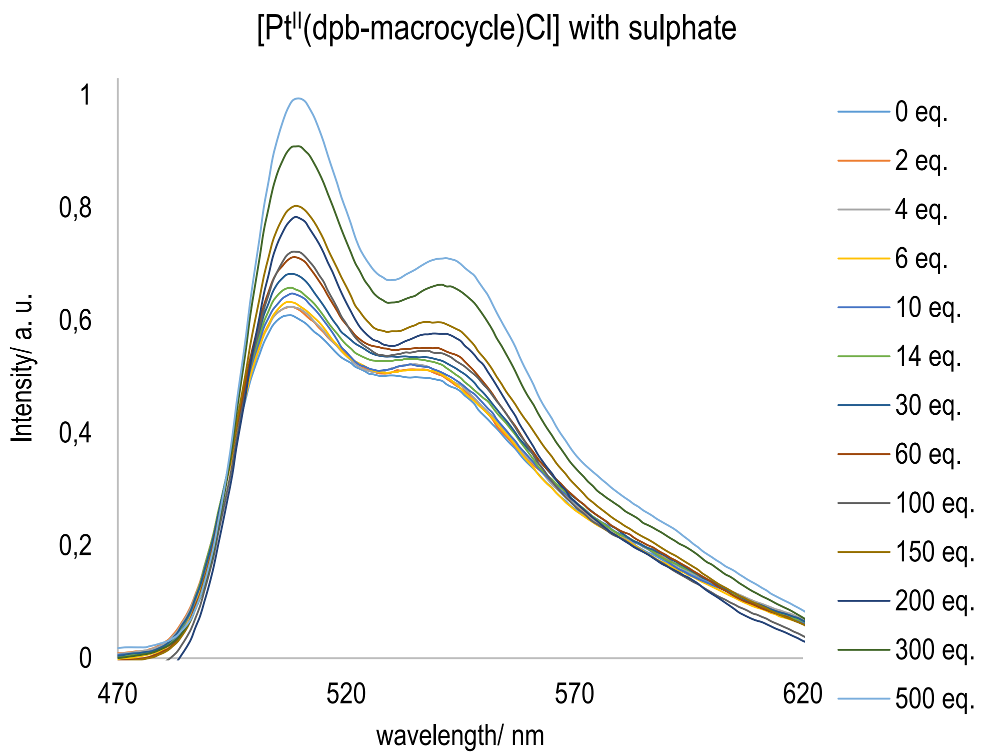

Emission spectra of [Pt^{II}(dpb-macrocycle)Cl] in the presence of increasing amounts of sulphate (CH_2Cl_2, 293 K; λ_{ex} = 320 nm)

2.2.3 Anion titrations with [RuII(dpb-hexyl)(ttpy)]PF6 7·Ru·PF6

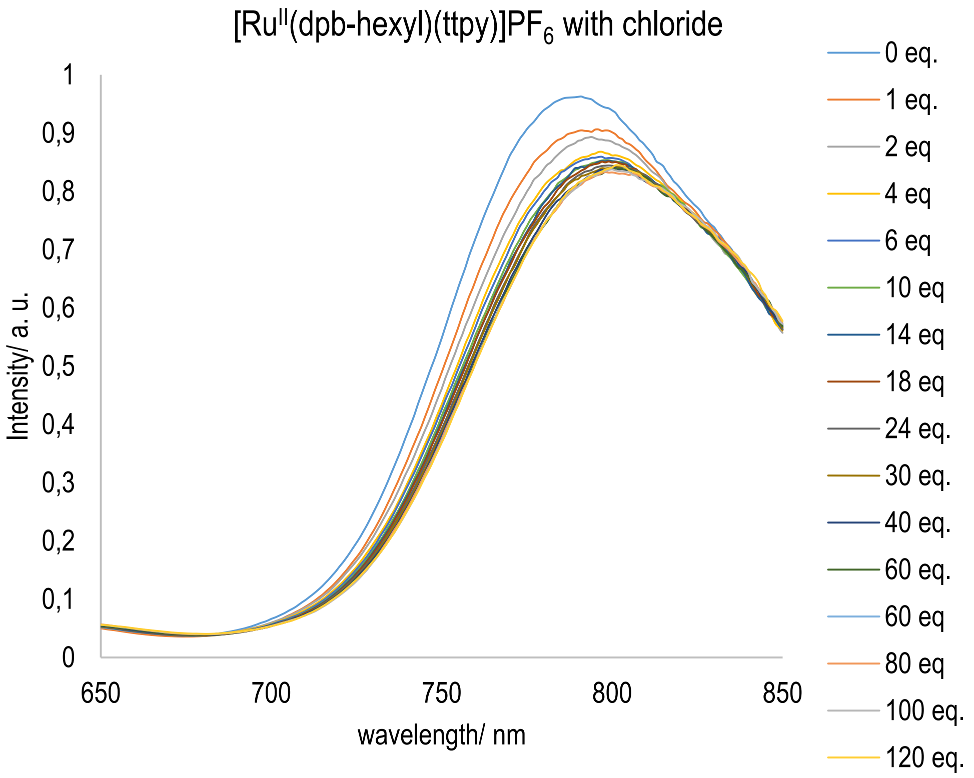

Emission spectra of [Ru^{II}(dpb-hexyl)(ttpy)]PF_6 in the presence of increasing amounts of chloride (CH_2Cl_2/MeOH 95:5 , 293 K; λ_{ex} = 530 nm)

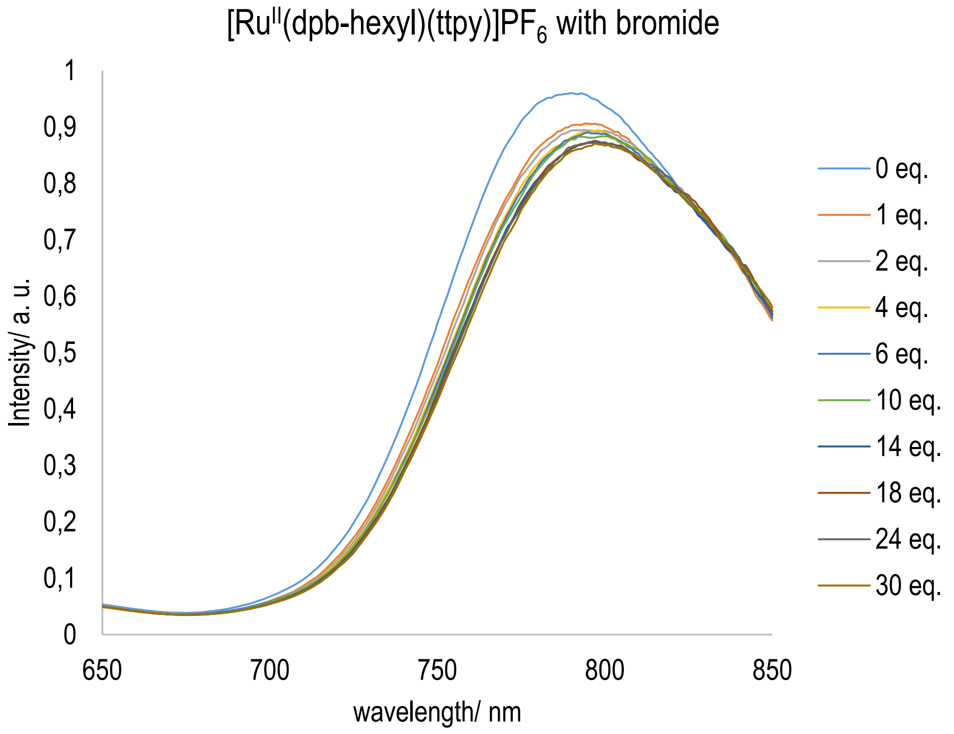

Emission spectra of $[Ru^{II}(dpb\text{-}hexyl)(ttpy)]PF_6$ in the presence of increasing amounts of bromide (CH_2Cl_2/MeOH 95:5 , 293 K; λ_{ex} = 530 nm)

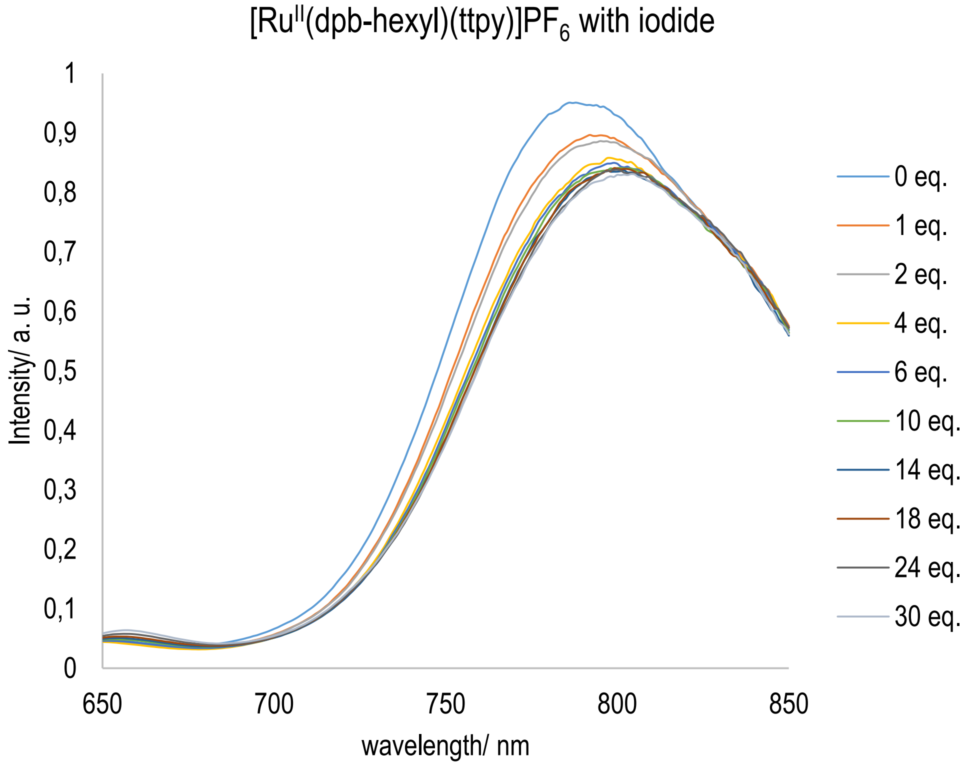

Emission spectra of $[Ru^{II}(dpb\text{-}hexyl)(ttpy)]PF_6$ in the presence of increasing amounts of iodide (CH_2Cl_2/MeOH 95:5 , 293 K; λ_{ex} = 530 nm)

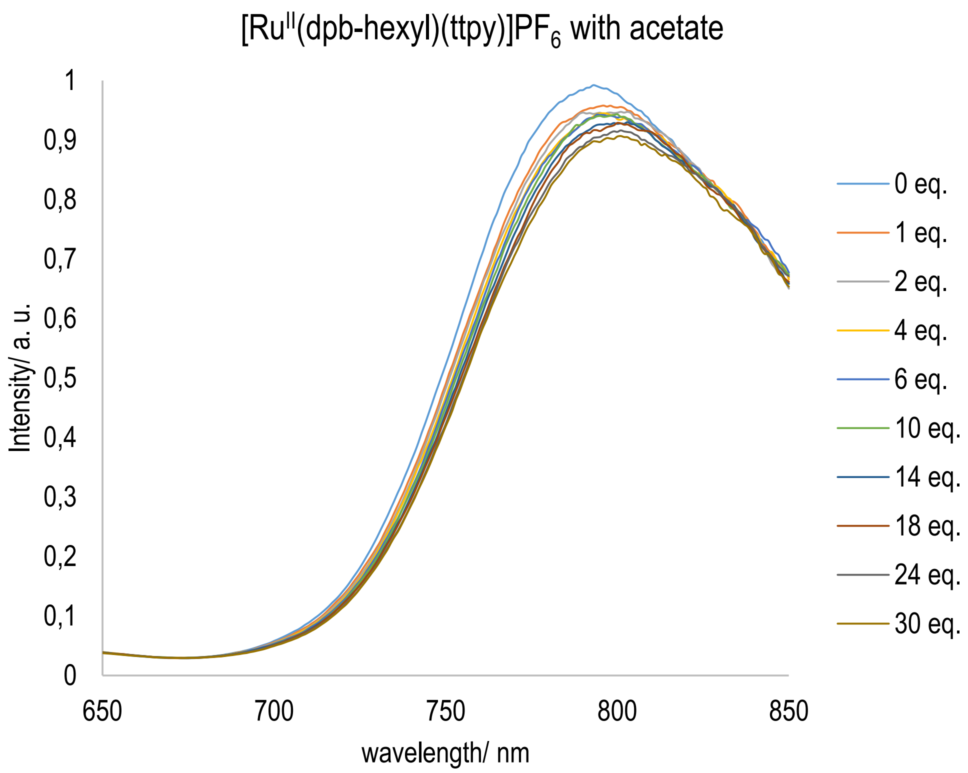

Emission spectra of [Ru^{II}(dpb-hexyl)(ttpy)]PF$_6$ in the presence of increasing amounts of acetate (CH_2Cl_2/MeOH 95:5 , 293 K; λ_{ex} = 530 nm)

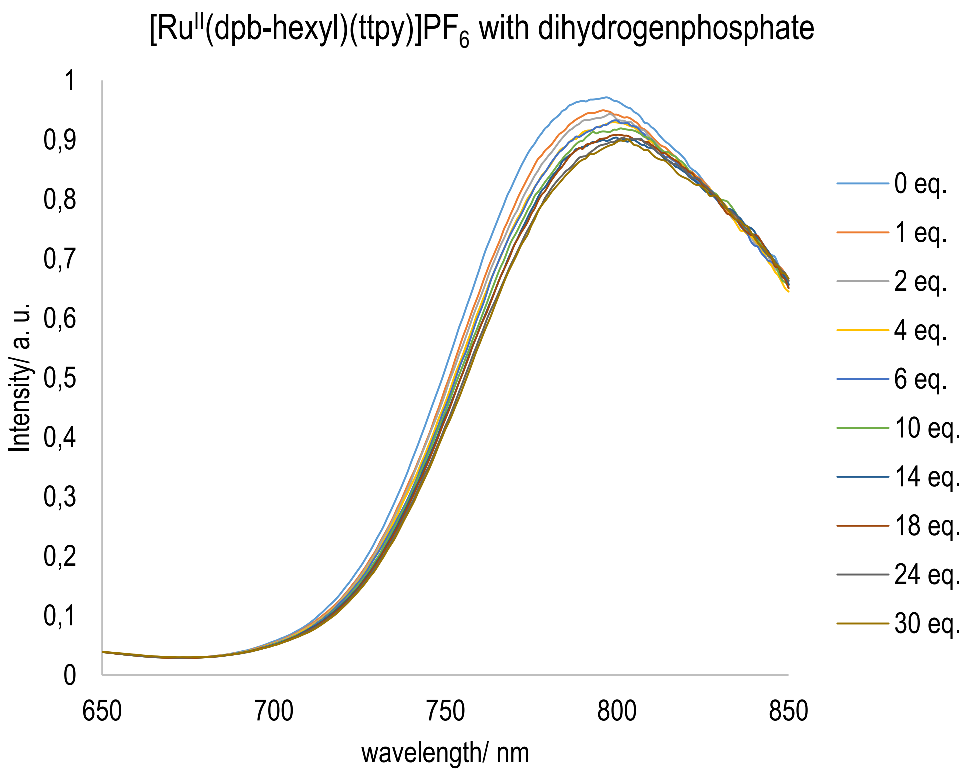

Emission spectra of [Ru^{II}(dpb-hexyl)(ttpy)]PF$_6$ in the presence of increasing amounts of dihydrogenphosphate (CH_2Cl_2/MeOH 95:5 , 293 K; λ_{ex} = 530 nm)

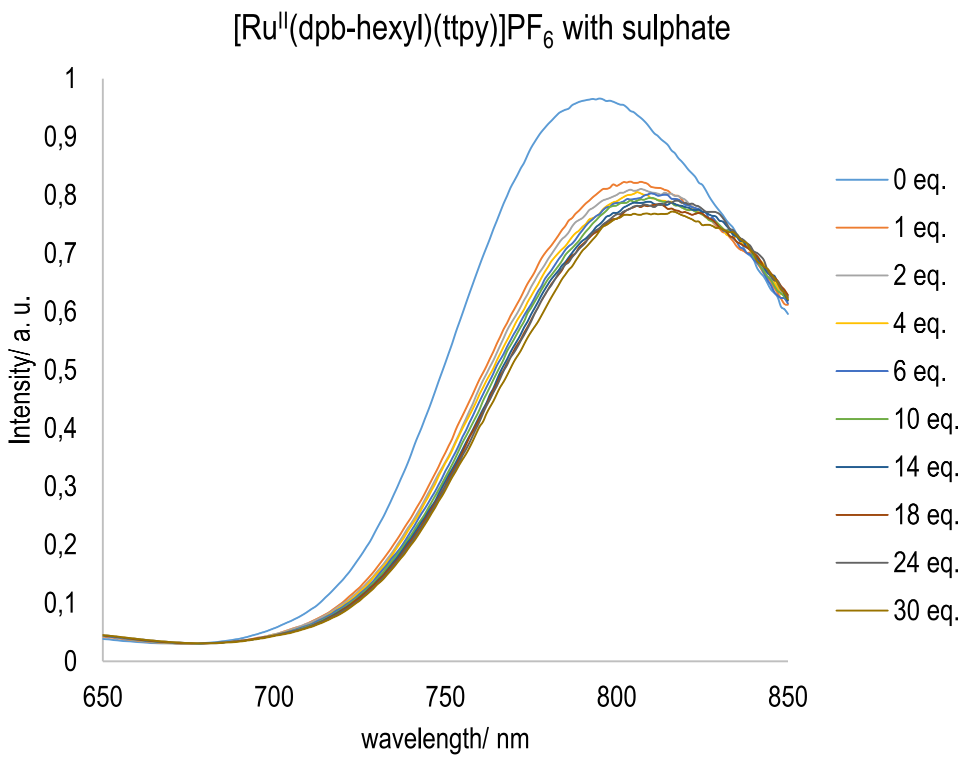

Emission spectra of $[Ru^{II}(dpb\text{-}hexyl)(ttpy)]PF_6$ in the presence of increasing amounts of sulphate

(CH_2Cl_2/MeOH 95:5 , 293 K; λ_{ex} = 530 nm)

2.2.4 Anion titrations with [RuII(dpb-macrocycle)(ttpy)]PF6 10·Ru·PF6

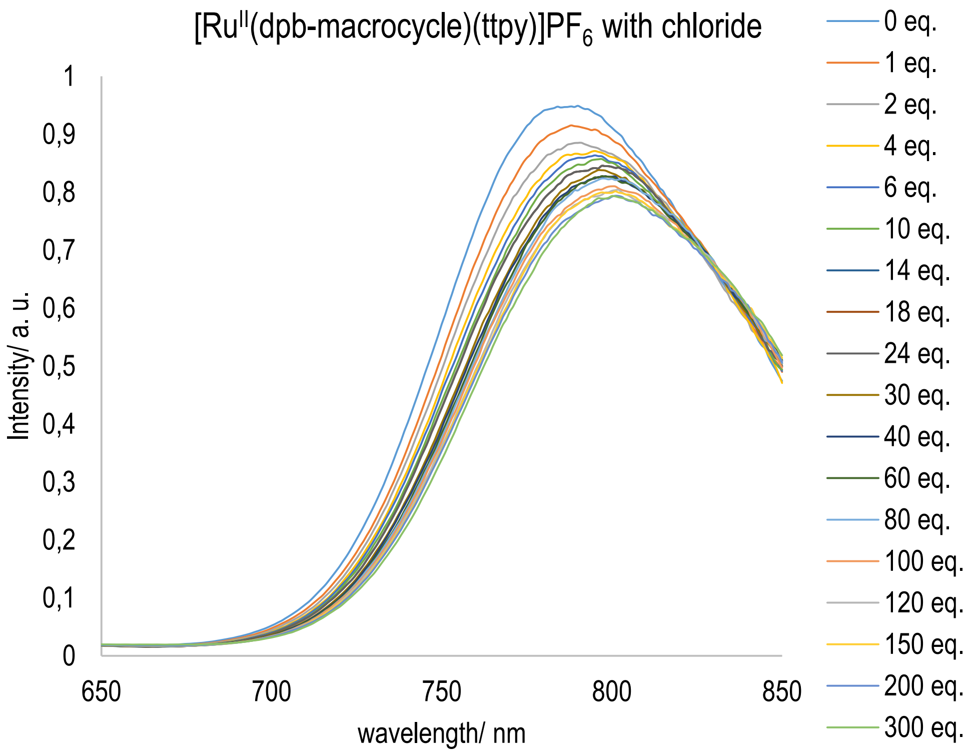

Emission spectra of $[Ru^{II}(dpb\text{-}macrocycle)(ttpy)]PF_6$ in the presence of increasing amounts of chloride

(CH_2Cl_2/MeOH 95:5 , 293 K; λ_{ex} = 530 nm)

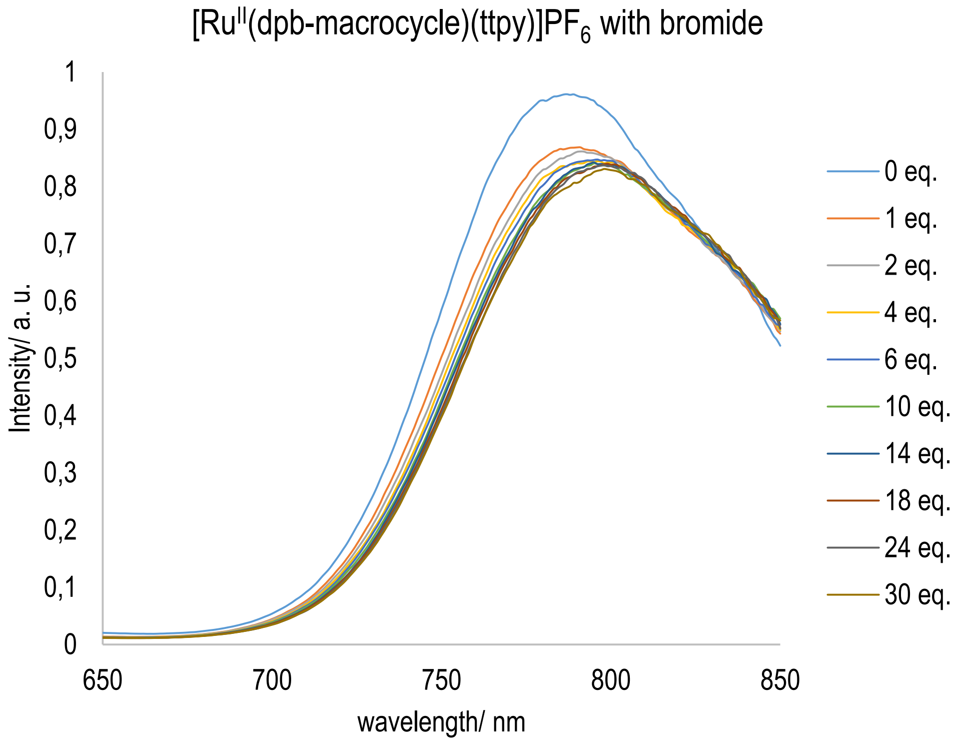

Emission spectra of [Ru^{II}(dpb-macrocycle)(ttpy)]PF_6 in the presence of increasing amounts of bromide (CH_2Cl_2/MeOH 95:5, 293 K; λ_{ex} = 530 nm)

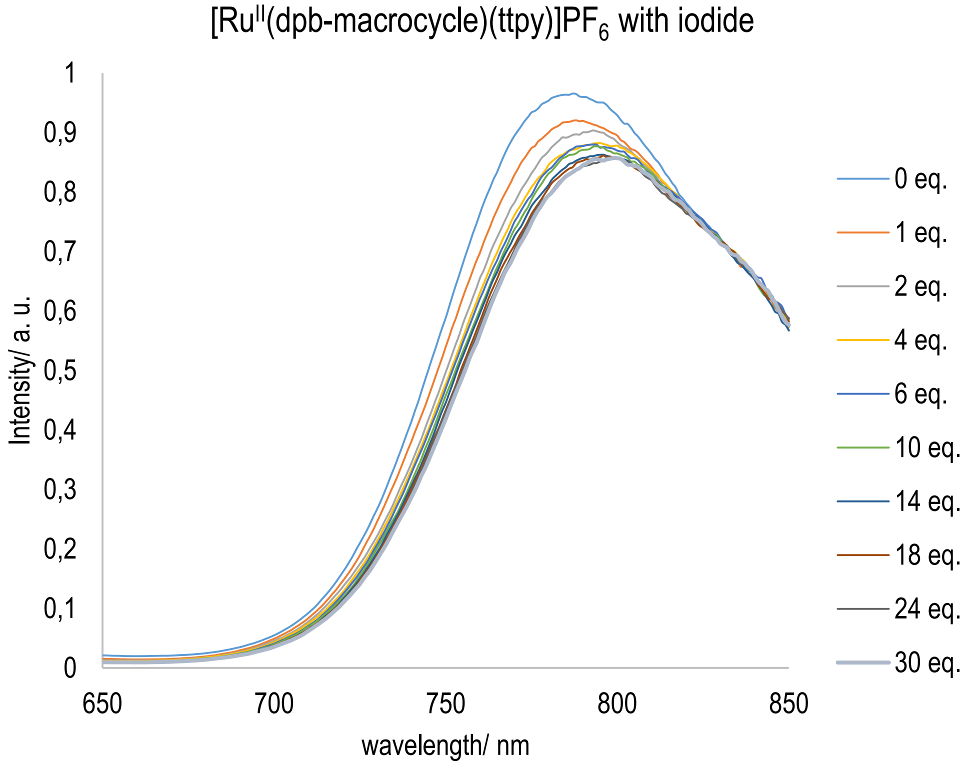

Emission spectra of [Ru^{II}(dpb-macrocycle)(ttpy)]PF_6 in the presence of increasing amounts of iodide (CH_2Cl_2/MeOH 95:5, 293 K; λ_{ex} = 530 nm)

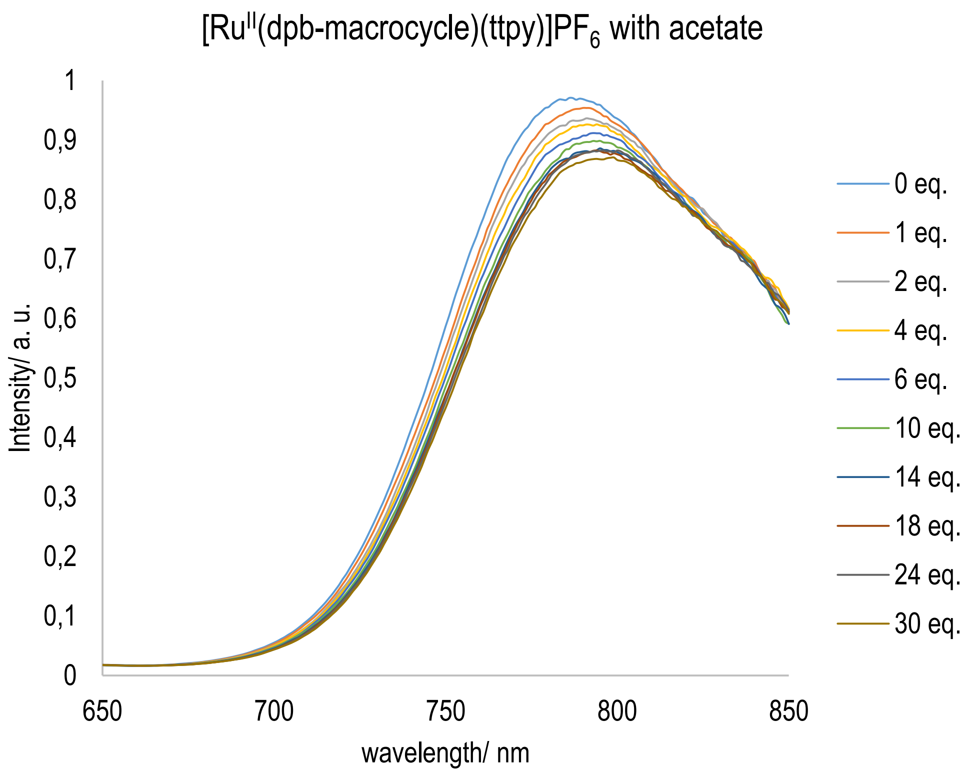

Emission spectra of $[Ru^{II}(dpb\text{-}macrocycle)(ttpy)]PF_6$ in the presence of increasing amounts of acetate (CH_2Cl_2/MeOH 95:5 , 293 K; λ_{ex} = 530 nm)

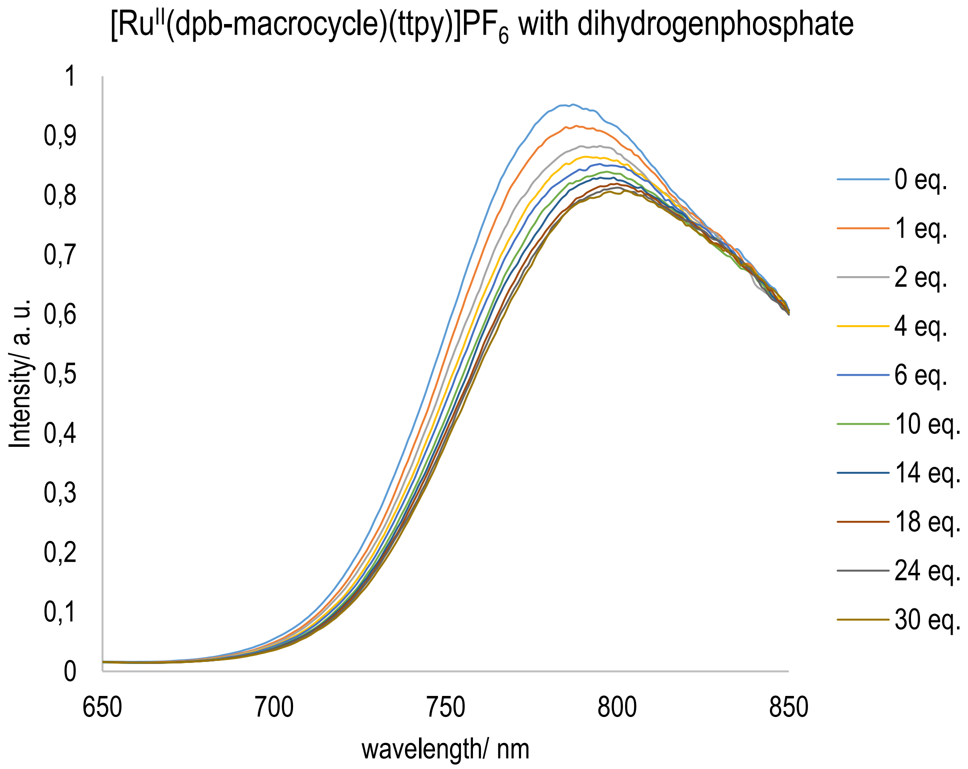

Emission spectra of $[Ru^{II}(dpb\text{-}macrocycle)(ttpy)]PF_6$ in the presence of increasing amounts of hydrogenphosphate (CH_2Cl_2/MeOH 95:5 , 293 K; λ_{ex} = 530 nm)

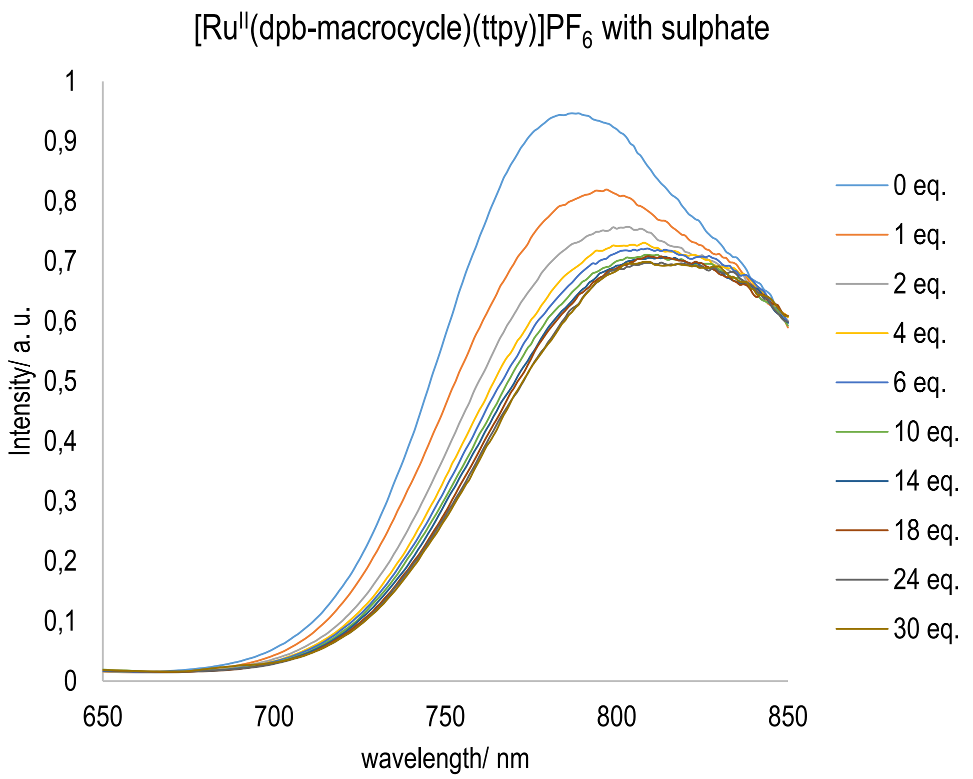

Emission spectra of [Ru^{II}(dpb-macrocycle)(ttpy)]PF_6 in the presence of increasing amounts of sulphate (CH_2Cl_2/MeOH 95:5 , 293 K; λ_{ex} = 530 nm)

2.2.5 Anion titrations with [2]rotaxane [Ru^{II}(dpb-macrocycle)(ttpy)]PF_6 12·Ru·$(PF_6)_2$

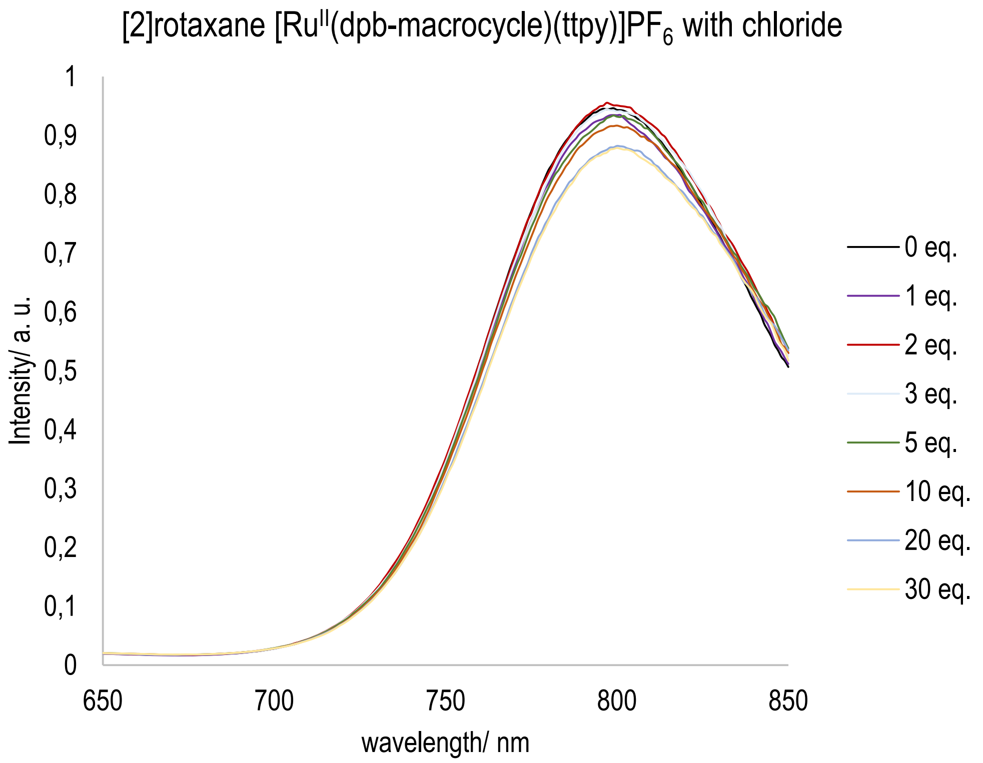

Emission spectra of [2]rotaxane [Ru^{II}(dpb-macrocycle)(ttpy)]PF_6 in the presence of increasing amounts of chloride (CH_2Cl_2/MeOH 95:5 , 293 K; λ_{ex} = 530 nm)

3. ^{1}H NMR anion titration data

Initial NMR sample volumes and concentrations were 500 μL and 2.0 mM respectively. Solutions (100 mM) of anion were added as their tetrabutylammonium salts. Spectra were recorded at 0, 0.2, 0.4, 0.6, 0.8, 1.0, 1.2, 1.4, 1.6, 1.8, 2.0, 2.5, 3.0, 4.0, 5.0, 7.0 and 10 equivalents. In all cases where association constants were calculated, bound and unbound species were found to be in fast exchange on the NMR timescale. Association constants were obtained by analysis of the resulting data using the WinEQNMR2 computer program. Binding stoichiometry was investigated by visual analysis of the titration data, and using approximations of Job plots. Estimates for the association constant and the limiting chemical shifts were added to the program's input file. The parameters were refined by non-linear least-squares analysis using WINEQNMR2[12] to achieve the best fit between observed and calculated chemical shifts. The input parameters for the final chemical shift and association constant were adjusted based on the program output until convergence was reached. Comparison of the calculated and experimental binding isotherms demonstrated that an appropriate model with an appropriate stoichiometry were used.

4. Single crystal X-ray crystallography

Data was collected at 150(2) K using either graphite monochromated Mo Kα radiation (λ = 0.71073 Å) on a Nonius KappaCCD diffractometer in the case of dimethyl-2,4-di-2-pyridyl-isophthalate ester **4**. For [Ru^{II}(dpb-hexyl)(ttpy)]Cl and *anti*- and *syn*-[Ru^{II}(dpb-macrocycle)(ttpy)]PF_6 **10·Ru·PF_6**, data were collected using silicon double crystal monochromated synchrotron radiation (λ = 0.66890 Å) at Diamond Light Source, Beamline I19 on a custom built Rigaku diffractometer.

When using the Nonius machine, a series of ω-scans were typically performed in such a way as to collect every independent reflection to a maximum resolution of 0.77 Å, aiming for 99.5% completeness. Cell parameters and intensity data (including inter-frame scaling) were processed using the DENZO-SMN package. When using synchrotron radiation, ω-scans were performed such that a half-sphere of data was collected to a maximum resolution of 0.77 Å. Cell refinement, data reduction and scaling were performed using the CrystalClear package.[13]

The structures were solved by direct methods using the SIR92 software[14] or by charge flipping using Superflip.[15] Structures were refined using full-matrix least-squares on F^2 within the CRYSTALS suite.[16] All non-hydrogen atoms were refined with anisotropic displacement parameters unless specified otherwise. Disordered portions were modelled using refined partial occupancies. Geometric and vibrational restraints were applied where appropriate to ensure physically reasonable models.

In some cases, the molecular structure within solvent voids could not be resolved in the difference map and PLATON SQUEEZE[17] was therefore used to account for the residual electron density in the refinement.

After the construction of a stable, physically reasonable and complete model, the weights were optimised,[18] analogous reflections were omitted and absent high-angle data (in the case of poorly diffracting samples) were pruned using the Wilson plot. This generally led to convergence of the refinement, giving the final structure.

For the more challenging structures, in which the refinement did not converge immediately, initially half-shifts, then restraints and finally rigid body refinement were used to overcome the problem. IUCr CheckCIF/PLATON[19] was used to validate the structures and warnings were dealt with as appropriate or justified using validation reply forms.

dimethyl-2,4-di-2-pyridyl-isophthalate ester 4 (CCDC 1978092)

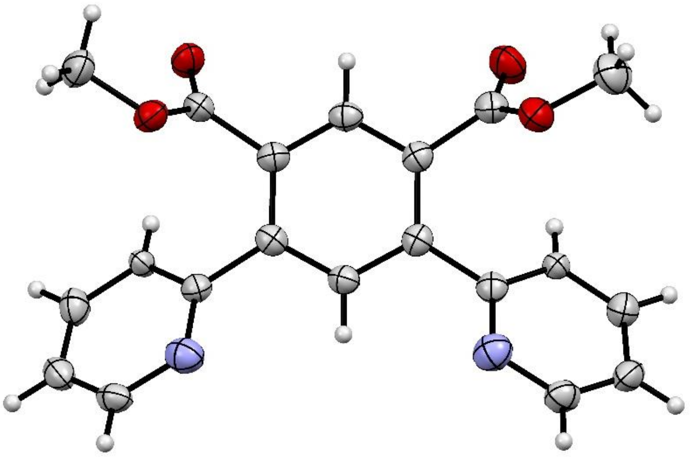

Single crystal X-ray structure of dimethyl-2,4-di-2-pyridyl-isophthalate ester (ellipsoids are plotted at the 50% probability level)

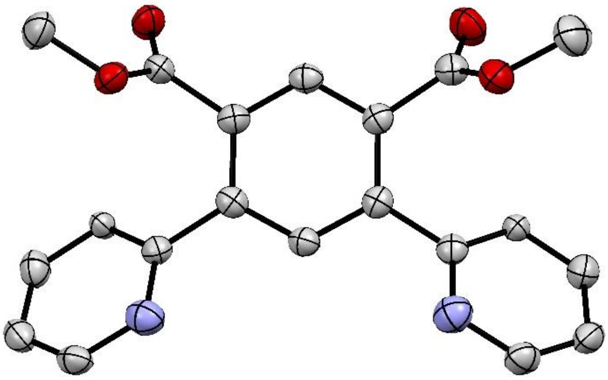

Single crystal X-ray structure of dimethyl-2,4-di-2-pyridyl-isophthalate ester (ellipsoids are plotted at the 50% probability level; hydrogen atoms omitted for clarity)

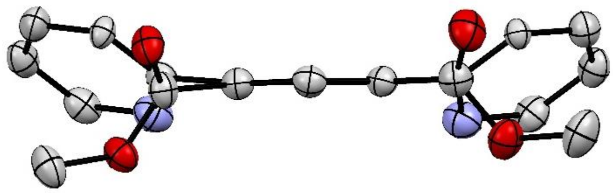

Single crystal X-ray structure of dimethyl-2,4-di-2-pyridyl-isophthalate ester (ellipsoids are plotted at the 50% probability level; hydrogen atoms omitted for clarity)

CCDC 1978092 contains the supplementary crystallographic data for this paper. These can be obtained free of charge from the Cambridge Crystallographic Data Centre *via* www.ccdc.cam.ac.uk/data_request/cif.

Single crystals of were grown from vapour diffusion of methanol into a chloroform solution of the compound over several days.

**Compound reference**	**dimethyl-2,4-di-2-pyridyl-isophthalate ester**
Chemical formula	$C_{20}H_{16}N_2O_4$
Formula Mass	348.36
Crystal system	Monoclinic
a/Å	7.0471(3)
b/Å	10.9564(4)
c/Å	11.2964(5)
α/°	73.2309(16)
β/°	89.9992(15)
γ/°	90.0079(19)
Unit cell volume/Å^3	835.11(6)
Temperature/K	150
Space group	*P*2111
No. of formula units per unit cell, *Z*	2
No. of reflections measured	6494
No. of independent reflections	3704
R_{int}	0.027
Final R_1 values ($I > 2\sigma(I)$)	0.0670
Final $wR(F^2)$ values ($I > 2\sigma(I)$)	0.1723
Final R_1 values (all data)	0.0744
Final $wR(F^2)$ values (all data)	0.1835

[Ru^{II}(dpb-hexyl)(ttpy)]Cl 7·Ru·PF_6 (CCDC 1978093)

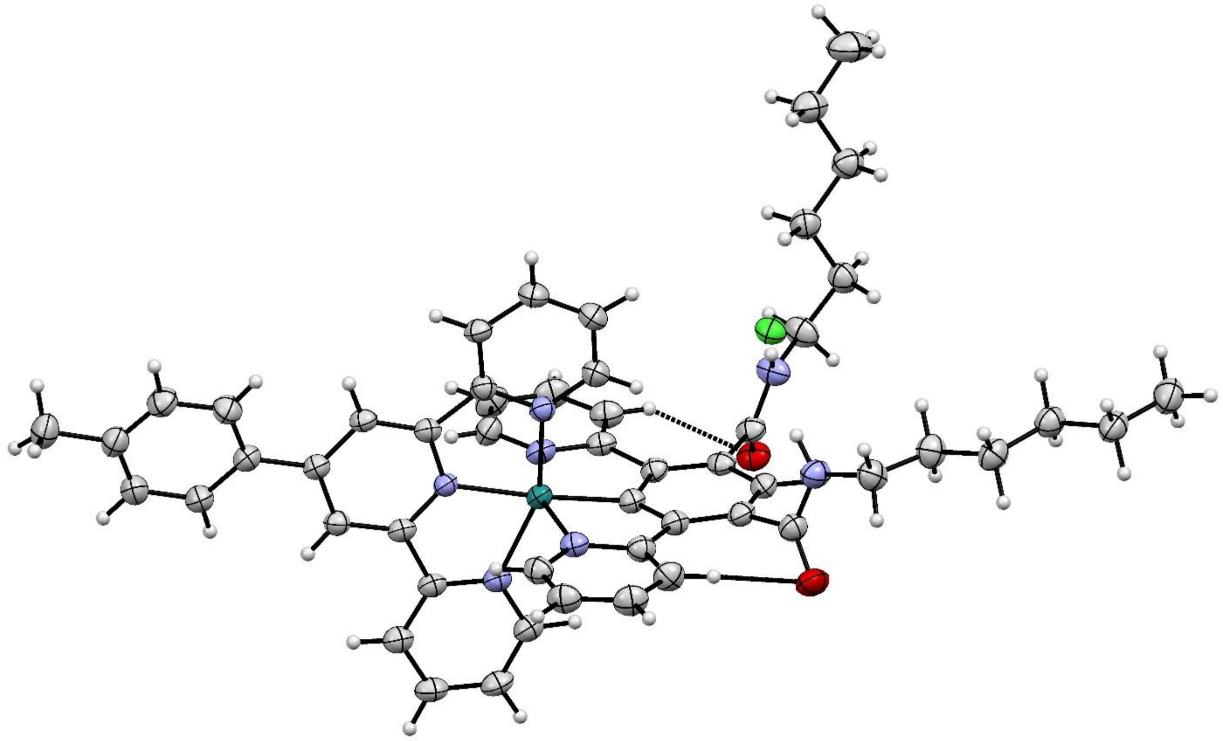

Single crystal X-ray structure of [Ru^{II}(dpb-hexyl)(ttpy)]Cl (ellipsoids are plotted at the 50% probability level)

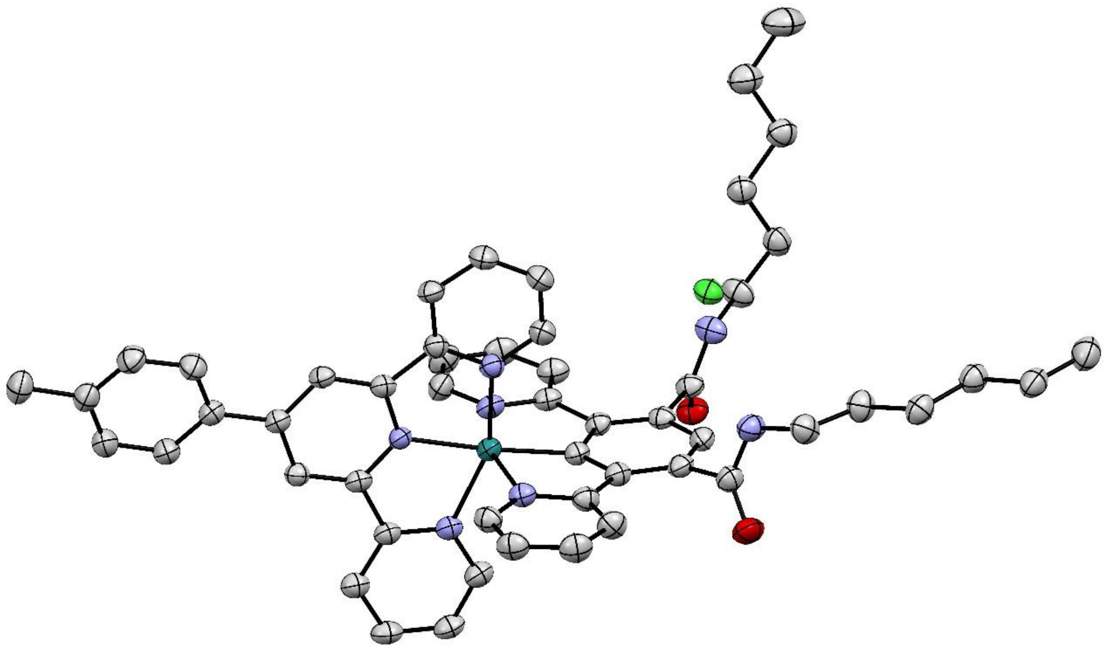

Single crystal X-ray structure of [Ru^{II}(dpb-hexyl)(ttpy)]Cl (ellipsoids are plotted at the 50% probability level; hydrogen atoms omitted for clarity)

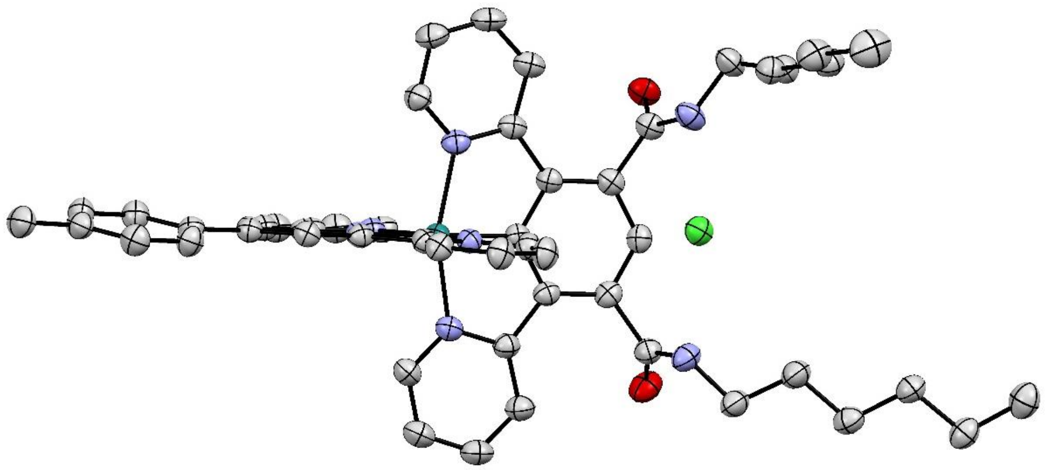

Single crystal X-ray structure of [Ru^{II}(dpb-hexyl)(ttpy)]Cl (ellipsoids are plotted at the 50% probability level; hydrogen atoms omitted for clarity)

CCDC 1978093 contains the supplementary crystallographic data for this paper. These can be obtained free of charge from the Cambridge Crystallographic Data Centre *via* www.ccdc.cam.ac.uk/data_request/cif.

Single crystals of were grown from vapour diffusion of diisopropyl ether into an acetonitrile solution of the compound in the presence of excess tetra-n-butyl ammonium chloride over several days.

Compound reference	[Ru^{II}(dpb-hexyl)(ttpy)]Cl
Chemical formula	$C_{52}H_{54}N_7O_2RuCl$
Formula Mass	945.57
Crystal system	Triclinic
a/Å	11.9614(5)
b/Å	13.3167(7)
c/Å	16.8542(6)
α/°	70.539(4)
β/°	79.249(3)
γ/°	65.151(5)
Unit cell volume/Å^3	2293.5(2)
Temperature/K	150
Space group	*P*1,¯
No. of formula units per unit cell, Z	2
No. of reflections measured	36392
No. of independent reflections	14727
R_{int}	0.049
Final R_1 values ($I > 2\sigma(I)$)	0.0554
Final $wR(F^2)$ values ($I > 2\sigma(I)$)	0.1293
Final R_1 values (all data)	0.0687
Final $wR(F^2)$ values (all data)	0.1469

***syn*-[Ru^{II}(dpb-macrocycle)(ttpy)]PF_6 10·Ru·PF_6 (CCDC 1978096)**

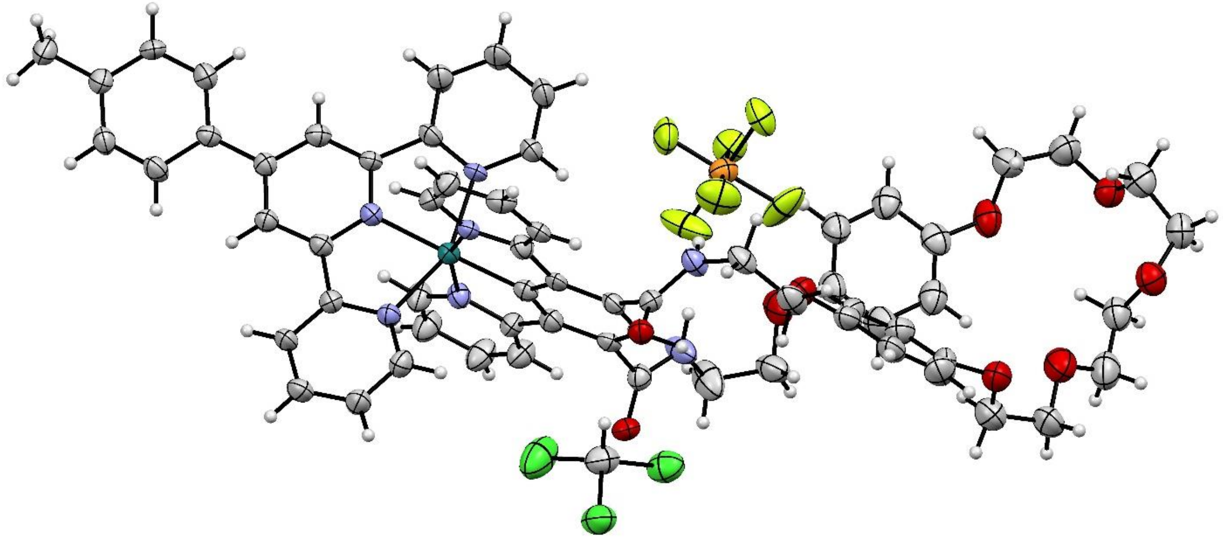

Single crystal X-ray structure of *syn*-[Ru^{II}(dpb-macrocycle)(ttpy)]PF_6 (ellipsoids are plotted at the 50% probability level; disorder omitted for clarity)

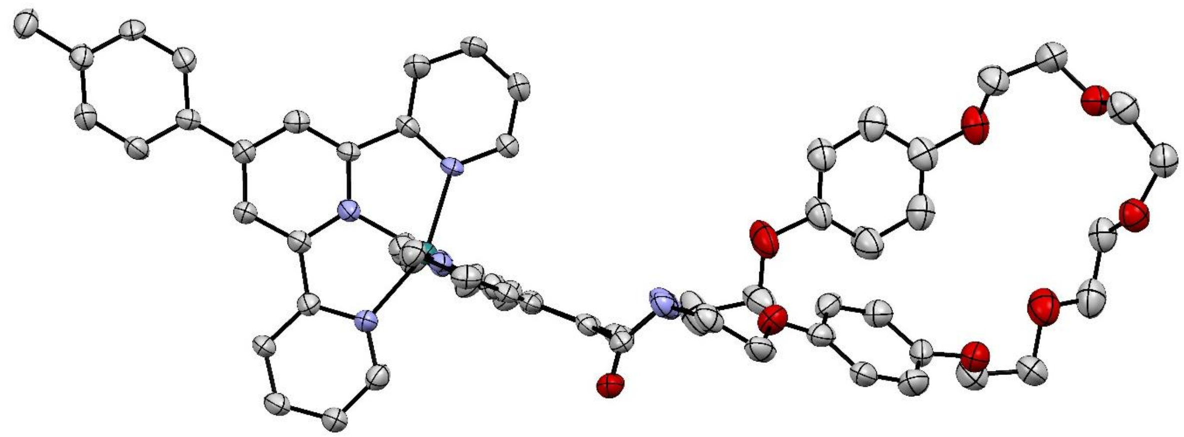

Single crystal X-ray structure of *syn*-[Ru^{II}(dpb-macrocycle)(ttpy)]PF_6] (ellipsoids are plotted at the 50% probability level; disorder, solvent and hydrogen atoms omitted for clarity)

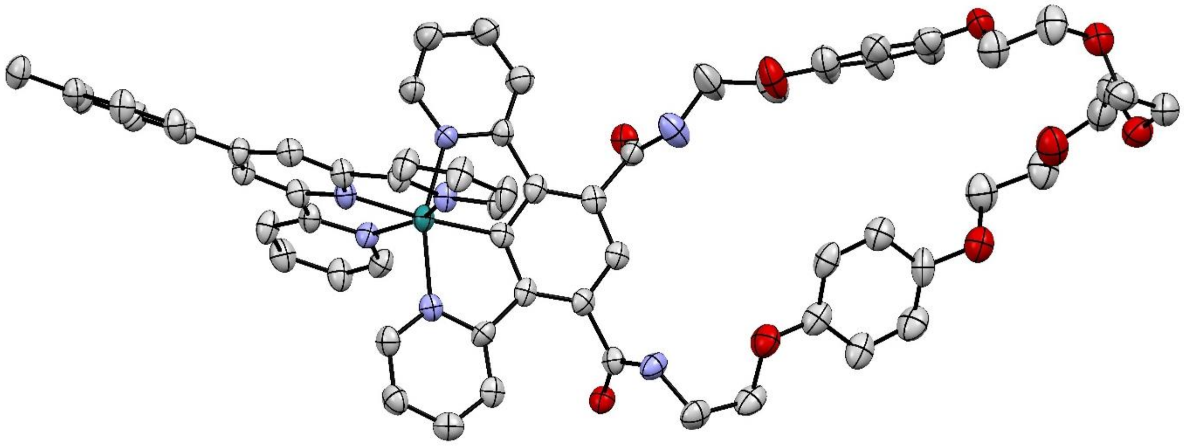

Single crystal X-ray structure of *syn*-[Ru^{II}(dpb-macrocycle)(ttpy)]PF_6 (ellipsoids are plotted at the 50% probability level; disorder, solvent and hydrogen atoms omitted for clarity)

CCDC 1978096 contains the supplementary crystallographic data for this paper. These can be obtained free of charge from the Cambridge Crystallographic Data Centre *via* www.ccdc.cam.ac.uk/data_request/cif.

Single crystals of were grown from vapour diffusion of diisopropyl ether into a chloroform solution of the compound over several days.

**Compound reference**	***syn*-[Ru^{II}(dpb-macrocycle)(ttpy)]PF_6**
Chemical formula	$C_{64}H_{60}N_7O_9Ru{\cdot}F_6P{\cdot}CHCl_3$
Formula Mass	1436.6
Crystal system	Triclinic
a/Å	11.6196(4)
b/Å	15.1032(6)
c/Å	19.5089(9)
α/°	95.892(3)
β/°	96.002(3)
γ/°	90.684(3)
Unit cell volume/Å^3	3386.0(2)
Temperature/K	100
Space group	$P1,\bar{}$
No. of formula units per unit cell, Z	2
No. of reflections measured	11024
No. of independent reflections	10271
R_{int}	0.079
Final R_1 values ($I > 2\sigma(I)$)	0.0758
Final $wR(F^2)$ values ($I > 2\sigma(I)$)	0.1902
Final R_1 values (all data)	0.0913
Final $wR(F^2)$ values (all data)	0.1859

***anti*-[Ru^{II}(dpb-macrocycle)(ttpy)]PF_6 10·Ru·PF_6 (CCDC 1978101)**

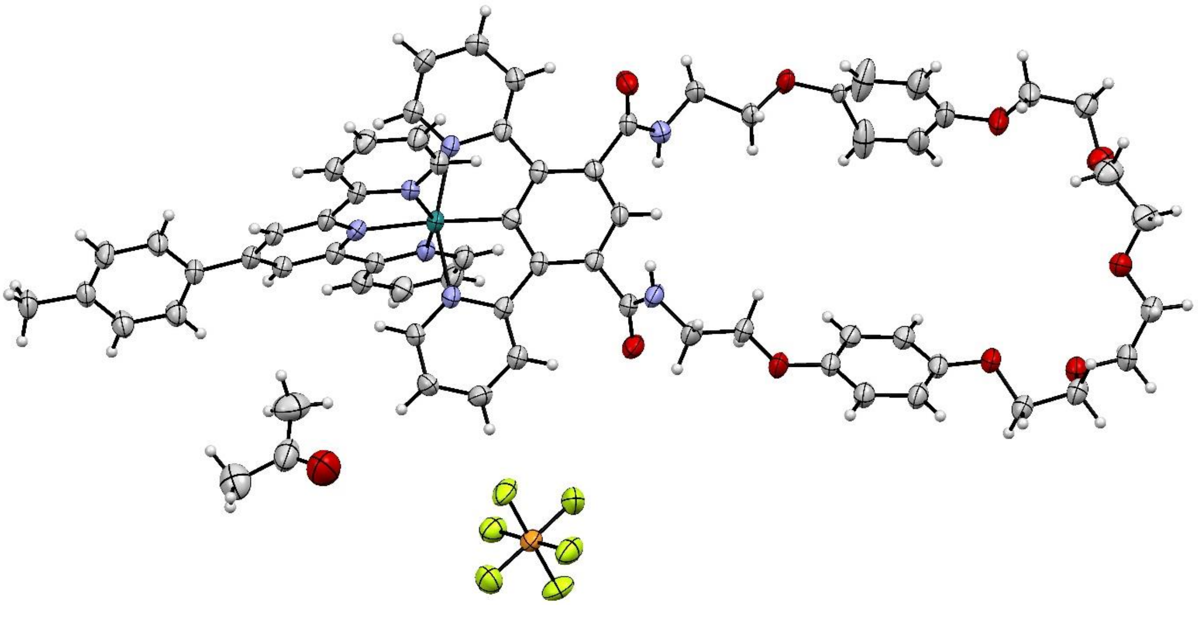

Single crystal X-ray structure of *anti*-[Ru^{II}(dpb-macrocycle)(ttpy)]PF_6 (ellipsoids are plotted at the 50% probability level; disorder omitted for clarity)

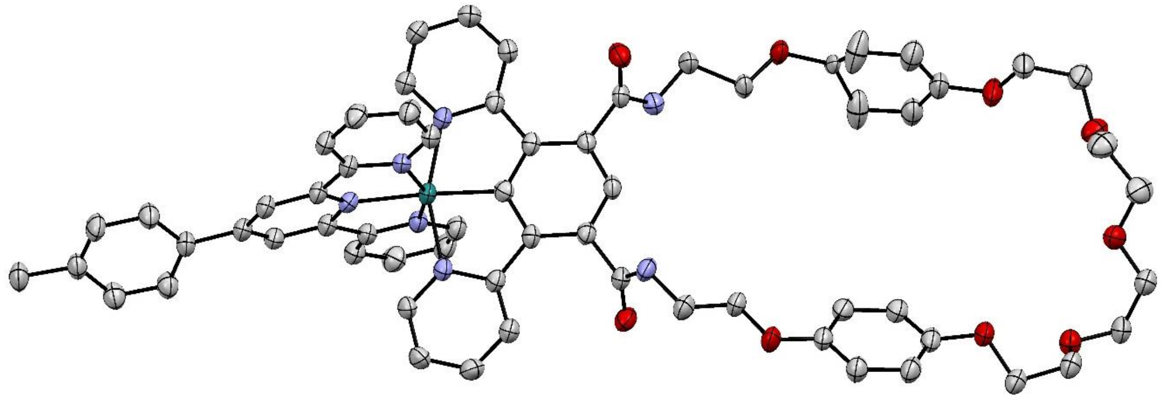

Single crystal X-ray structure of *anti*-[Ru^{II}(dpb-macrocycle)(ttpy)]PF_6] (ellipsoids are plotted at the 50% probability level; disorder, solvent and hydrogen atoms omitted for clarity)

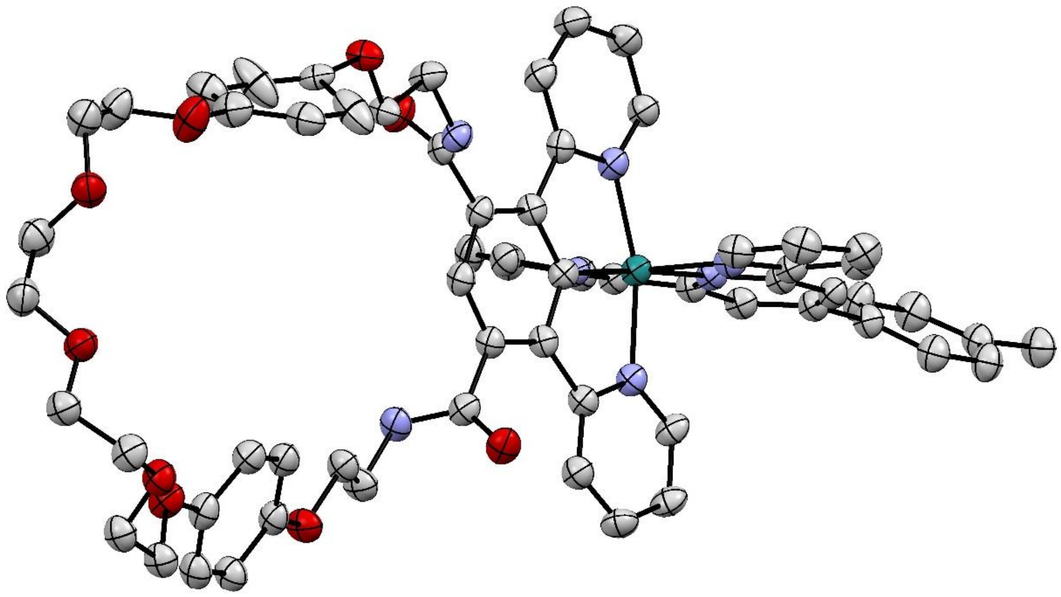

Single crystal X-ray structure of *anti*-[Ru^{II}(dpb-macrocycle)(ttpy)]PF_6 (ellipsoids are plotted at the 50% probability level; disorder, solvent and hydrogen atoms omitted for clarity)

CCDC 1978101 contains the supplementary crystallographic data for this paper. These can be obtained free of charge from the Cambridge Crystallographic Data Centre *via* www.ccdc.cam.ac.uk/data_request/cif.

Single crystals of were grown from vapour diffusion of diisopropyl ether into an acetone/H_2O (9:1) solution of the compound over several days.

Compound reference	*anti*-[Ru^{II}(dpb-macrocycle)(ttpy)]PF_6
Chemical formula	$C_{64}H_{56}N_7O_9Ru\cdot F_6P\cdot C_3H_6O$
Formula Mass	1373.30
Crystal system	Triclinic
a/Å	10.4341(5)
b/Å	12.3603(5)
c/Å	24.9734(12)
α/°	80.884(4)
β/°	84.607(4)
γ/°	76.012(4)
Unit cell volume/Å^3	3080.6(3)
Temperature/K	100
Space group	$P1$,¯
No. of formula units per unit cell, Z	2
No. of reflections measured	25967
No. of independent reflections	17475
R_{int}	0.041
Final R_1 values ($I > 2\sigma(I)$)	0.0491
Final $wR(F^2)$ values ($I > 2\sigma(I)$)	0.0915
Final R_1 values (all data)	0.0660
Final $wR(F^2)$ values (all data)	0.1029

5. References

1 A. J. Wilkinson, H. Puschmann, J. A. K. Howard, C. E. Foster and J. A. G. Williams, *Inorg. Chem.*, 2006, **45**, 8685–8699.

2 H. Nierengarten, J. Rojo, E. Leize, J.-M. Lehn and A. Van Dorsselaer, *Eur. J. Inorg. Chem.*, 2002, **2002**, 573–579.

3 A. Winter, A. M. J. van den Berg, R. Hoogenboom, G. Kickelbick and U. S. Schubert, *Synthesis (Stuttg).*, 2007, **2007**, 642.

4 Y. Halpin, D. Dini, H. M. Younis Ahmed, L. Cassidy, W. R. Browne and J. G. Vos, *Inorg. Chem.*, 2010, **49**, 2799–2807.

5 L. M. Hancock and P. D. Beer, *Chem. – A Eur. J.*, 2009, **15**, 42–44.

6 J. A. Wisner, P. D. Beer and M. G. B. Drew, *Angew. Chemie Int. Ed.*, 2001, **40**, 3606–3609.

7 J. A. Wisner, P. D. Beer, M. G. B. Drew and M. R. Sambrook, *J. Am. Chem. Soc.*, 2002, **124**, 12469–12476.

8 A. Borissov, I. Marques, J. Y. C. Lim, V. Félix, M. D. Smith and P. D. Beer, *J. Am. Chem. Soc.*, 2019, **141**, 4119–4129.

9 Y. Li, K.-W. Huang, Z. Sun, R. D. Webster, Z. Zeng, W. Zeng, C. Chi, K. Furukawa and J. Wu, *Chem. Sci.*, 2014, **5**, 1908–1914.

10 H. Gampp, M. Maeder, C. J. Meyer and A. D. Zuberbühler, *Talanta*, 1985, **32**, 95–101.

11 H. Gampp, M. Maeder, C. J. Meyer and A. D. Zuberbu¨hler, *Talanta*, 1985, **32**, 257–264.

12 M. J. Hynes, *J. Chem. Soc. Dalt. Trans.*, 1993, 311–312.

13 . Rigaku (2007), *CrystalClear. Version 1.3.5*, Tokyo, Japan.

14 A. Altomare, G. Cascarano, C. Giacovazzo and A. Guagliardi, *J. Appl. Crystallogr.*, 1994, **27**, 1045–1050.

15 L. Palatinus and G. Chapuis, *J. Appl. Crystallogr.*, 2007, **40**, 786–790.

16 P. W. Betteridge, J. R. Carruthers, R. I. Cooper, K. Prout and D. J. Watkin, *J. Appl. Crystallogr.*, 2003, **36**, 1487.

17 A. L. Spek, *J. Appl. Crystallogr.*, 2003, **36**, 7–13.

18 J. R. Carruthers and D. J. Watkin, *Acta Crystallogr. Sect. A*, 1979, **35**, 698–699.

19 M. Fourmigué and P. Batail, *Chem. Rev.*, 2004, **104**, 5379–5418.
